# Ni_12_ tetracubane cores with slow relaxation of magnetization and efficient charge utilization for photocatalytic hydrogen evolution†

**DOI:** 10.1039/d2tc03508a

**Published:** 2022-10-18

**Authors:** Elias Tanuhadi, Joan Cano, Samar Batool, Alexey Cherevan, Dominik Eder, Annette Rompel

**Affiliations:** Universität Wien, Fakultät für Chemie, Institut für Biophysikalische Chemie Josef-Holaubek-Platz-2 Wien 1090 Austria annette.rompel@univie.ac.at https://www.bpc.univie.ac.at; Department of Química Inorgànica/Instituto de Ciencia Molecular (ICMol), Facultat de Quimica, Universitat de València C/Catedrático Jose Beltrán 2 Paterna 46980, València Spain; TU Wien, Institute of Materials Chemistry Getreidemarkt 9 Vienna 1060 Austria alexey.cherevan@tuwien.ac.at https://www.imc.tuwien.ac.at/division_molecular_materials_chemistry/

## Abstract

We report two Ni_12_ multicubane topologies enclosed in the polyanions [Ni_12_(OH)_9_(WO_4_)_3_(PO_4_)(B-α-PW_9_O_34_)_3_]^21−^{Ni_12_W_30_} and [Ni_12_(OH)_9_(HPO_4_)_3_(PO_4_)(B-α-PW_9_O_34_)(A-α-PW_9_O_34_)_2_]^21−^{Ni_12_W_27_} that magnetically behave as Ni_12_ units clearly distinguishing them from typical Ni_4_ cubanes as shown by magnetic studies together with high field and frequency electron paramagnetic resonance (HFEPR). Beyond the unprecedented static properties, {Ni_12_W_30_} shows the unusual coexistence of slow relaxation of the magnetization and a diamagnetic ground state (*S* = 0), providing the unique opportunity of studying the essentially elusive magnetic relaxation behavior in excited states. The cubane-topology dependent activity of {Ni_12_W_30_} and {Ni_12_W_27_} as homogeneous HER photocatalysts unveils the structural key features significant for the design of photocatalysts with efficient charge utilization exemplified by high quantum yields (QY) of 10.42% and 8.36% for {Ni_12_W_30_} and {Ni_12_W_27_}, respectively.

## Abstract 2

Two Ni_12_-multicubane topologies {Ni_12_W_30_} and {Ni_12_W_27_} have been synthesized. Magnetometry and homogeneous catalytic studies probing their properties as hydrogen evolution photocatalysts clearly distinguish {Ni_12_W_30_} and {Ni_12_W_27_} from Ni_4_ cubanes.

## Graphical abstract

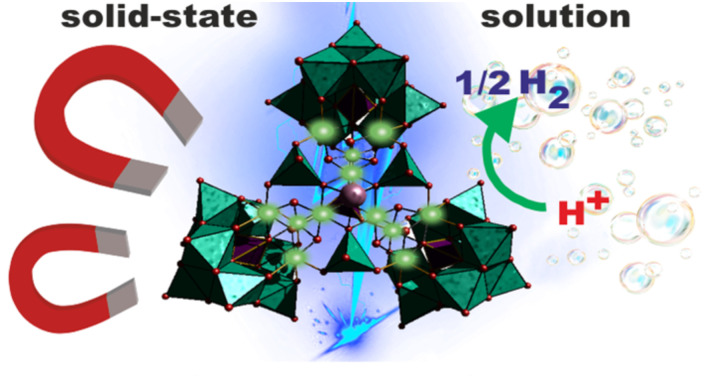

The compound class of transition metal (TM) based cubane clusters with the general formulation {TM_4_L_4_} (TM = Co^II^, Ni^II^, Fe^III^, Mn^II/III/IV^ and L = O, S, N)^1^ exhibits unique structural and electronic properties. Consequently, cubane-based materials have been subjected to magnetic studies.^2^ In solution, the structural reminiscence of enzyme active sites such as the oxygen evolving complex (OEC) {Mn_4_O_5_Ca}^3^ in photosystem II (PS II)^4,5^ and the {Fe_4_S_4_} cubane cluster in hydrogenases^6^ encouraged studies on cubane-based metal oxide nanoparticle catalysts.^7^ From a synthetic point of view, recent attention has been given to lacunary polyoxometalates (POMs).^8,9^ Being generated upon the removal of one or several MO_6_ (M = Mo^VI^, W^VI^) units from their parental architectures such as the Keggin^10^ or Wells–Dawson types^11^, lacunary POMs can act as strong inorganic, diamagnetic, multidentate O-donor ligands towards electrophiles. This multidentate nature allows the construction of mono- or multinuclear transition metal substituted POMs.^12^ Considering the lacunary POMs’ rigidity, bulkiness, and diamagnetic nature, cubane-motif incorporating POM representatives were shown to exhibit interesting magnetic properties such as single molecule magnet (SMM) behavior as a result of large spin ground state (*S*) values.^13*a*^ POMs generally exhibit a facile photoexcitation *via* near-visible or UV-light generating photoexcited species with enhanced oxidizing (higher electron affinity *E*_ea_) and reducing (lower ionization energy *E*_I_) properties.^13*b*^ Their multicentered nature allows POMs to undergo multi-redox events. In addition to their versatile redox properties, the inherent water solubility of POMs and stability encouraged researchers to employ cubane-motif incorporating POMs as homogeneous photocatalysts for the water reduction catalysis (WRC)^14^ and oxidation catalysis (WOC)^15^ reactions, respectively.

Despite the variety of existing POM-stabilized cubane motifs (Fig. S2, ESI†) that have been subjected to magnetic and/or (photo)catalytic studies (Table S1, ESI†), there are no studies exploring the correlation between a (multi)cubane-topology and its magnetic properties. Hence, advanced magnetic studies of, *e.g.*, magnetic relaxation arising from excited states that might pave the way towards advanced spintronics remain widely elusive. While significant advances have been made in studying the cubane-dependent HER photocatalytic activity of POMs^14^ (Fig. S3 and Table S2, ESI†), the underlying structural features that may grant taking full advantage of photogenerated charge carriers as a key for improving the activity of photocatalysts remain essentially unexplored.^16^

Herein, we employ phosphotungstates as rigid, all-inorganic multidentate ligands together with the simple inorganic templates PO_4_^3−^ or CO_3_^2−^ for the template-dependent stabilization of two tetracubane Ni_12_ topologies enclosed in the polyanions [Ni_12_(OH)_9_(WO_4_)_3_(PO_4_)(B-α-PW_9_O_34_)_3_]^21−^{Ni_12_W_30_} and [Ni_12_(OH)_9_(HPO_4_)_3_(PO_4_)(B-α-PW_9_O_34_)(A-α-PW_9_O_34_)_2_]^21−^{Ni_12_W_27_}. Given the identical number of incorporated Ni metal centers and their same net charge, {Ni_12_W_30_} and {Ni_12_W_27_} only differ in the type of POM-stabilized multicubane topology, thereby representing ideal candidates to study the topology dependent magnetic and photocatalytic behavior in the solid and solution states, respectively.

{Ni_12_W_30_} and {Ni_12_W_27_} were prepared employing a template dependent synthetic approach.^17*a*^ To an aqueous reaction mixture containing the K_14_[P_2_W_19_O_69_(H_2_O)]·24H_2_O^17*b*^{P_2_W_19_} lacunary precursor, three equivalents of NiCl_2_ were added following the adjustment of the pH value to 9.1 *via* CO_3_^2−^ or PO_4_^3−^ and subsequent heating of the reaction mixture at 80 °C for 10 min. Depending on the inorganic template used for the solution's basification, {Ni_12_W_30_} (CO_3_^2−^) or {Ni_12_W_27_} (PO_4_^3−^) is obtained (Fig. 1, ESI†) upon slow evaporation of the solution for two weeks at 25 °C leading to yellow-plate shaped crystals of {Ni_12_W_27_} or light-green rod-shaped crystals of {Ni_12_W_30_}, respectively. When this paper was under preparation, a crystal structure with the identical anion of {Ni_12_W_30_} was reported.^17*c*^ Note that for the synthesis of {Ni_12_W_30_}, different routes have been used by Lian *et al.* and our group (see Synthesis procedure†). The template dependent synthetic system reported in this work ultimately leads to the isolation of the novel polyanion {Ni_12_W_27_}.

**Fig. 1:**
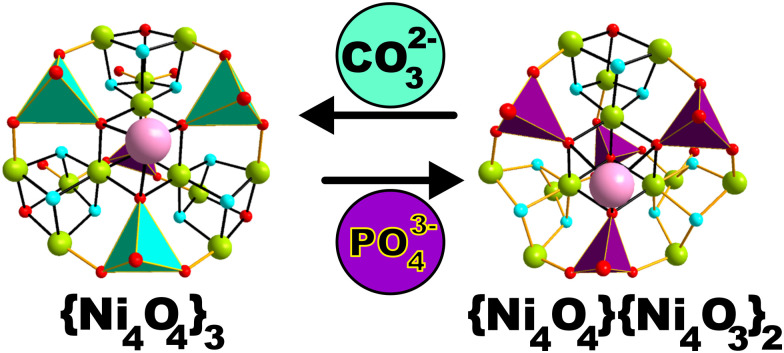
Schematic representation showing the template-dependent stabilization of the multicubane scaffolds {Ni_4_O_4_}_3_ (in {Ni_12_W_30_}*via* CO_3_^2−^) and {Ni_4_O_4_}{Ni_4_O_3_}_2_ (in {Ni_12_W_27_}*via* PO_4_^3−^. Color code, polyhedra: turquoise for {WO_4_} and purple for {PO_4_}. Balls: red for oxygen lime green for Ni^II^ and sky-blue for OH^−^ ions, respectively.

Single crystal X-ray diffraction (SXRD) was performed (Tables S5–S9, ESI†) revealing that {Ni_12_W_27_} and {Ni_12_W_30_} both incorporate a Ni_12_ metal-oxo core that differs in the type of connectivity, and resulting in multicubane topology (Fig. 1) leading to the stabilization of three full {Ni_4_O_4_} cubane units in {Ni_12_W_30_} while {Ni_12_W_27_} comprises one full {Ni_4_O_4_} and two defect {Ni_4_O_3_} cubane motifs (Fig. 1, Fig. S14 and Tables S10, S11, ESI†). The compound's elemental composition and homogeneity were determined in the solid state by elemental analysis, IR spectroscopy (Fig. S4–S9 and Table S3, ESI†), thermogravimetric analyses (TGA) (Fig. S10–S13 and Table S4, ESI†), diffuse reflectance spectroscopy (DRS) (Fig. S17–S22, ESI†), and powder X-ray diffraction (PXRD) (Fig. S15 and S16, ESI†) as well as in solution by UV/vis spectroscopy (Fig. S31, ESI†) and cyclic voltammetry (Fig. S26–S30, ESI†).

The magnetic properties of {Ni_12_W_30_} and {Ni_12_W_27_} were studied in the solid state (Fig. 2) and solution (Fig. S42, ESI†). Plots of *χ*_M_*T* (*χ*_M_ being the magnetic susceptibility per Ni_12_ unit) *vs. T* for {Ni_12_W_30_} and {Ni_12_W_27_} are displayed in Fig. 2. At 300 K, after removing the temperature-independent paramagnetism in subsequent fits (TIP = 4211 × 10^−6^ and 2756 × 10^−6^ cm^3^ mol^−1^ for {Ni_12_W_30_} and {Ni_12_W_27_}, respectively), their *χ*_M_*T* values are *ca.* 15.6 ({Ni_12_W_30_}) and 13.6 cm^3^ mol^−1^ K ({Ni_12_W_27_}). These values are larger and smaller, respectively, than the spin-only value expected (*ca.* 14.5 cm^3^ mol^−1^ K, with *g* = 2.2) for twelve magnetically non-interacting high-spin Ni^II^ ions (*S* = 1). Hence, ferromagnetic (F) interactions are suggested to be predominant in {Ni_12_W_30_} as supported by the continuous increase of the compound's *χ*_M_*T* upon lowering the temperature. In contrast to {Ni_12_W_30_}, a continuous decrease of *χ*_M_*T* starting at room temperature (300 K) and reaching a minimum at 95 K accompanied by a subsequent increase is observed for {Ni_12_W_27_}. These features support the coexistence of F and antiferromagnetic (AF) interactions, with the latter being predominant. At 31 K ({Ni_12_W_30_}, Fig. 2A) and 17 K ({Ni_12_W_27_}, Fig. 2B), *χ*_M_*T* shows maxima followed by an abrupt downturn to reach values of 5.7 and 15.0 cm^3^ mol^−1^ K at 2.0 K, respectively. Usually, zero-field splitting (zfs) could be the responsible factor for the observed decrease, as shown by the values of the local axial and rhombic zfs parameters (Table S14, see ESI,† Magnetism). The sharp downturn observed for {Ni_12_W_30_} (Fig. 2A) and {Ni_12_W_27_} (Fig. 2B) suggests additional factors apart from conventional zfs to be related to the AF inter- or intramolecular interactions. The shapes of the magnetization (*M*) *vs. H* plot at 2.0 K for both compounds (see insets of Fig. 2) show values largely below that expected for a ferromagnetic *S* = 12 spin ground state (26.4 *Nβ*, with *g* = 2.2). In the case of {Ni_12_W_27_}, weak or moderate *H* cancel the intermolecular magnetic interaction between two Ni_12_ units, and *M* tends to a saturation value of *ca* 12 *Nβ*, which is close to that expected for an *S* = 6 ground state. In contrast, a gentle increase of *M* for {Ni_12_W_30_} is observed at low fields without any trend for reaching saturation, supporting the close presence of many excited states of higher spin multiplicity that are partially populated upon increasing the magnetic field or the temperature. An estimation of the coupling constants (*J*_i_ values) obtained from DFT calculations guides the analysis of the observed magnetic behavior of both compounds suggesting a diamagnetic spin ground state *S* = 0 for {Ni_12_W_30_} with an energetically close excited state *S* = 1 (0.4 cm^−1^) and a ground state *S* = 6 with close excited states *S* = 5 and *S* = 4 at 2.0 and 10.1 cm^−1^ for {Ni_12_W_27_} (Fig. S32–S37 and Tables S12, S13, ESI,† see Magnetism).

**Fig. 2:**
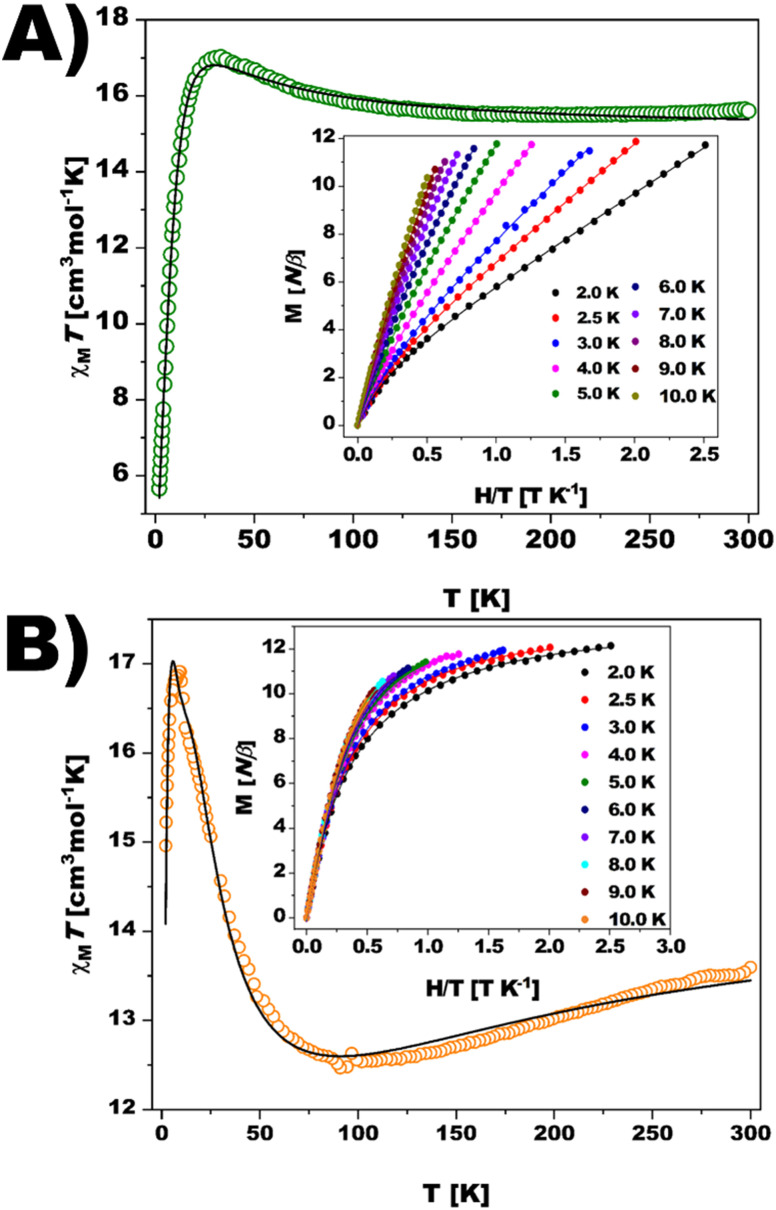
Temperature dependence of *χ*_M_*T* and reduced magnetization curves (inset) for (A) {Ni_12_W_30_} and (B) {Ni_12_W_27_}. Solid lines are the best-fit curve and a guide to the eye for *χ*_M_*T* and *M*.

Alternating current (ac) magnetic susceptibility studies of {Ni_12_W_30_} and {Ni_12_W_27_} were performed to explore their magnetic relaxation properties. Out-of-phase 
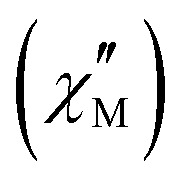
 signals were observed both in the absence and under applied dc magnetic fields (*H*_dc_) (Fig. S38 and S39, ESI†), typical for SMMs with energy barriers (*E*_a_) risen from a *D* < 0 for low rhombicity (*E*/*D* ≈ 0).^18,19^ The occurrence of only incipient 
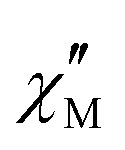
 signals without maxima in the 
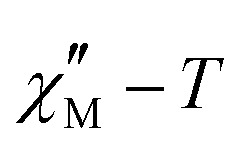
 plots in both compounds precludes a correct treatment of the experimental data. Hence, a semiquantitative estimation of the *E*_a_ values was extracted from the 
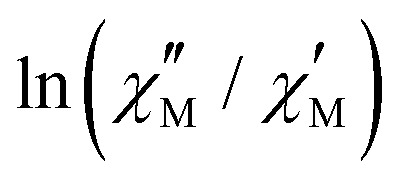
*vs.* 1/*T* plots for each frequency.^18^ According to the relationship 
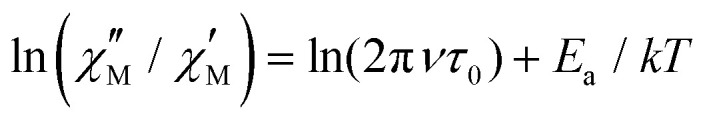
 valid for a system with a single relaxation process, a collection of parallel straight lines is expected (Fig. S40 and S41, ESI†).^18,19^ The value of *E*_a_ would be obtained from their slope, giving *E*_a_ values of 18.1(7) and 25.0(3) cm^−1^ for {Ni_12_W_30_} and {Ni_12_W_27_}, respectively, with the *E*_a_ value for {Ni_12_W_30_} being close to that estimated (17.3 cm^−1^, the largest value between the round-trip paths) from the values of *D* (−15.2 cm^−1^) and *E* (0.14) proposed from the theoretical study for the first excited state *S* = 1. Since the negative sign of *D* enables a magnetic energy barrier and the good agreement between experimental and theoretical values of *E*_a_, it can be concluded that a two-phonon Orbach mechanism governs the relaxation of the magnetization. The magnetization relaxation in {Ni_12_W_27_} occurs in the *S* = 6 ground state and is controlled through the energy barrier promoted by the zfs, whereas the diamagnetic singlet ground state *S* = 0 in {Ni_12_W_30_} suggests the observed slow relaxation of magnetization to arise from the close first excited state (*S* = 1, 0.4 cm^−1^, Fig. S36, ESI†).^20*a*,*b*^ This excited triplet state *S* = 1 is only partially populated at the lowest experimental temperature being the cause of the weak 
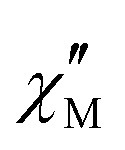
 signals and precluding the data analysis in the absence of *H*_dc_. These assumptions are additionally reinforced by high-frequency and high-field electron paramagnetic resonance (HFEPR) studies, which show an EPR-silent spectrum for {Ni_12_W_30_} in accordance with the proposed singlet ground state *S* = 0 (Fig. S43, ESI†), whereas HFEPR studies on {Ni_12_W_27_} reveal a weak signal at 6.6 T (Fig. S44 and S45, ESI†) further supporting the presence of a paramagnetic ground state and dipolar AF interactions arising from the {Ni_12_W_27_}_2_ supradimers, according to the DFT study and the crystal structure (Fig. S46, ESI,† DFT studies. Section 10.1). The observed topology-dependent magnetic behaviour and electronic properties distinguish the isolated Ni_12_ scaffolds from typical {Ni_4_O_4_} single-cubane motifs.

A careful inspection of the 236 GHz EPR spectrum of {Ni_12_W_30_} allows for identifying of two almost muted signals at 4.0 and 6.9 T resulting from a very close excited state (Fig. S43, ESI†). These signals do not change as the temperature increases, and their intensity decreases when using a frequency of 400 GHz. This surprising result must be attributed to several excited states very close to the ground one, as shown in Figure S36 (ESI†) from the theoretical study. A triplet spin state and a *D* value comparable to that proposed by CASSCF calculations (≈−15 cm^−1^) could reproduce the signal at 6.9 T. However, the low-field signal must appear due to a notable rhombicity in the zfs tensor (*E*/*D* ≠ 0), which agrees well with the theoretical study (0.14) and the weakness of these signals (page 59 in the ESI†).

The EPR spectrum at 236 GHz of {Ni_12_W_27_} shows five attenuated signals at 3.6, 6.2, 6.6, 7.0 and 8.0 T, which move to higher fields when increasing the frequency to 388 GHz but do not substantially modify their intensity (Fig. S45, ESI†). This pattern resembles that observed in a previous double-cubane Ni_7_ cluster,^20*c*^ which, like {Ni_12_W_27_}, exhibits an *S* = 6 ground state, supporting our conclusions. The analysis of the EPR spectra of Ni_7_ required a nearby *S* = 7 excited state. However, according to our theoretical study, an analogous procedure in {Ni_12_W_27_} should consider an *S* = 5 rather than an *S* = 7 excited state. Unfortunately, the weakness of the signals on {Ni_12_W_27_} prevents further investigation.

Following earlier reports on structurally relevant Ni-containing POM hydrogen evolution catalysts (HECs)^21^ the TBA salts of {Ni_12_W_30_} and {Ni_12_W_27_} were evaluated towards the visible-light-driven hydrogen evolution reaction (HER) employing [Ir(dtbbpy)(ppy)_2_]^+^ (dtbbpy = 4,4′-di-*tert*-butyl-2,2′-dipyridyl, ppy = 2-phenylpyridine) as the photosensitizer (PS), triethanolamine (TEOA) as an electron donor and 11 : 33 : 4 vol% CH_3_CN/DMF/H_2_O as a solvent mixture (see Hydrogen evolution (HER) experiments, Fig. S47 and S48, ESI†). A compositionally relevant tetra-nickel polyoxotungstate reported by Hill *et al.*^21*c*^ referred to as {Ni_4_W_18_} was prepared, characterized (Fig. S1, ESI†), and tested under identical experimental conditions as a benchmark HEC following a comprehensive set of pre-catalytic studies employing time-dependent UV/vis studies (Fig. S31, ESI†) that demonstrated pre-catalytic stability of {Ni_12_W_30_} and {Ni_12_W_27_} as shown by the compounds’ virtually unchanged spectra over a time period of 60 min relevant to the catalytic experiment (see Section S12.1, ESI†).

Table 1 contains the values of the turnover frequencies (TOFs) calculated after 10 minutes of each HER experiment. Depending on the concentrations of {Ni_12_W_30_} and {Ni_12_W_27_}, their HER TOFs lie in the range between 19.8 and 145.7 (expressed in 10^−3^ s^−1^). To allow for a valid comparison to relevant catalysts, the HER quantum yields (QYs, see General information†) were calculated (Table 1) and amount to 10.42 and 8.36% for {Ni_12_W_30_} and {Ni_12_W_27_}, respectively. These QYs greatly surpass values previously reported for Ni-containing POMs^14,16^ (Table S2, ESI†) and – considering that HER QY values already include absorption and recombination losses (*e.g.*, contributions by PS-POM charge transfer) – they indicate extremely high efficiency of charge utilization for {Ni_12_W_30_} and {Ni_12_W_27_} suggesting {Ni_12_W_30_} and {Ni_12_W_27_} to be the fastest Ni-PT based HECs reported so far (Table S2, ESI†).

**Table 1:** Average values (standard deviations 3–6%) of turnover number (TON) and turnover frequency (TOF) values towards H_2_ generation for {Ni_12_W_30_}, {Ni_12_W_27_}, the benchmark {Ni_4_W_18_} and Ni(NO_3_)_2_ along with quantum yields (QYs) (2–20 μM). TONs were extracted after H_2_ evolution saturation point (point *x* at 60 min), initial TOFs and QYs were extracted after 10 min (Fig. 3)

Ni-POT	*c*/μM	H_2_/μmol	TON	TOF/10^−3^ s^−1^	QY/%
{Ni_12_W_30_}	2	0.56	140.9	145.7	6.15
	5	0.82	81.6	84.4	8.90
	10	1.11	55.4	39.2	8.26
	20	1.47	36.7	24.7	10.42
{Ni_12_W_27_}	2	0.52	130.0	106.3	4.49
	5	0.81	81.0	56.1	5.92
	10	1.12	56.2	37.4	7.89
	20	1.55	38.8	19.8	8.36
{Ni_4_W_18_}	20	0.45	11.4	8.8	3.72
Ni(NO_3_)_2_	20	0.35	8.7	6.9	

**Fig. 3:**
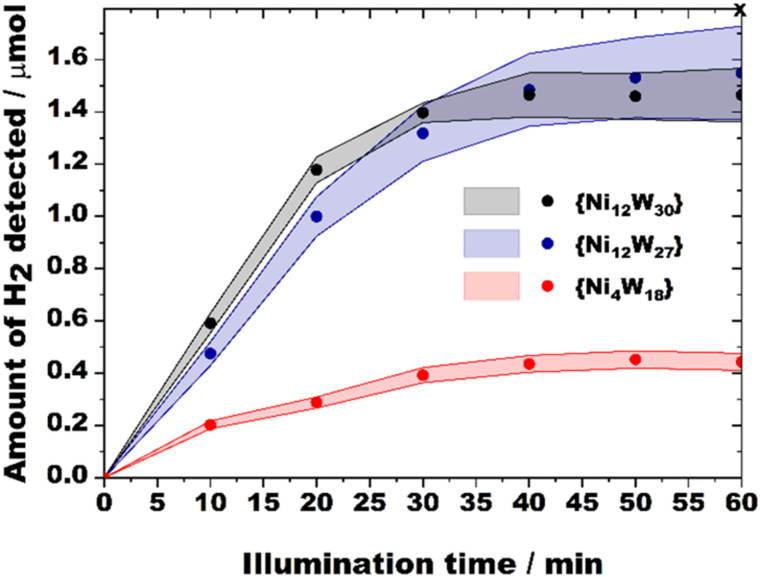
H_2_ evolution profiles for {Ni_12_W_30_}, {Ni_12_W_27_}, and the benchmark {Ni_4_W_18_} obtained using 20 μM catalyst concentrations. Data points represent average values obtained from multiple measurements, and colored areas indicate standard deviations for each compound and data point. Each measurement was conducted for 60 min until H_2_ saturation was reached (point *x*).

Post-catalytic studies (see the ESI† section Post-catalytic studies) featuring IR-spectroscopy demonstrated the post-catalytic bulk stability of {Ni_12_W_30_} and {Ni_12_W_27_} (Fig. S49, ESI†), which was subsequently assessed quantitatively employing X-ray fluorescence (XRF) (Table S15, ESI†). A series of reloading experiments demonstrated the recyclability of {Ni_12_W_30_} and {Ni_12_W_27_} as homogeneous HECs (Fig. S50, ESI†).

A careful analysis of the TOF values allows for structure–activity relationship (SAR) correlations between {Ni_12_W_30_} and {Ni_12_W_27_} that both feature {Ni^II^_4_O_4_} quasi-cubanes. While higher catalyst concentrations (10 and 20 μM) yield similar TOFs, a significantly higher HER performance of {Ni_12_W_30_} over {Ni_12_W_27_} can be observed for 2 and 5 μM catalyst solutions at which the catalytic role of the elsewise isostructural Ni-PTs – that both display the same net charge of −21 – is more pronounced. One contribution to the superior activity of {Ni_12_W_30_} over {Ni_12_W_27_} can be related to the higher number of {Ni^II^_4_O(OH)_3_} cubanes incorporated in the POT framework as compared to {Ni_12_W_27_}, which is in accordance with the findings reported by Wang and co-workers (Fig. S3, ESI†).^14^

To further explore the origin of the higher HER activity of {Ni_12_W_30_}, photoluminescence (PL) emission studies (Fig. S52 and S53, Section S14, ESI†) elucidating the HEC mechanism were conducted. Both steady-state and time-resolved data propose reductive quenching to dominate under the turnover conditions (Scheme S1, ESI†), which suggests that the reversible reduction of the corresponding Ni-PT (Scheme S2, ESI,† step III) – which consecutively reduces H^+^ upon H_2_ formation may represent a rate-limiting step in the HER. Based on CV studies on {Ni_12_W_27_} (Fig. S25, ESI†), {Ni_12_W_30_} (Fig. S24, ESI†), and {Ni_4_W_18_} (Fig. S23, ESI†), the onset reduction of the Ni-PTs occurs at more positive potentials in the order {Ni_4_W_18_} (−0.66 V) < {Ni_12_W_27_} (−0.25 V) < {Ni_12_W_30_} (−0.12 V). Hence, the observed activity trend can be explained by the increased tendency of {Ni_12_W_30_} to be reduced, which is additionally reflected by a substantially higher (50%) quenching rate *K*_q_ for {Ni_12_W_30_} (*K*_q_ = 13.2 × 10^9^ M^−1^ s^−1^) compared to that of {Ni_12_W_27_} (*K*_q_ = 8.9 × 10^9^ M^−1^ s^−1^) (Fig. S53, ESI†) illustrating its more efficient redox shuttling. A template-dependent change in the topology of the Ni_12_ metal-oxo core resulted in an increased number of {Ni^II^_4_O(OH)_3_} cubanes present in {Ni_12_W_30_} and provided this Ni-PT with an enhanced charge-utilization expressed by the tuned redox-activity and increased photocatalytic HER performance as compared to {Ni_12_W_27_} and {Ni_4_W_18_}.

## Conclusions

Our results highlight that a diamagnetic ground state does not necessarily preclude SMM behavior in compounds that exhibit energetically closely located paramagnetic excited spin states. Moreover, incorporating a tandem ground and nearby excited states, behaving both as SMMs or qubits, allows for envisaging advanced spintronics and quantum computing. Structure–activity studies on {Ni_12_W_30_} and {Ni_12_W_27_} probing their photocatalytic HEC activity revealed high charge utilization for {Ni_12_W_30_} and {Ni_12_W_27_}. The topology modulation of the tetracubane scaffold employing simple inorganic anionic templates represents a key to tuning their functional HEC properties upon modulating the corresponding Ni-PT's redox properties as exemplified by the high quantum yield of {Ni_12_W_30_}.

## Conflicts of interest

There are no conflicts to declare.

## Supplementary Material

TC-010-D2TC03508A-s001

TC-010-D2TC03508A-s002
