# Supplementary material for: Ni12 tetracubane cores with slow relaxation of magnetization and efficient charge utilization for photocatalytic hydrogen evolution
Source: J Mater Chem C Mater. 2022 Oct 18;10(45):17048–52. doi: 10.1039/d2tc03508a (PMC9686624; doi:10.1039/d2tc03508a)
Supplement: TC-010-D2TC03508A-s001 [file TC-010-D2TC03508A-s001.pdf]

This journal is © The Royal Society of Chemistry 2022

ELECTRONIC SUPPLEMENTARY INFORMATION

Ni_{12} - tetracubane cores with slow relaxation of magnetization and efficient charge utilization for photocatalytic hydrogen evolution

Elias Tanuhadi†, Joan Cano‖, Samar Batool‡, Alexey Cherevan*,‡, Dominik Eder‡, and Annette Rompel*,†

*correspondence to: annette.rompel@univie.ac.at, alexey.cherevan@tuwien.ac.at

† Universität Wien, Fakultät für Chemie, Institut für Biophysikalische Chemie, Josef-Holaubek-Platz-2, 1090 Wien, Austria; www.bpc.univie.ac.at

‖ Department of Química Inorgànica/Instituto de Ciencia Molecular (ICMol), Facultat de Quimica de la Universitat de València, C/Catedrático Jose Beltrán 2, 46980 Paterna, València, Spain

‡ TU Wien Institute of Materials Chemistry, Getreidemarkt 9, Vienna, 1060, Austria; https://www.tuwien.at/en/tch/mmc

Content

1. General Information **3**

2. Synthesis Procedure **6**

2.1. Synthesis of $K_{11}Na_{10}[Ni_{12}(OH)_9(WO_4)_3(PO_4)(B\text{-}\alpha\text{-}PW_9O_{34})_3]$ • 98 H_2O ($K_{11}Na_{10}$-**{$Ni_{12}W_{30}$}**) 6

2.2. Ion-exchange procedure – preparation of $(C_{16}H_{36}N)_{13}Na_8[Ni_{12}(OH)_9(WO_4)_3(PO_4)(B\text{-}\alpha\text{-}PW_9O_{34})_3]$ • 13 H_2O ($TBA_{13}Na_8$-**{$Ni_{12}W_{30}$}**) 7

2.3. Synthesis of $K_{14}Na_7[Ni_{12}(OH)_9(HPO_4)_3(PO_4)(B\text{-}\alpha\text{-}PW_9O_{34})(A\text{-}\alpha\text{-}PW_9O_{34})_2]$ • 44 H_2O ($K_{14}Na_7$-**{$Ni_{12}W_{27}$}**) 7

2.4. Ion-exchange procedure – preparation of $(C_{16}H_{36}N)_{13}Na_8[Ni_{12}(OH)_9(HPO_4)_3(PO_4)(B\text{-}\alpha\text{-}PW_9O_{34})(A\text{-}\alpha\text{-}PW_9O_{34})_2]$ • 11 H_2O ($TBA_{13}Na_8$-**{$Ni_{12}W_{27}$}**) 7

3. IR-spectra **18**

4. Thermogravimetric analysis **23**

5. Single-Crystal X-ray Diffraction (SXRD) **26**

6. Powder X-ray Diffraction (PXRD) **44**

7. Optical transition **46**

7.1. Estimation of E_g using diffuse reflectance spectroscopy (DRS) 46

7.2. Estimation of band gap position using cyclic voltammetry (CV) 49

8. Cyclic voltammetry **52**

9. UV/vis spectroscopy **55**

10. Magnetism **56**

10.1. DFT - guided estimation of coupling constants (J_i values) 56

10.2. Magnetic behavior of $K_{11}Na_{10}$-**{$Ni_{12}W_{30}$}** and $K_{14}Na_7$-**{$Ni_{12}W_{27}$}** in solution 73

11. High-Frequency/High-Field Electron Paramagnetic Resonance (HFEPR) **74**

12. Hydrogen Evolution (HER) experiments **78**

12.1. Pre-catalytic stability study. 78

12.2. HER – activity studies on $TBA_{13}Na_8$-**{$Ni_{12}W_{30}$}** and $TBA_{13}Na_8$-**{$Ni_{12}W_{27}$}**. 78

13. POM - HER activity and integrity **81**

13.1 Post-catalytic studies 81

13.2 Post-catalytic precipitation of $TBA_{13}Na_8$-**{$Ni_{12}W_{30}$}** and $TBA_{13}Na_8$-**{$Ni_{12}W_{27}$}** 81

13.3 Reloading experiments 82

13.4 Total X-ray fluorescence (TXRF) experiments 82

14. Photoluminescence (PL) emission spectroscopy **84**

15. References **87**

1. General Information

All reagents and chemicals were of high-purity grade and were used as purchased without further purification. $K_{14}[P_2W_{19}O_{69}(H_2O)] \cdot 24H_2O$ (**{P$_2$W$_{19}$}**) and $(TBA)_3K_4Na_3[Ni_4(H_2O)_2(PW_9O_{34})_2]$ (**{Ni$_4$W$_{18}$}**) were prepared according to literature procedures[1] and characterized using single-crystal X-ray diffraction (SXRD) (**{Ni$_4$W$_{18}$}**) and ^{31}P-NMR spectroscopy (**{P$_2$W$_{19}$}**) as well as ESI-MS (**{Ni$_4$W$_{18}$}**) (**Figure S1**).

Attenuated total reflection Fourier−transform Infrared Spectroscopy: All FTIR spectra were recorded on a Bruker Vertex 70 IR Spectrometer equipped with a single−reflection diamond−ATR unit. Frequencies are given in cm^{-1}, intensities denoted as w = weak, m = medium, s = strong, br = broad.

Elemental analysis: Elemental analysis was performed using X-ray photoelectron spectroscopy (XPS). The analysis was performed with a Nexsa XPS system (Thermo-Fisher) using a radiation source gun-type Al K*α* operating at 72 W and a pass energy of 200 eV, a spot size of 400 µm, "Standard Lens Mode", CAE Analyzer Mode, an energy step size of 0.1 eV for the survey spectrum, and integrated flood gun. Analysis was performed after cleaning the surface with Ar-clusters (1000 atoms, 6000 eV, 1 mm raster size) for 60 s. The high-resolution C 1s spectrum was acquired with 10 passes at a pass energy of 50 eV and fitted using Thermo Avantage v5.9914, Build 06617 with Smart background and Simplex Fitting algorithm using Gauss-Lorentz Product. Elemental microanalysis of C/H/N/O contents was performed by Mikroanalytisches Laboratorium (University Vienna, Faculty of Chemistry). An EA 3000 (Eurovector) was used for C/H/N/S-analysis. O-determination was performed by high temperature digestion using the HT 1500 (Hekatech, Germany) pyrolysis system in combination with the EA 3000 system.

UV–Vis spectroscopy: UV−Vis spectra were collected on a Shimadzu UV 1800 spectrophotometer.

Thermogravimetric analysis (TGA): TGA was performed on a Mettler SDTA851e Thermogravimetric Analyzer under N_2 flow with a heating rate of 5 K min^{-1} in the region 298−973 K.

Single crystal X-ray diffraction (SXRD): The X-ray data were measured on a Bruker D8 VENTURE equipped with a multilayer monochromator, Mo *Kα* Incoatec Microfocus sealed tube, and Kryoflex cooling device. The structures were solved by direct methods and refined by full-matrix least-squares. Non hydrogen atoms were refined with anisotropic displacement parameters. The following software was used for the structure-solving procedure: frame integration, Bruker SAINT software package using a narrow-frame algorithm (absorption correction)[2], SADABS[3], SHELXS-2013[4] (structure solution), SHELXL-2013[5] (refinement), OLEX2[6] (structure solution, refinement, molecular diagrams and graphical user-interface), and SHELXLE[7] (molecular diagrams and graphical user interface). CCDC-codes are provided in **Table S5**. Experimental data are summarized in **Tables S6-S9.**

Powder X-ray diffraction was performed on an EMPYREAN diffractometer system using Cu *Kα* radiation (λ = 1.540598), a PIXcel3D-Medipix3 1 × 1 detector (used as a scanning line detector) and a divergence slit fixed at 0.1 mm. The scan range was from 5° to 50° (2θ).

Diffuse reflectance spectroscopy (DRS) was performed on a Jasco V-670 UV-Vis spectrometer using a diffuse reflectance unit containing an Ulbricht-sphere. The powdered samples were fixed in the micro sample holder with a diameter of 3 mm and $MgSO_4$ was used as a standard.

Theoretical Study: DFT calculations were carried out using the Gaussian 09 package in order to estimate the magnitude and nature of the magnetic couplings in **{Ni$_{12}$W$_{30}$}** and **{Ni$_{12}$W$_{27}$}**.[8] These calculations were performed with the B3LYP hybrid functional[9], the quadratic convergence approach, a guess function generated with the fragment tool of the same program, and a stability test of the wavefunction. All electron basis sets proposed by Ahlrichs *et al*. were used for every atom type except for tungsten, for which the LanL2DZ basis set and pseudopotentials were employed.[10] A Triple-ζ (TZV) basis set was employed for nickel atoms, whereas the double-ζ (SV) one was used for oxygen, carbon, hydrogen and phosphorus. A polarizable continuum model (PCM) was introduced in the calculations with the parameters corresponding to the acetonitrile to reduce the delocalization error typical of DFT methods, mainly in charged systems with close molecular groups and opposite charges.[11] The magnetic coupling constants were estimated from the broken-symmetry approach according to Ruiz *et al*.[12] Thirteen different J_i magnetic couplings could describe the magnetic topology of the Ni_{12} entities present in **{Ni$_{12}$W$_{30}$}** and **{Ni$_{12}$W$_{27}$}**. However, given the absence of symmetry between the Ni_4 units, a total of 39 magnetic couplings have to be considered (J_{ia}, J_{ib}, and J_{ic}). The magnetic coupling pattern for **{Ni$_{12}$W$_{30}$}**

and **{$Ni_{12}W_{27}$}** is outlined in **Figure S32**. Although the structural differences between these units are not very significant, J_{ia-c} may be sufficiently different since the magnitude and nature of some of these interactions strongly depend on the Ni-O-Ni angle (*α*, **Figure S35**). To evaluate the 39 magnetic coupling constants, the calculation of at least 40 different spin configurations, one of reference and the remaining ones forming a linearly independent set of equations depending on J_i, is required. Due to the large size of the complete molecular model, only 31 spin configurations were calculated, and only 13 different J_i constants were assumed, that is, $J_{ia} = J_{ib} = J_{ic}$. Difficulties in the correct description of tungsten atomic orbitals and weak interactions between second neighbors considering that the pathways between two adjacent neighbors are partially made up of a single atom mainly contribute to the standard deviations given for the magnetic coupling constants thereby providing information on the approximation's accuracy. The coexistence of tungsten atoms forces the use of an atomic basis set different from the rest of atoms causing a marked slowdown in the convergence of the wavefunction. Hence, a model was built employing hydroxo groups to replace the tungsten centers while maintaining the positions of the oxygen atoms and placing the hydrogen atom ($d_{O–H}$: 0.98 Å) in the direction of the O–W bond, which allowed verifying the validity of the approximation that reduces the number of magnetic couplings to only thirteen.

Two approaches were subsequently applied:

1) 51 spin configurations were calculated in the simplified Ni_{12} model, applying one configuration as reference (high-spin, S = 12) and the remaining ones with their relative energies being expressed as a function of the J_i constants.

2) In both **{$Ni_{12}W_{30}$}** and **{$Ni_{12}W_{27}$}**, all Ni^{II} except the two Ni^{II} centers involved in the corresponding calculated coupling were substituted by diamagnetic Zn^{II} ions. This procedure was performed to simplify the complex magnetic structure endowed by the presence of multiple Ni^{II} cations to build a **Ni_2Zn_{10}** model for each magnetic coupling without affecting the region involved in the magnetic interaction, considering that the charge of the system remains unchanged.

In **{$Ni_{12}W_{27}$}**, the PO_4 groups appear in both deprotonated (**{PO_4}**$^{3-}$) and monoprotonated (**{HPO_4}**$^{2-}$) forms. Furthermore, the phosphate group encapsulated in the $[PW_9O_{34}]^{9-}$ lacunary ligands coordinates in a different way to three Ni^{II} ions of a Ni_4 entity, sharing a single oxygen atom in one of them (μ_3-OPO_3 or 3.3000-PO_4) and three of them in the remaining two Ni_4 units (3.1110-PO_4). This lower symmetry of the Ni_{12} entity compared to that in **{$Ni_{12}W_{30}$}** complicates the study, which was carried out only on the simplified molecular model. Considering a notation that indicates only the spin reversal, the 50 calculated configurations are: twelve S = 10 configurations ({1}, {2}, {3}, {4}, {5}, {6}, {7}, {8}, {9}, {10}, {11}, and {12}); twenty-two S = 8 configurations ({1,2}, {1,3}, {1,4}, {2,3}, {2,4}, {3,4}, {5,6}, {5,7}, {5,8}, {6,7}, {6,8}, {7,8}, {9,10}, {9,11}, {9,12}, {10,11}, {10,12}, {11,12}, {1,5}, {1,9}, {5,9}, and {1,7}); three S = 6 configurations ({1,2,3}, {5,6,7}, and {9,10,11}); three S = 4 configurations ({1,3,5,7}, {1,3,9,11}, and {5,7,9,11}); and ten S = 0 configurations ({1,3,5,7,9,11}, {5,6,7,8,10,12}, {5,6,7,8,11,12}, {5,6,7,9,10,11}, {5,6,7,9,10,12}, {3,6,8,9,11,12}, {3,6,8,10,11,12}, {3,6,9,10,11,12}, {3,7,8,9,10,11}, {3,7,8,9,10,12}). Complete active space (CAS) calculations were performed on $NiZn_{11}$ molecular models aiming at evaluating the axial (D) and rhombic (E) *zfs* parameters. These models were built from the original Ni_{12} entity of **{$Ni_{12}W_{30}$}** replacing the Ni^{II} by Zn^{II} ions except for the one Ni^{II} metal center of interest. The calculations were carried out with version 4.0 of the ORCA program[13] using the TZVP basis set proposed by Ahlrichs and the auxiliary TZV/C Coulomb fitting basis sets.[14] The spin-orbit coupling contributions to *zfs* from 10 triplet and 5 singlet excited states generated from an active space with eight electrons in five *d*-orbitals were included from an effective Hamiltonian. The *g*-tensors were calculated using Multireference Configuration Interaction (MRCI) wave functions with a first-order perturbation theory on the SOC matrix.[15]

Magnetic Studies: Variable-temperature (2–300 K) direct current (dc) magnetic susceptibility measurements under applied magnetic fields of 0.5 T (above 30 K) and 0.025 T (below 30 K) and variable-field (0–8 T) magnetization measurements at 2.0 K on powdered crystalline samples were carried out using Quantum Design Superconducting Quantum Interference Device (SQUID) magnetometer and Physical Property Measurement System (PPMS). The samples were embedded in *n*-eicosane to prevent any crystal reorientation. Variable-temperature (2–6 K) and variable-field (0–0.75 T) alternating current (*ac*) magnetic susceptibility measurements under ± 5.0 Oe oscillating field at frequencies in the range 1–10 kHz were performed with a Quantum Design PPMS. The magnetic susceptibility data were corrected for the diamagnetism of the constituent atoms and the sample holder.

High-Frequency/High-Field Electron Paramagnetic Resonance: HFEPR spectra of powdered crystalline samples of **{$Ni_{12}W_{30}$}** and **{$Ni_{12}W_{27}$}** at temperatures ranging from ca. 5 to 280 K were recorded on a home-built spectrometer at the Electron Magnetic Resonance facility of National High Magnetic Field Laboratory, Tallahassee, Florida. The setup of this instrument has been described in detail previously.[16] The instrument is a transmission type device in which microwaves are propagated in cylindrical lightpipes. The microwaves are generated by a phase-locked Virginia Diodes source, generating a frequency of 13 ± 1 GHz and producing its harmonics of which the 2nd, 4th, 6th, 8th, 16th, 24th, and 32nd are available. A superconducting magnet (Oxford Instruments) capable of reaching a field of 17 T was employed.

Photocatalytic H_2 evolution: The visible-light-driven hydrogen evolution experiments were carried using a 5 mL batch reactor equipped with a monochromatic LED light source (445±13 nm, power 2.5 mW/cm^2, incident light intensity 5 mW, Thorlabs SOLIS). The reactor volume was filled with a 2 mL solution mixture of 11:33:4 CH_3CN/DMF/H_2O containing $[Ir(ppy)_2(dtbbpy)]^+$ (dtbbpy = 4,4′-Di-*tert.*-butyl-2,2′-dipyridyl, ppy = 2-Phenylpyridine) as photosensitizer (0.2 mM), triethanolamine (TEOA) as proton donor (0.25 M), and the corresponding photocatalyst **{Ni_4W_{18}}**, **{$Ni_{12}W_{27}$}**, or **{$Ni_{12}W_{30}$}** (2-20 µM). Exposure to ambient light was minimized during the solution mixture preparation and transfer to the reactor. The reaction volume was purged with Ar for 10 min to ensure the removal of headspace and dissolved oxygen prior to reaction start. The temperature of the reactor was maintained at 15 °C with a water-cooling system. The reaction mixture was stirred at 1150 rpm. The H_2 produced was monitored by sampling the reactor headspace (100 µL) and analyzing its composition via gas chromatography (Shimadzu GC 2030) equipped with a barrier ionization discharge detector and a Micropacked-ST column using helium as a carrier gas. Injections were done with an interval of 10 minutes. The calibration was done using a range of H_2 in argon gas mixtures. The H_2 concentrations in ppm (derived from the chromatograms) were converted to µmol and turnover numbers (TONs – expressed per catalyst cluster/species) based on reactor parameters and the ideal gas equation. Initial turnover frequencies (TOFs) were calculated after 10 minutes of illumination (in most of the cases a close to linear H_2 evolution trend within the first 20 minutes of HER was observed). The calculation of the quantum yields (QYs, better known as internal quantum efficiency IQE values) considered the ratio between the number of H_2 molecules produced and the number of photons absorbed by the reaction solution. The latter was extracted using a power meter PM100D (Thorlabs) by measuring photon flux at the reactor position.

X-ray fluorescence: Chemical analysis with total-reflection X-ray fluorescence (TXRF) was performed using an Atomika 8030C X-ray fluorescence analyzer to analyze the supernatant obtained upon precipitation and subsequent centrifugation of **{$Ni_{12}W_{27}$}** or **{$Ni_{12}W_{30}$}** (for experimental details see section 13.4). This spectrometer operates with a total reflection geometry using an energy-dispersive Si(Li) detector, and the measurements were done with monochromatized Mo-*Kα* excitation mode (20.2 keV) at 50 kV and 47 mA, for 100 s live time. All reflectors were washed thoroughly and measured to account for true blanks. 995 µL of each sample was pipetted into an Eppendorf tube and 5 µL of a Cr internal standard solution (c = 1000 ppm = 1000 mg/L) were added into the tube (total volume = 1000 µL) resulting in a final Cr concentration of 5 ppm. The Eppendorf tubes were vortexed for at least 1 min and 5 µL of the sample solutions containing the internal standard were pipetted in the middle of the reflector followed by subsequent addition of 45 µL Cr standard solution (c = 1000 ppm) giving a total volume of V = 1040 µL and a concentration of 50 ppm internal standard. After drying for 5 min on a hot plate and cooling, the dried samples were measured. The results are summarized in **Table S15**, section 13.4. entitled Total X-ray fluorescence (TXRF) experiments.

Photoluminescence (PL) spectroscopy: PL steady state measurements of 0.2 mM $[Ir(ppy)_2(dtbbpy)]^+$ solutions (with and without quenchers) were performed using a Picoquant FluoTime 300 spectrophotometer with a Xe arc lamp (300 W power) as excitation source coupled with a double-grating monochromator. The detection system was composed of a PMA Hybrid 07 detector along with a high-resolution double monochromator. The excitation wavelength utilized for all steady state measurements was 445 nm (2.79 eV photon energy). The concentration of the $[Ir(ppy)_2(dtbbpy)]^+$ solution was set to be in the range to exclude any inner filter effects. Time-resolved PL spectra were obtained using a laser wavelength of 377 nm, keeping the detection wavelength at 590 nm for all measured solutions. The collected data was fitted using the EasyTau2 software.[17]

2. Synthesis Procedure

When this paper was under preparation, a crystal structure identical with the anion of **{$Ni_{12}W_{30}$}** has been reported.[18] Note that for the synthesis of **{$Ni_{12}W_{30}$}** different routes have been used by Lian *et al.* and our group. The structures reported by Lian *et al.* were synthesized starting from the trilacunary Na_9[*A*-PW_9O_{34}] · $7H_2O$ building block in a Teflon bomb in the presence of $ZrOCl_2$ $8H_2O$ and Na_3PO_4 as an additional phosphate source. The synthesis protocol reported in this work applies the **{P_2W_{19}}** precursor which allows for the use of comparably mild bench conditions (80°C for 10 min) without requiring additional phosphate or $ZrOCl_2$ (**Scheme S1**). In contrast to the procedure of Lian *et al.* which leads to the mixed dimethylammonium (DMA) – cesium salt of **{$Ni_{12}W_{30}$}**, our procedure offers the potassium - sodium salt further allowing subsequent ion exchange according to an established literature procedure[1a] to yield the tetrabutylammonium (TBA) salt that is ultimately subjected towards homogeneous HER studies.

The syntheses of **{$Ni_{12}W_{27}$}** and **{$Ni_{12}W_{30}$}** start with the preparation of the literature known phosphotungstate building block $K_{14}[P_2W_{19}O_{69}(H_2O)]$ • $24H_2O$[1b] **{P_2W_{19}}** which was chosen based on its rich solution chemistry[19], propensity to dissociate into *A*- and *B*- isomers of {PW_9}-units[20] and additional tungstate structural fragments, as well as its affinity towards Ni^{II} electrophiles.[21] To an aqueous solution of **{P_2W_{19}}**, 3 eq. of $NiCl_2$ were added, and the pH of the resulting light green reaction mixture (pH = 6.8) was adjusted to 5.5 using HCl [1 M] to allow the use of increased PO_4^{3-} or CO_3^{2-} amounts for the subsequent basification (pH = 9.1) and templated formation of **{$Ni_{12}W_{27}$}** (using PO_4^{3-}) or **{$Ni_{12}W_{30}$}** (using CO_3^{2-}) upon heat activation (**Scheme S1**). Note that control experiments lacking the acidification step *via* HCl yielded the same products **{$Ni_{12}W_{27}$}** (using PO_4^{3-}) or **{$Ni_{12}W_{30}$}** (using CO_3^{2-}), however, in significantly lower yields (Yield: 4% based on W for **{$Ni_{12}W_{30}$}** and 7% for **{$Ni_{12}W_{27}$}**) as compared to the optimized reaction system featuring pre-acidified solutions (Yield: 15% based on W for **{$Ni_{12}W_{30}$}** and 12% for **{$Ni_{12}W_{27}$}**).

Scheme S1. Schematic representation of the template dependent synthesis of **{$Ni_{12}W_{27}$}** and **{$Ni_{12}W_{30}$}** starting from a solution containing the lacunary **{P_2W_{19}}** phosphotungstate precursor and $NiCl_2$ in a 1:3 ratio (pH =5.5 via [1 M] HCl). Basification of the reaction mixture to pH = 9.1 (via Na_3PO_4 or Na_2CO_3) and subsequent heat activation results in **{$Ni_{12}W_{27}$}** (using Na_3PO_4) or **{$Ni_{12}W_{30}$}** (using Na_2CO_3), respectively. Turquoise and purple polyhedra for {WO_6} and {PO_4}, red spheres represent oxygen ions.

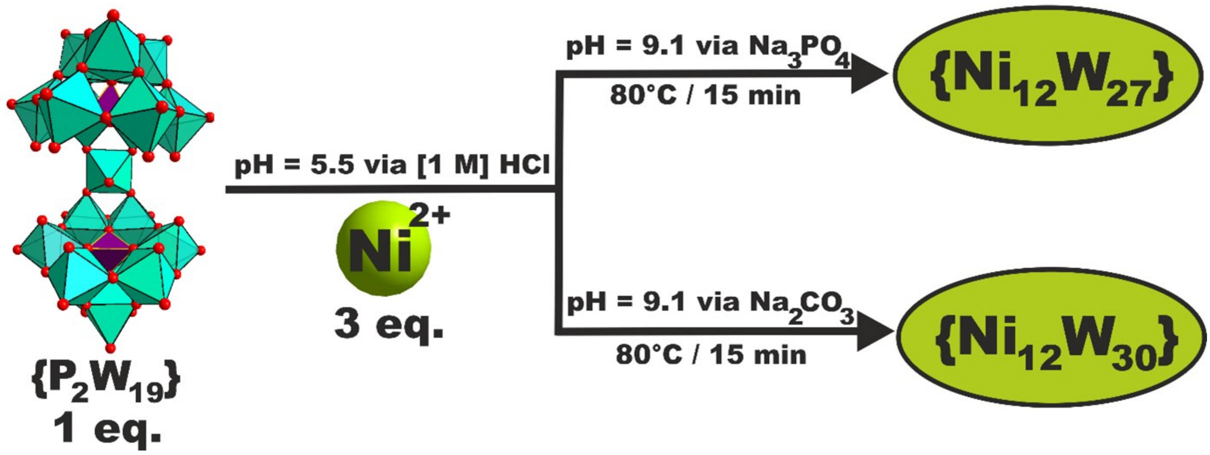

2.1. Synthesis of $K_{11}Na_{10}[Ni_{12}(OH)_9(WO_4)_3(PO_4)$(*B*-*α*-$PW_9O_{34})_3]$ • 98 H_2O ($K_{11}Na_{10}$-**{$Ni_{12}W_{30}$}**)

Solid $K_{14}[P_2W_{19}O_{69}(H_2O)]$ • $24H_2O$ (5.6 g, 1.0 mmol) synthesized according to the published procedure[1a] was dissolved in H_2O (100 mL) under heating the solution to 80 ºC. After the reaction mixture was cooled

down to room temperature, solid $NiCl_2 \cdot 6\ H_2O$ (740 mg, 3.1 mmol) was added. The pH value of the light green reaction mixture was adjusted to 5.5 with HCl [1 M] followed by addition of solid Na_2CO_3 until a pH of 9.1 was reached. Importantly, the HCl and Na_2CO_3 must be added stepwise with the next addition only when the pH value starts to rise or fall, respectively. The dark green solution was heated to 80°C for 15 min, cooled down to room temperature and left for crystallization at 20°C. Light-green, almost colorless rod-shaped crystals started to appear after ca 24 h and were filtered and air dried after four days. Yield: 1.6 g, 15% based on W. Elemental analysis calcd (found) for ($K_{11}Na_{10}Ni_{12}W_{30}H_{205}O_{225}P_4$): K 3.98 (5.83), Na 2.13 (4.60), Ni 6.52 (5.87), W 51.02 (55.63), P 1.15 (2.28).

2.2. Ion-exchange procedure – preparation of $(C_{16}H_{36}N)_{13}Na_8[Ni_{12}(OH)_9(WO_4)_3(PO_4)(B\text{-}\alpha\text{-}PW_9O_{34})_3] \cdot 13\ H_2O$ (TBA$_{13}$Na$_8$-**{Ni$_{12}$W$_{30}$}**)

1.0 g of $K_{11}Na_{10}$-**{Ni$_{12}$W$_{30}$}** (0.1 mmol) in 30 mL H_2O were added to a solution of 4.8 g tetrabutyl ammonium bromide (15 mmol) in 20 mL 0.25 M sodium acetate buffer (pH 4.8) resulting in immediate precipitation of a light green solid. The dried solid was then re-dissolved in CH_3CN (5 mL) and the mixture centrifuged to remove any undissolved precipitate. The final products were obtained in high purity by adding anhydrous diethyl ether (40 mL) to the green CH_3CN solution. Yield: 1.1 g, 91 % based on W. Elemental analysis calcd (found) for ($C_{208}H_{503}N_{13}O_{140}P_4W_{30}Ni_{12}Na_8$): C 20.90 (22.12), H 4.24 (4.52), N 1.52 (1.62), O 18.74 (18.88).

2.3. Synthesis of $K_{14}Na_7[Ni_{12}(OH)_9(HPO_4)_3(PO_4)(B\text{-}\alpha\text{-}PW_9O_{34})(A\text{-}\alpha\text{-}PW_9O_{34})_2] \cdot 44\ H_2O$ (K$_{14}$Na$_7$-**{Ni$_{12}$W$_{27}$}**)

In a 400 ml beaker, solid $K_{14}[P_2W_{19}O_{69}(H_2O)] \cdot 24H_2O$ (5.6 g, 1.0 mmol) synthesized according to the published procedure[1a] was dissolved in H_2O (100 mL) under heating the solution to 80 ºC. After the reaction mixture was cooled down to room temperature, solid $NiCl_2 \cdot 6\ H_2O$ (740 mg, 3.1 mmol) was added. The pH value of the light green reaction mixture was adjusted to 5.5 with HCl [1 M] followed by addition of solid Na_3PO_4 until a pH of 9.0-10.0 was reached. Importantly, the HCl and Na_3PO_4 must be added stepwise with the next addition only when the pH value starts to rise or fall, respectively. The yellow solution was heated to 80°C for 15 min, cooled down to room temperature, centrifuged to remove any undissolved precipitates and left for crystallization at 20°C. Yellow plate shaped crystals started to appear after ca 24 h and were filtered after three days. Yield: 1.3 g, 12% based on W. Elemental analysis calcd (found) for ($K_{14}Na_7Ni_{12}W_{27}H_{100}O_{171}P_7$): K 5.80 (7.32), Na.1.71 (2.76), Ni 7.47 (6.16), W 52.64 (57.68), P 2.30 (2.73).

2.4. Ion-exchange procedure – preparation of $(C_{16}H_{36}N)_{13}Na_8[Ni_{12}(OH)_9(HPO_4)_3(PO_4)(B\text{-}\alpha\text{-}PW_9O_{34})(A\text{-}\alpha\text{-}PW_9O_{34})_2] \cdot 11\ H_2O$ (TBA$_{13}$Na$_8$-**{Ni$_{12}$W$_{27}$}**)

1.0 g of $K_{14}Na_7$-**{Ni$_{12}$W$_{27}$}** (0.1 mmol) in 30 mL H_2O were added to a solution of 4.8 g tetrabutyl ammonium bromide (15 mmol) in 20 mL 0.25 M sodium acetate buffer (pH 4.8) resulting in immediate precipitation of a yellowish solid. The dried solid was then re-dissolved in CH_3CN (5 mL) and the mixture centrifuged to remove any undissolved precipitate. The final products were obtained in high purity by adding anhydrous diethyl ether (40 mL) to the CH_3CN solution. Yield: 940 mg, 82% based on W. Elemental analysis calcd (found) for ($C_{208}H_{502}N_{13}O_{138}P_7W_{27}Ni_{12}Na_8$): C 21.79 (18.68), H 4.41 (3.85), N 1.59 (1.37), O 19.26 (19.30).

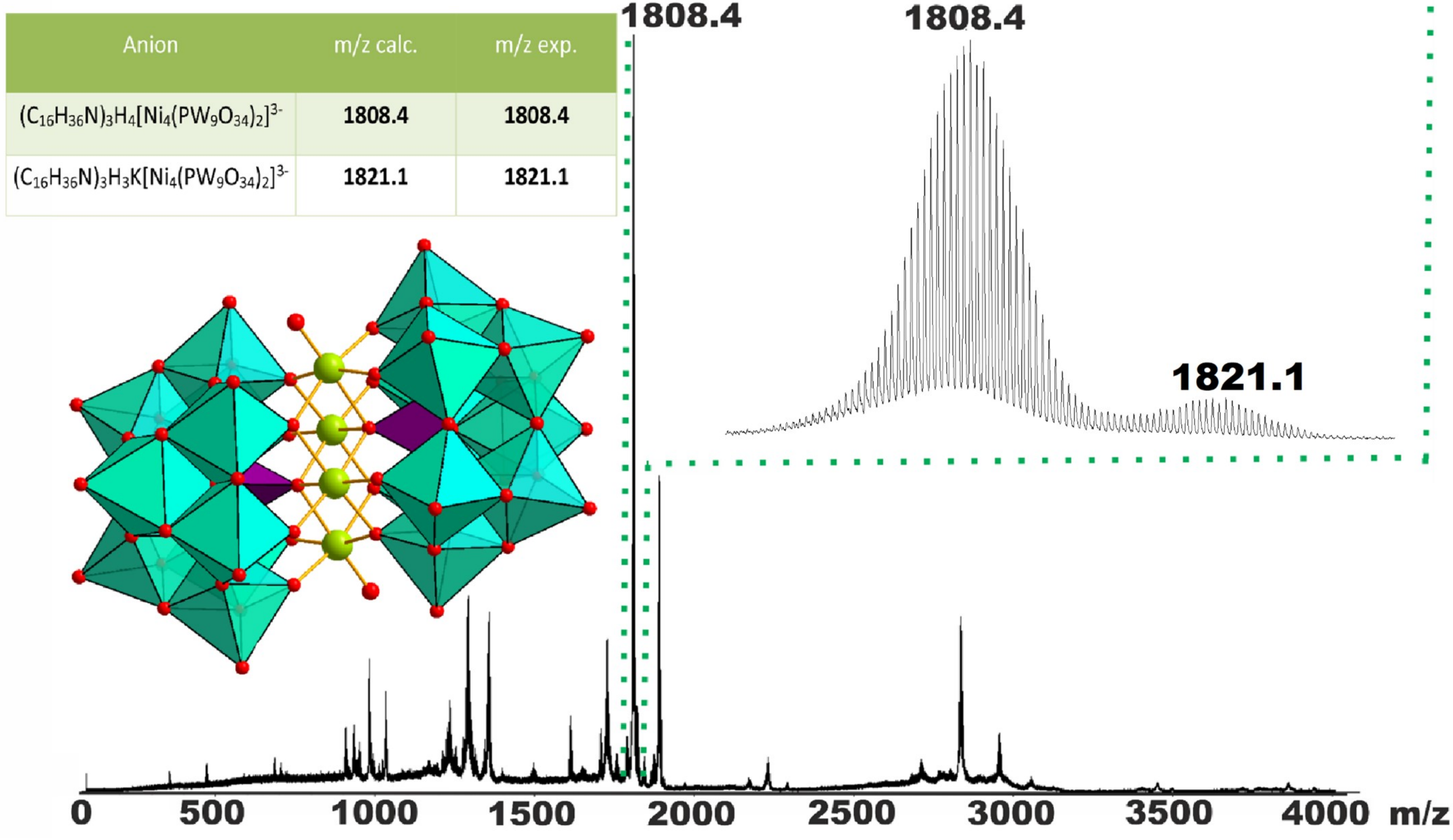

Anion	m/z calc.	m/z exp.
$(C_{16}H_{36}N)_3H_4[Ni_4(PW_9O_{34})_2]^{3-}$	**1808.4**	**1808.4**
$(C_{16}H_{36}N)_3H_3K[Ni_4(PW_9O_{34})_2]^{3-}$	**1821.1**	**1821.1**

Figure S1. Negative ion-mode ESI-MS spectrum of **{Ni_4W_{18}}** in $H_2O/CH_3CN/MeOH$ mixture.

Table S1. Survey of existing crystal structures of nickel – cubane incorporating POTs according to Scifinder and the ICSD database (**August**, 2022). enMe = 1,2-diaminopropane, OAc = acetate, dien = diethylenetriamine.

POT	Investigated properties	Number of Ni centers	Number/Types of cubanes	Ref.
**$[H_2PW_9Ni_4O_{34}(OH)_3(H_2O)_6]^{2-}$**	Magnetism (ferromagnetic interactions between Ni-centers), *S* = 4	4	1 $\{Ni^{II}_4O_4\}$ cubane	22
**$\{[SiW_9O_{34}Ni_4(OH)_3(OAc)_3]\}_2^{15-}$**	Magnetism (ferromagnetic & antiferromagnetic interactions between Ni-centers), *S* = 2	8	2 $\{Ni^{II}_4O_3\}$ - deficient cubane (tetrahedron)	23
**$[Na\{(A\text{-}\alpha\text{-}SiW_9O_{34})Ni_4(OAc)_3(OH)_2(N_3)\}_2]^{15-}$**	Electrochemical properties	8	2 $\{Ni^{II}_4O_3\}$ - deficient cubane (tetrahedron)	23
**$[(A\text{-}\alpha\text{-}SiW_9O_{34})_2Ni_9(OH)_6(H_2O)_6(CO_3)_3]^{14-}$**	Magnetism (antiferromagnetic interactions between Ni-centers), *S* = 4	9	2 $\{Ni^{II}_4O_3\}$ - deficient cubane (tetrahedron)	23
**$[(SiW_8O_{31})_2Ni_7(H_2O)_4(OH)_6]^{12-}$**	Electrochemical properties	7	1 double-cubane $\{Ni^{II}_3O_4\}Ni^{II}\{Ni^{II}_3O_4\}$ = $\{Ni^{II}_{3.5}O_4\}_2$	24
**$[Ni_{12}(OH)_9WO_4(W_7O_{26}(OH))(PW_9O_{34})_3]^{25-}$**	Magnetism (dominating intramolecular ferromagnetic interactions between Ni-centers competing with weak antiferromagnetic interactions), *S* not reported	12	3 $\{Ni^{II}_4O_4\}$ cubanes	25
**$[Ni_{12}(OH)_9(CO_3)_3(PO_4)(SiW_9O_{34})_3]^{24-}$**	WOC	12	1 $\{Ni^{II}_3O_4\}$ quasi-cubane, 3 $\{Ni^{II}_4O_3\}$ - deficient cubanes (tetrahedra)	26
**$[Ni_{13}(H_2O)_3(OH)_9(PO_4)_4(SiW_9O_{34})_3]^{25-}$**	WOC	13	1 $\{Ni^{II}_4O_4\}$ cubane, 3 $\{Ni^{II}_4O_3\}$ - deficient cubanes (tetrahedra)	26
**$[Ni_{25}(H_2O)_2(OH)_{18}(CO_3)_2(PO_4)_6(SiW_9O_{34})_6]^{50-}$**	WOC	25	2 $\{Ni^{II}_3O_4\}$ quasi-cubanes, 6 $\{Ni^{II}_4O_3\}$ - deficient cubanes (tetrahedra)	26

**$[\{Ni_4(OH)_3AsO_4\}_4(\textit{B}\text{-}\alpha\text{-}PW_9O_{34})_4]^{28-}$**	Magnetism (dominating ferromagnetic interactions), *S* not reported/WRC	16	4 $\{Ni^{II}_4O_4\}$ cubanes	27
**$[Ni_3(H_2O)_3(PW_{10}O_{39})H_2O]^{7-}$**	Magnetism (ferromagnetic interactions between Ni-centers), *S* = 3	3	1 $\{W^{VI}Ni^{II}_3O_4\}$ quasi-cubane	28
**$[Ni_6(\mu_3\text{-}OH)_3(H_2O)_6(enMe)_3(\textit{B}\text{-}\alpha\text{-}SiW_9O_{34})]^{1-}$**	-	6	3 $\{Ni^{II}_3O_4\}$ quasi-cubanes	29
**$[Ni_6(\mu_3\text{-}OH)_3(H_2O)_4(enMe)_3(OAc)(\textit{B}\text{-}\alpha\text{-}PW_9O_{34})]^{1-}$**	Magnetism (ferromagnetic interactions between Ni-centers), *S* = 6	6	3 $\{Ni^{II}_3O_4\}$ quasi-cubanes	29
**$[Ni_6(\mu_3\text{-}OH)_3(H_2O)_2(dien)_3(\textit{B}\text{-}\alpha\text{-}PW_9O_{34})]$**	Magnetism (ferromagnetic interactions between Ni-centers), *S* = 6	6	3 $\{Ni^{II}_3O_4\}$ quasi-cubanes	29
**$[H_6Ni_{20}P_4W_{34}(OH)_4O_{136}(enMe)_8(H_2O)_6]^{6-}$**	Magnetism (dominating ferromagnetic interactions between Ni-centers), *S* not reported	20	3 $\{Ni^{II}_3O_4\}$ quasi-cubanes	30
**$[\{Ni_4(OH)_3(PO_4)\}_4(\textit{A}\text{-}PW_9O_{34})_4]^{28-}$**	WRC	16	4 $\{Ni^{II}_4O_4\}$ cubanes	27, 31
**$[\{Ni_4(OH)_3(PO_4)\}_4(\textit{A}\text{-}PW_9O_{34})_2(\textit{B}\text{-}PW_9O_{34})_2]^{28-}$**	WRC	16	4 $\{Ni^{II}_4O_4\}$ cubanes	31
**$[\{Ni_4(OH)_3(VO_4)\}_4(\textit{B}\text{-}PW_9O_{34})_4]^{28-}$**	WRC	16	4 $\{Ni^{II}_4O_4\}$ cubanes	31
**$\{Ni_{12}(OH)_9(PO_4)_4(\textit{A}\text{-}\alpha\text{-}SiW_9O_{34})[W_4O_{10}(OH)(PO_2(OH)_2)_2(\textit{A}\text{-}\alpha\text{-}SiW_9O_{34})_2]\}^{26-}$**	CO_2 reduction/Magnetism (competing ferro – & antiferromagnetic interactions, *S* not reported)	12	3 $\{Ni^{II}_4O_3\}$ - deficient cubanes (tetrahedra)	32
**$[Ni_3(OH)_3(H_2O)_3P_2W_{16}O_{59}]^{9-}$**	WRC	3	1 $\{W^{VI}Ni^{II}_3O_4\}$ quasi-cubane	33
**$[Ni_{36}(OH)_{18}(H_2O)_{36}(SiW_9O_{34})_6]^{6-}$**	Magnetism (F interactions/unit + AF between units)	36	$\{Ni^{II}_3O_4\}$ quasi-cubane	34
**$[Ni_{12}(OH)_9(WO_4)_3(PO_4)(\textit{B}\text{-}\alpha\text{-}PW_9O_{34})_3]^{21-}$**	Catalytic Knoevenagel condensation reaction	12	3 $\{Ni^{II}_4O_4\}$ cubanes, 1 $\{Ni^{II}_3O_4\}$ quasi - cubane	18
**$[Ni_{12}(OH)_9(WO_4)_3(PO_4)(\textit{B}\text{-}\alpha\text{-}PW_9O_{34})_3]^{21-}$**	Magnetism (Single molecule magnet behavior, *S* = 0)/WRC	12	3 $\{Ni^{II}_4O_4\}$ cubanes, 1 $\{Ni^{II}_3O_4\}$ quasi - cubane	**This work**
**$[Ni_{12}(OH)_9(HPO_4)_3(PO_4)(\textit{B}\text{-}\alpha\text{-}PW_9O_{34})(\textit{A}\text{-}\alpha\text{-}PW_9O_{34})_2]^{21-}$**	Magnetism (Single molecule magnet behavior, *S* = 6)/WRC	12	3 $\{Ni^{II}_4O_3\}$ - deficient cubanes (tetrahedra), 1 $\{Ni^{II}_3O_4\}$ quasi - cubane	**This work**

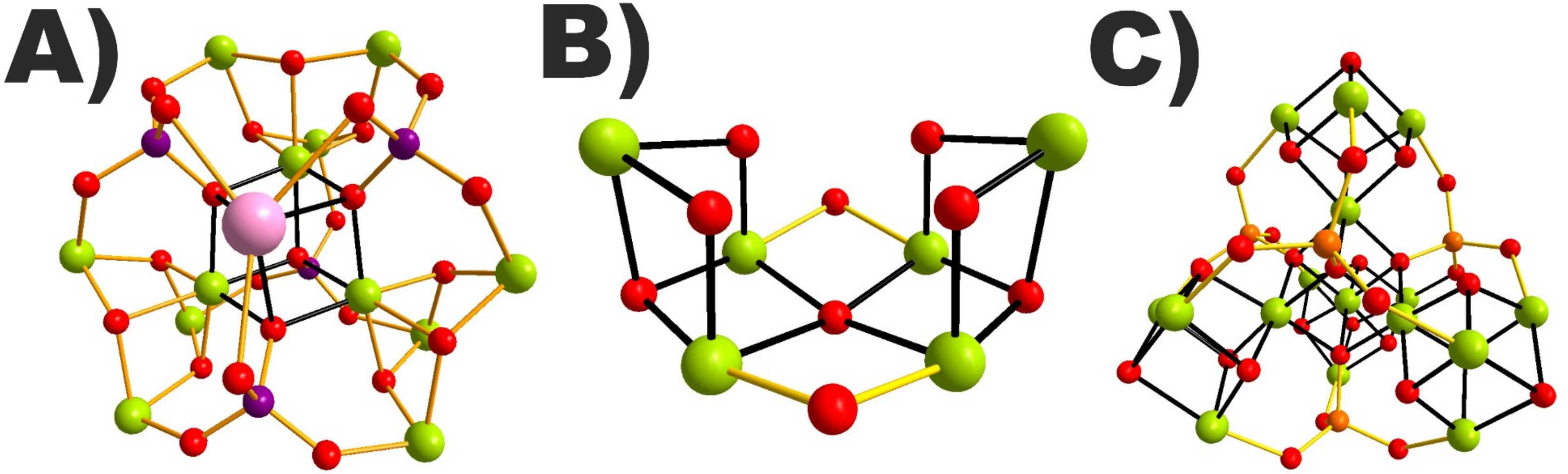

Figure S2. Ball and stick representation of the Ni^{II} metal-oxo cores enclosing **A)** a **$\{Ni^{II}_3O_4\}$** quasi-cubane in **$\{Ni_{12}Si_3W_{31}\}$**, **B)** a double **μ_4-O$\{Ni^{II}_3O_4\}_2$** quasi-cubane in **$\{Ni_6Si_2W_{20}\}$** and **C)** a penta-cubane **$\{Ni^{II}_4O_4\}_5$** in **$\{Ni_{16}W_{36}\}$**. Bonds belonging to cubane motifs are highlighted in black. Color code: Ni^{II}, lime; P^V, purple; O, red; As^V, orange; K, pink.

Table S2. Survey of photocatalytic POT-WRCs according to Scifinder (works citing Zhang, Z.; Lin, Q.; Zheng, S.-T.; Bu, X.; Feng, P. A Novel Sandwich-Type Polyoxometalate Compound with Visible-Light Photocatalytic H_2 Evolution Activity. *Chem. Commun*. **2011**, *47* (13), 3918–3920) and the *web of knowledge* database (using the key words "homogeneous hydrogen evolution polyoxometalate" in the search engine) (**August, 2022**). Note that various parameters such as the shape of the reaction vessel, light intensity, stirring rate as well as the ratio of gaseous head space to total volume render a direct comparison of the WRC performance difficult.[35] DMF = N, N', -dimethylformamide; bpy = 2, 2' – bipyridine; dtbbpy = 4,4′-bis(1,1-dimethylethyl)-2,2′-bipyridine-N1,N1′; ppy = 2-(2-pyridinyl-N)phenyl-C; TEA = triethylamine; Fl = fluorescein; dien = diethylenetriamine; ale = alendronate, aleH = alendronic acid, EY^{2-} = Eosin-Y, PVA = polyvinyl alcohol, pdc = 3,4-pyridinedicarboxylate, TEOA = triethanolamine.

POT	TON	TOF, in × 10^{-3} s^{-1} (normalized for comparability)	TOF as reported	Quantum yield (QY), %	Representative Reaction conditions	Ref.
**$[Ni(H_2O)GeW_{11}O_{39}]^{6-}$**	36.8	9	9 × 10^{-3} s^{-1}	-	High power LED (λ = 470 nm), 1.0 mM $[Ru(bpy)_3]Cl_2$, 0.12 M ascorbate buffer, pH 4	36
**$[Ni(H_2O)PW_{11}O_{39}]^{5-}$**	10.9	9.7	9.7 × 10^{-3} s^{-1}	-	High power LED (λ = 470 nm), 1.0 mM $[Ru(bpy)_3]Cl_2$, 0.12 M ascorbate buffer, pH 4	36
**$[Ni(H_2O)SiW_{11}O_{39}]^{6-}$**	inactive	-	-	-	High power LED (λ = 470 nm), 1.0 mM $[Ru(bpy)_3]Cl_2$, 0.12 M ascorbate buffer, pH 4	36
**$[Mn_4(H_2O)_2(VW_9O_{34})_2]^{10-}$**	42 (5.5 h)	2.12	-	-	LED light (20 mW, 455 nm, beam diameter 0.4 cm), 0.67 mM $[Ru(bpy)_3]Cl_2$, TEOA (0.25 M), 2 mL DMF/H_2O (1.86/1)	37

**[*α*-Sn$_4$(SiW$_9$O$_{34}$)$_2$]$^{12–}$**	1.4 (56 h)	0.007	0.025 h^{-1}	0.025	300 W Xe lamp (400 nm cut-off filter), 0.5 g H$_2$PtCl$_6$ co-catalyst, 270 mL MeOH (20 vol%) solution	38
**[{Ni$_4$(OH)$_3$AsO$_4$}$_4$(*B*-*α*-PW$_9$O$_{34}$)$_4$]$^{28–}$**	580 (5 h)	32	116 h^{-1}	4.07	LED light (20 mW, 455 nm, beam diameter 0.4 cm), 0.2 mM [Ir(ppy)$_2$(dtbbpy)][PF$_6$], TEOA (0.25 M), H$_2$O (1.4 M), CH$_3$CN/DMF (1:3 v/v, 2 mL)	27
**{Ni$_{12}$(OH)$_9$(PO$_4$)$_4$(*A*-*α*-SiW$_9$O$_{34}$)[W$_4$O$_{10}$(OH)(PO$_2$(OH)$_2$)$_2$(*A*-*α*-SiW$_9$O$_{34}$)$_2$]}$^{26-}$**	23.71 (1 h)	6.6	23.71 h^{-1}	-	[Ru(bpy)$_3$]Cl$_2$·6H$_2$O (0.01 mmol) TEOA (1 mL), H$_2$O (1 mL) and acetonitrile (MeCN, 4 mL). The reaction setup was alternately vacuum degassed and purged with CO$_2$ three times, after which high-purity CO$_2$ was purged again for 30 min, λ ≥ 420 nm, 30°C, 1 h.	32
**[Ni$_6$(OH)(BO$_3$)$_2$(dien)$_2$(*B*-*α*-SiW$_{10}$O$_{37}$)$_2$]$^{30-}$**	83.03 (1 h)	23.1	83.03 h^{-1}	-	[Ru(bpy)$_3$]Cl$_2$·6H$_2$O (0.01 mmol) TEOA (1 mL), H$_2$O (1 mL) and acetonitrile (MeCN, 4 mL). The reaction setup was alternately	32

					vacuum degassed and purged with CO_2 three times, after which high-purity CO_2 was purged again for 30 min, λ ≥ 420 nm, 30°C, 1 h.	
**$[Ni_3(OH)_3(H_2O)_3P_2W_{16}O_{59}]^{9-}$**	160 (3 h)	14.8	53.3 h^{-1}	-	LED light (20 mW, 455 nm, beam diameter 0.4 cm), 0.2 mM $[Ir(ppy)_2(dtbbpy)][PF_6]$, TEOA (0.25 M), H_2O (1.4 M), CH_3CN/DMF (1:3 v/v, 2 mL), deaerated with argon.	33
**$[\{Ni_4(OH)_3(PO_4)\}_4(\textit{A}\text{-}PW_9O_{34})_4]^{28-}$**	578.8 (12 h)	27.9	100.5 h^{-1} (0.5 h)	-	100 W LED light (400–780 nm), 0.2 mM $[Ir(ppy)_2(dtbbpy)][PF_6]$, 2 mL TEOA, 11 mL CH_3CN, 33 mL DMF, and 4 mL H_2O	31, 27
**$[\{Ni_4(OH)_3(PO_4)\}_4(\textit{A}\text{-}PW_9O_{34})_2(\textit{B}\text{-}PW_9O_{34})_2]^{28-}$**	679.1 (12 h)	31.3	112.7 h^{-1} (0.5 h)	-	100 W LED light (400–780 nm), 0.2 mM $[Ir(ppy)_2(dtbbpy)][PF_6]$, 2 mL TEOA, 11 mL CH_3CN, 33 mL DMF, and 4 mL H_2O	31
**$[\{Ni_4(OH)_3(VO_4)\}_4(\textit{B}\text{-}PW_9O_{34})_4]^{28-}$**	931.1 (12 h)	51.5	185.5 h^{-1} (0.5	-	100 W LED light (400–780 nm), 0.2	31

			h)		mM $[Ir(ppy)_2(dtbbpy)][PF_6]$, 2 mL TEOA, 11 mL CH_3CN, 33 mL DMF, and 4 mL H_2O	
**$[Ni_4(H_2O)_2(TiW_9O_{34})_2]^{12-}$**	2000 (3h)	185.2	666.6 h^{-1}	0.5	room temperature; 300 W Xenon light, λ ≥ 400 nm; POT [10 μM]; Fl 4 mM; TEA 6%	39
**$[(\alpha\text{-}A\text{-}SiW_9O_{34})Ni_{14}(AleH)_5(Ale)_2(H_2O)_{11}(OH)_7]^{12-}$**	256 (4h)	27	2.7×10^{-2} s^{-1}	-	0.2 mM [Ir]-PS, 0.25 M TEOA, 0.1 M BNAH, 2 mL CH_3CN, catalyst conc.= 20 μM, Xenon arc lamp (λ= 415-800 nm, 280 mW), 20 μM TBA-$P_2W_{18}Ni_4$ (as reference)	40
**$[Co^{III}Co^{II}(H_2O)W_{11}O_{39}]^{7-}$**	100	25 (10 min)	0.025 s^{-1}	(29, when co-catalyzed by Pt indicating the POM's role as a charge mediator in this case)	40 μM POT-catalyst, 50 μM EY^{2-}, 5 wt% Pt as H_2PtCl_6 as co-catalyst, in 5% (v/v) TEOA/H_2O (pH = 7.0), irradiated via 3 W LED (λ > 420 nm)	41
**$[\{Co_3(B\text{-}\beta\text{-}SiW_9O_{33}(OH))(B\text{-}\beta\text{-}$**	8.55	-	-	-	300 W Xe lamp (λ =	42

**$SiW_8O_{29}(OH)_2)\}_2]^{22-}$**					200 to 1100 nm), 0.05 M POM, 0.38 mM colloidal TiO_2, PVA as electron donor, 0.5% Pt co-catalyst	
**$[Co_6(H_2O)_2(PW_9O_{34})_2(PW_6O_{26})]^{17-}$**	-	2.8	10 h^{-1}	-	300 W Xe lamp with a cut-off filter (λ ≥ 400 nm), EY, TEOA	43
**$[Cu_4(H_2O)_2(B\text{-}\alpha\text{-}PW_9O_{34})_2]^{10-}$.**	~745 (5h)	-	-	5.2 (for 20 μM)	0.2 mM $[Ir(ppy)_2(dtbbpy)]^+$, 0.25 M TEOA, 1.4 M H_2O and 4−20 μM catalyst	44
**$[Ni_4(H_2O)_2(SiW_{10}O_{38})_2]^{8-}$**	-	7.8	28 h^{-1}	-	Fluorescein, TEOA, a 300 W Xe lamp (λ = > 420 nm)	45
**$[(pdc)_2La(H_2O)_2SiW_{11}O_{39}]^{7-}$**	525 (7h)	90	324 h^{-1}	-	20 μM $[Ru(bpy)_3]^{2+}$, Ascorbic Acid/ H_2O:MeOH (9:1, v:v)	46
**$[(pdc)_2Pr(H_2O)_2SiW_{11}O_{39}]^{7-}$**	448 (7h)	71.7	258 h^{-1}	-	20 μM $[Ru(bpy)_3]^{2+}$, Ascorbic Acid/ H_2O:MeOH (9:1, v:v)	46
**$[(pdc)_2Dy(H_2O)_2SiW_{11}O_{39}]^{7-}$**	162 (7h)	18.1	65 h^{-1}	-	20 μM $[Ru(bpy)_3]^{2+}$, Ascorbic Acid/ H_2O:MeOH (9:1, v:v)	46

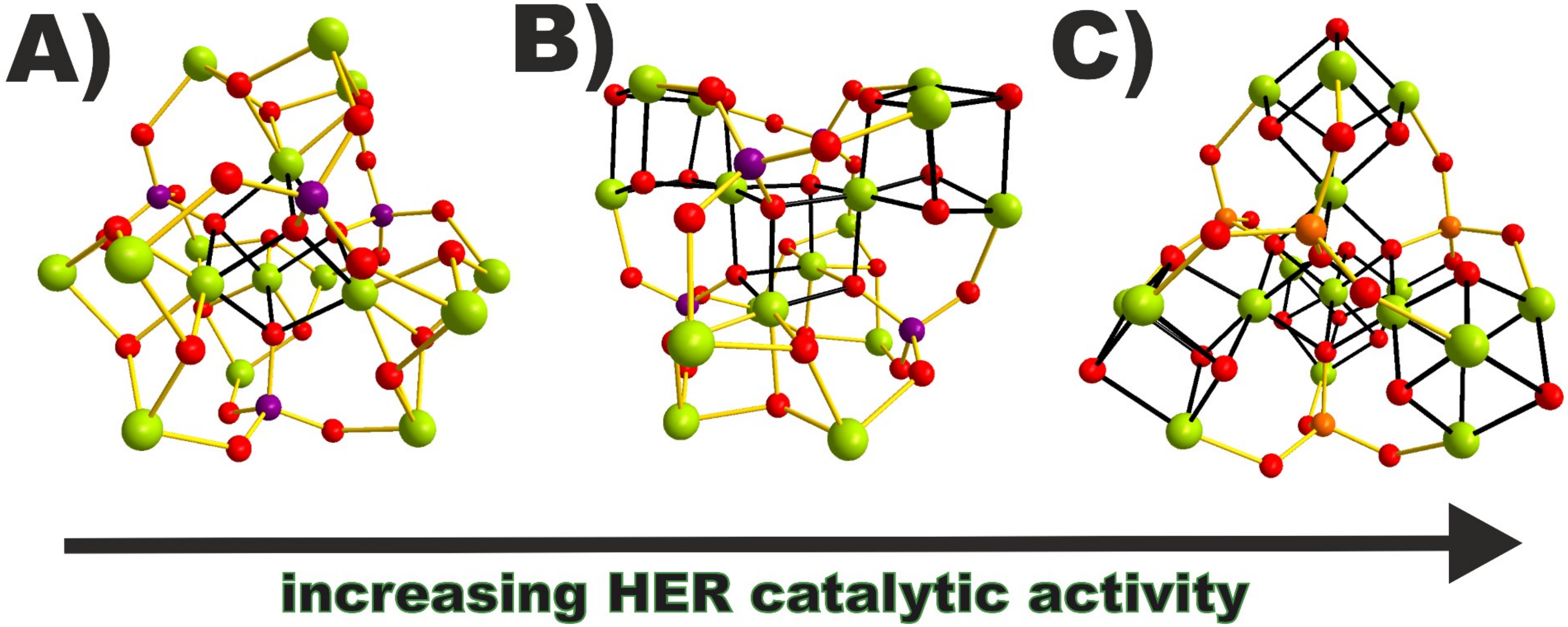

Figure S3. Ball and stick representation of the Ni^{II} metal-oxo cores in **A)** $[\{Ni_4(OH)_3(PO_4)\}_4(A\text{-}PW_9O_{34})_4]^{28-}$ (one cubane), **B)** $[\{Ni_4(OH)_3(PO_4)\}_4(A\text{-}PW_9O_{34})_2(B\text{-}PW_9O_{34})_2]^{28-}$ (three cubanes) and **C)** $[\{Ni_4(OH)_3(VO_4)\}_4(B\text{-}PW_9O_{34})_4]^{28-}$ (five cubanes), which exhibit an increasing HER activity with higher number of encapsulated $\mathbf{\{Ni^{II}_4O_4\}}$ cubanes. Bonds belonging to cubane motifs are highlighted in black. Color code: Ni^{II}, lime; P^{V}, purple; O, red; V^{V}, orange.

3. IR-spectra

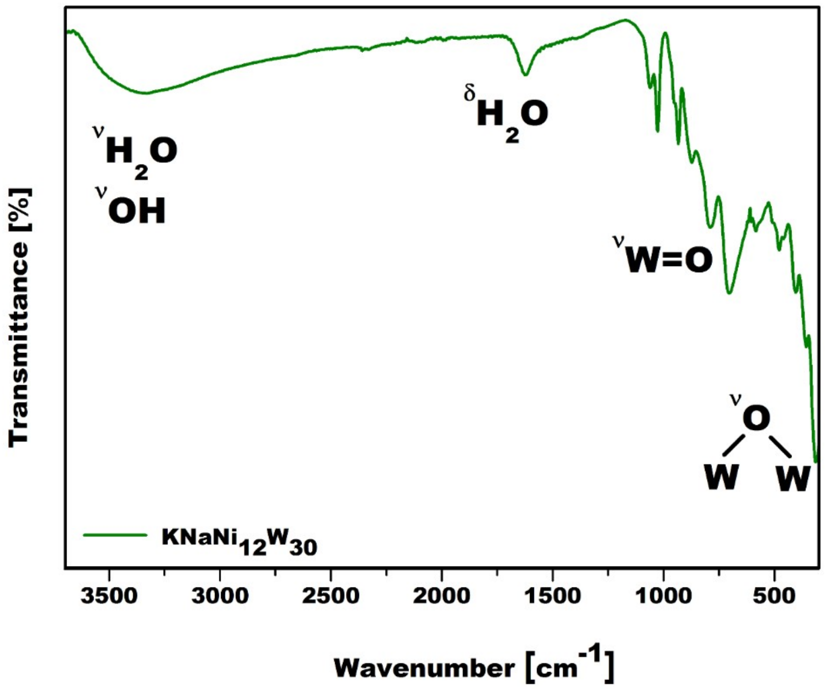

Figure S4. IR-spectrum of $K_{11}Na_{10}$-**{$Ni_{12}W_{30}$}** in the range of 3600 - 300 cm^{-1}. The strong vibrational peaks at 1168 cm^{-1} and 1061 cm^{-1} can be associated with P=O and P-O stretching.[47] The broad vibrational peak at ~3400 cm^{-1} and the sharp peak at ~1600 cm^{-1} are characteristic of stretching vibration of (O-H) and bending vibration of (O-H) in the lattice and coordinated water molecules.

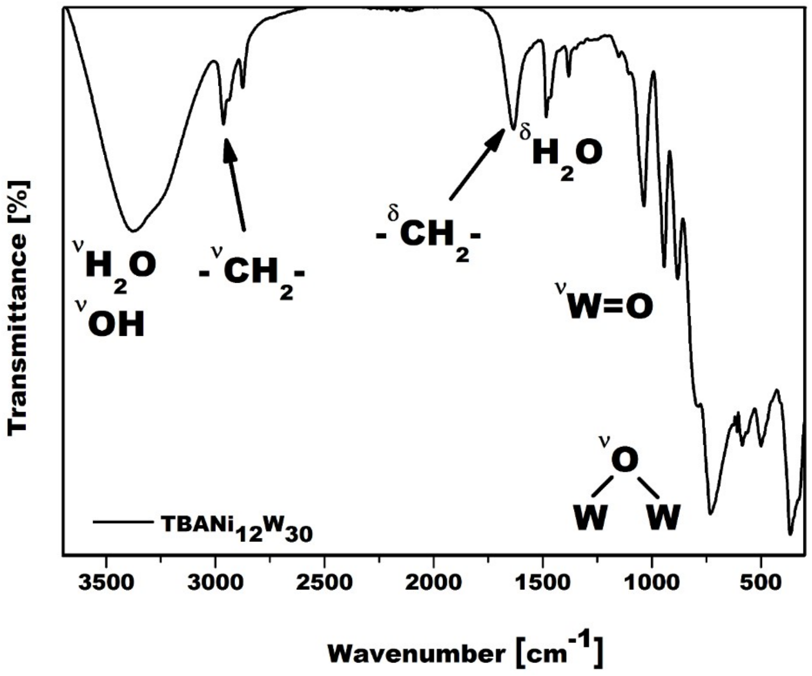

Figure S5. IR-spectrum of $TBA_{13}Na_8$-**{$Ni_{12}W_{30}$}** in the range of 3600 - 300 cm^{-1}.

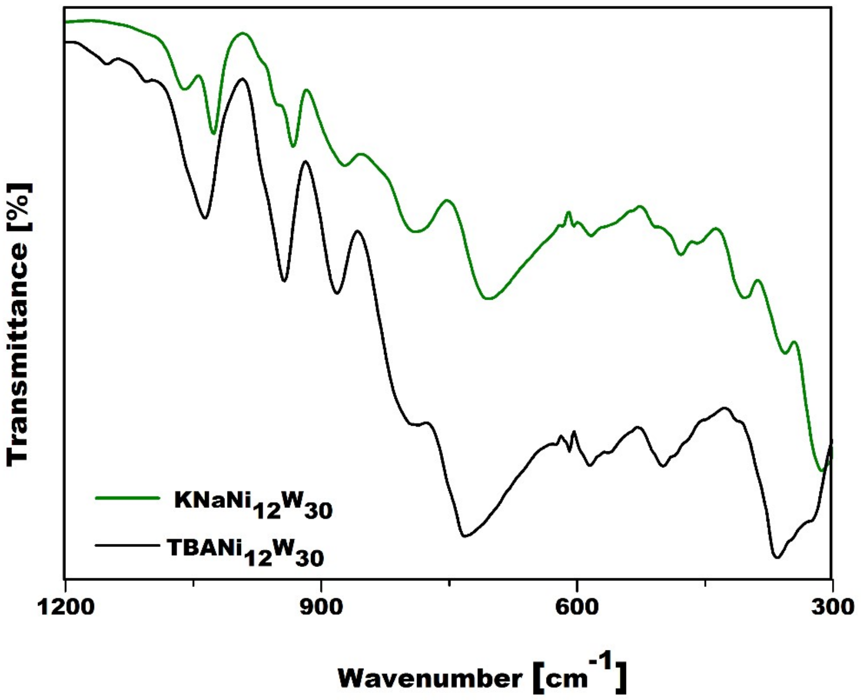

Figure S6. IR-spectra showing the tungsten fingerprint area of $K_{11}Na_{10}$-**{Ni₁₂W₃₀}** (green) and $TBA_{13}Na_{8}$-**$\{Ni_{12}W_{30}\}$** (black) from 1200 – 300 cm^{-1}. The spectra show all the characteristic bands of polyanion **$\{Ni_{12}W_{30}\}$** confirming that the ion-exchange does not change the structure of **$\{Ni_{12}W_{30}\}$**.

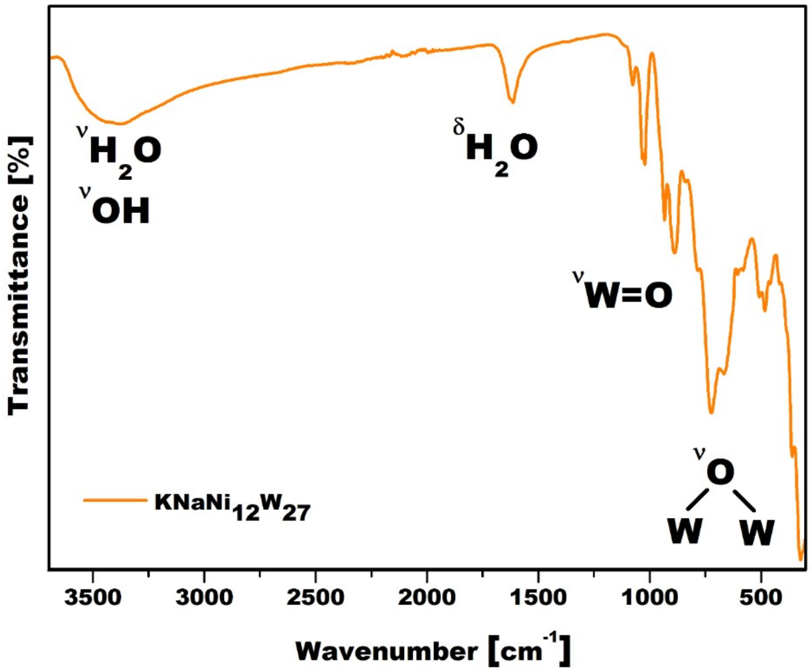

Figure S7. IR-spectrum of $K_{14}Na_{7}$-**$\{Ni_{12}W_{27}\}$** in the range of 3600 - 300 cm^{-1}. The strong vibrational peaks at 1168 cm^{-1} and 1061 cm^{-1} can be associated with P=O and P-O stretching.[47] The broad vibrational peak at ~3400 cm^{-1} and the sharp peak at ~1600 cm^{-1} are the characteristic of stretching vibration of (O-H) and bending vibration of (O-H) in the lattice and coordinated water molecules.

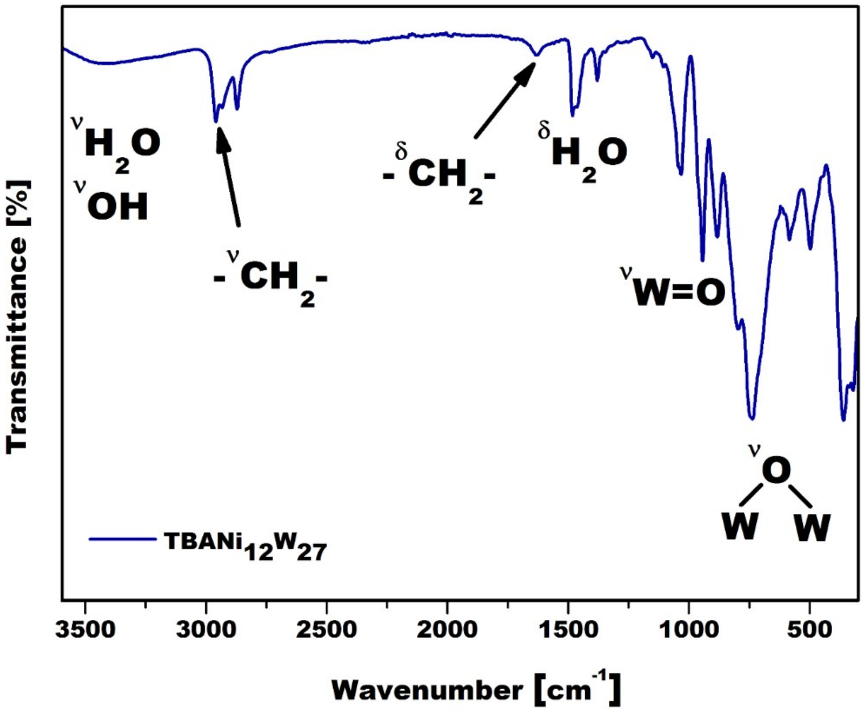

Figure S8. IR-spectrum of $TBA_{13}Na_8$-**$\{Ni_{12}W_{27}\}$** in the range of 3600 - 300 cm^{-1}.

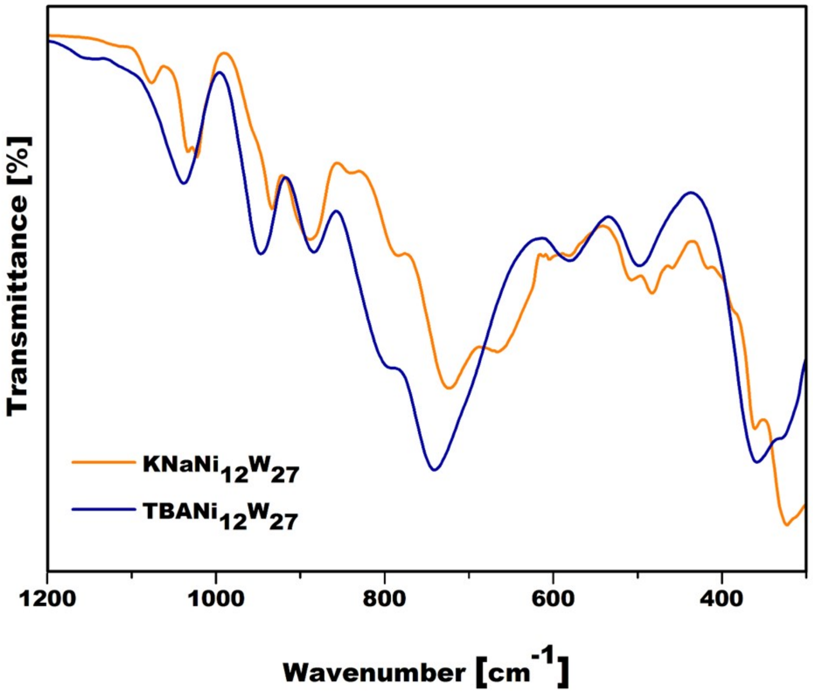

Figure S9. IR-spectra showing the tungsten fingerprint area of $K_{14}Na_7$-**$\{Ni_{12}W_{27}\}$** (yellow) and $TBA_{13}Na_8$-**$\{Ni_{12}W_{27}\}$** (blue) from 1200 – 300 cm^{-1}. The spectra show all the characteristic bands of polyanion **$\{Ni_{12}W_{27}\}$** confirming that the ion-exchange does not change the structure of **$\{Ni_{12}W_{27}\}$**.

Table S3. Attribution and positions of the bands observed in the IR-spectra of $K_{11}Na_{10}$-**{$Ni_{12}W_{30}$}**, $K_{14}Na_7$-**{$Ni_{12}W_{27}$}**, $TBA_{13}Na_8$-**{$Ni_{12}W_{30}$}**, and $TBA_{13}Na_8$-**{$Ni_{12}W_{27}$}**.

Ni-PT	position/range [cm^{-1}]	attribution	intensity
$K_{11}Na_{10}$-**{$Ni_{12}W_{30}$}**	3324.9	$^{\nu}$OH	m
	1618.2	$^{\delta}H_2O$	m
	1058.8, 1027.9	$^{\nu}$P-O	s
	931.4	$^{\nu}$W=O	s
	871.7 – 312.4	$^{\nu}$W=O, $^{\delta}$W-O-W	s
	584.4	$^{\delta}$Ni-O-H	w
	403.1	$^{\nu}$Ni-O	w
$TBA_{13}Na_8$-**{$Ni_{12}W_{30}$}**	3380.9	$^{\nu}$OH	m
	2962.3, 2875.7	-$^{\nu}CH_2$	m
	1633.5	$^{\delta}H_2O$	m
	1483.1, 1380.9	-$^{\delta}CH_2$	m
	1035.7	$^{\nu}$P-O	s
	943.1	$^{\nu}$W=O	s
	881.4 – 366.4	$^{\nu}$W=O, $^{\delta}$W-O-W	s
	584.4	$^{\delta}$Ni-O-H	w
	408.9	$^{\nu}$Ni-O	w
$K_{14}Na_7$-**{$Ni_{12}W_{27}$}**	3373.1	$^{\nu}$OH	m
	1612.2	$^{\delta}H_2O$	w
	1076.2, 1022.2	$^{\nu}$P-O	s
	933.4	$^{\nu}$W=O	s
	887.2 - 324	$^{\nu}$W=O, $^{\delta}$W-O-W	s
	580.5	$^{\delta}$Ni-O-H	w
	416.6	$^{\nu}$Ni-O	w
$TBA_{13}Na_8$-**{$Ni_{12}W_{27}$}**	3384.7	$^{\nu}$OH	w
	2960.4, 2871.7	-$^{\nu}CH_2$	m
	1633.5	$^{\delta}H_2O$	w
	1481.1, 1380.9	-$^{\delta}CH_2$	m

	1031.8	$^{\nu}$P-O	s
	943.1	$^{\nu}$W=O	s
	883.3 – 320.4	$^{\nu}$W=O, $^{\delta}$W-O-W	s
	584.4	$^{\delta}$Ni-O-H	m
	408.9	$^{\nu}$Ni-O	w

4. Thermogravimetric analysis

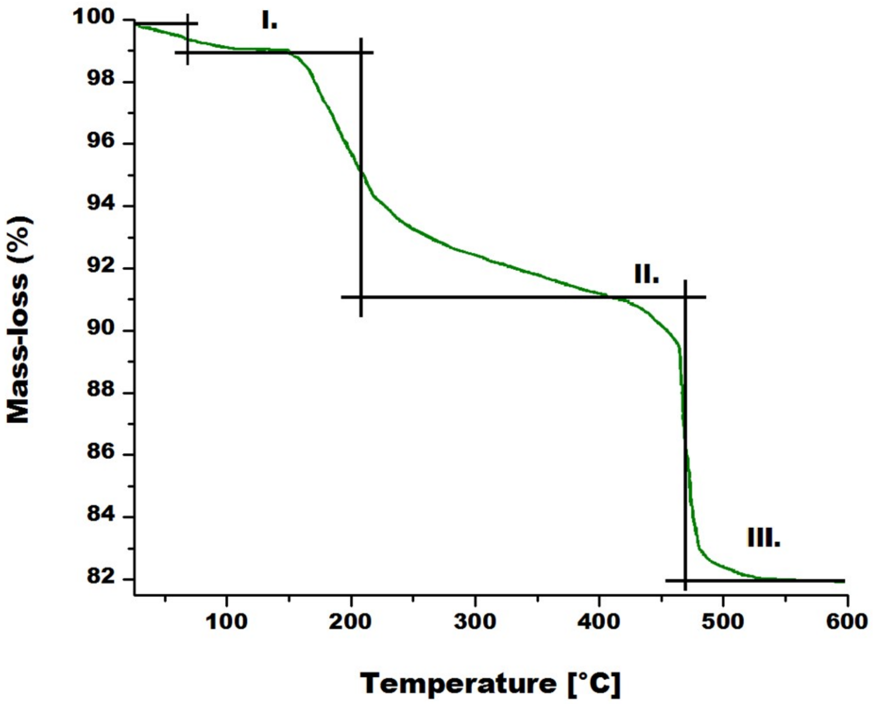

Figure S10. Thermogravimetric curve of $K_{11}Na_{10}$-**{$Ni_{12}W_{30}$}** • 98 H_2O showing three weight-loss steps (**Table S4**).

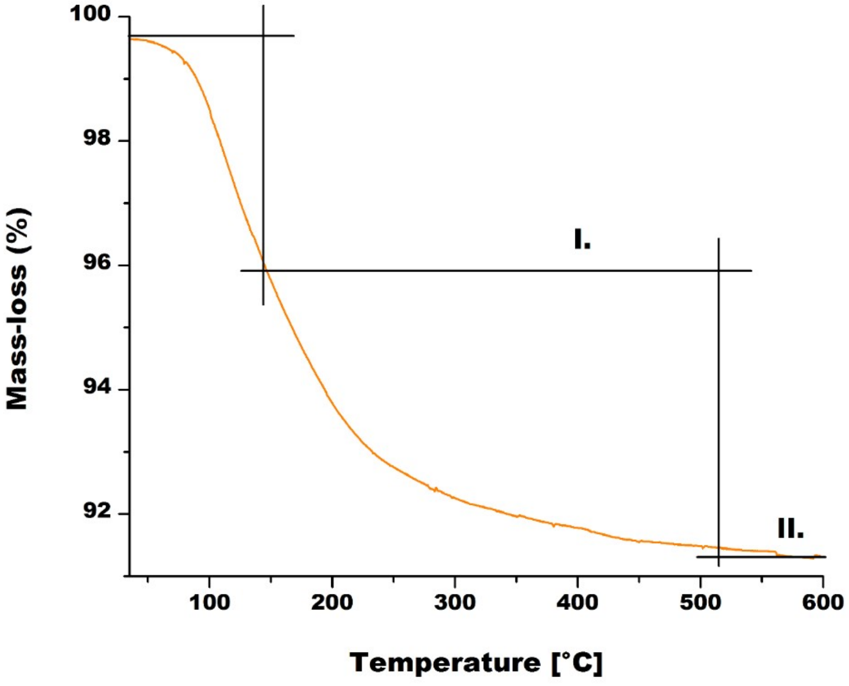

Figure S11. Thermogravimetric curve of $K_{14}Na_{7}$-**{$Ni_{12}W_{27}$}** • 44 H_2O showing two weight-loss steps (**Table S4**).

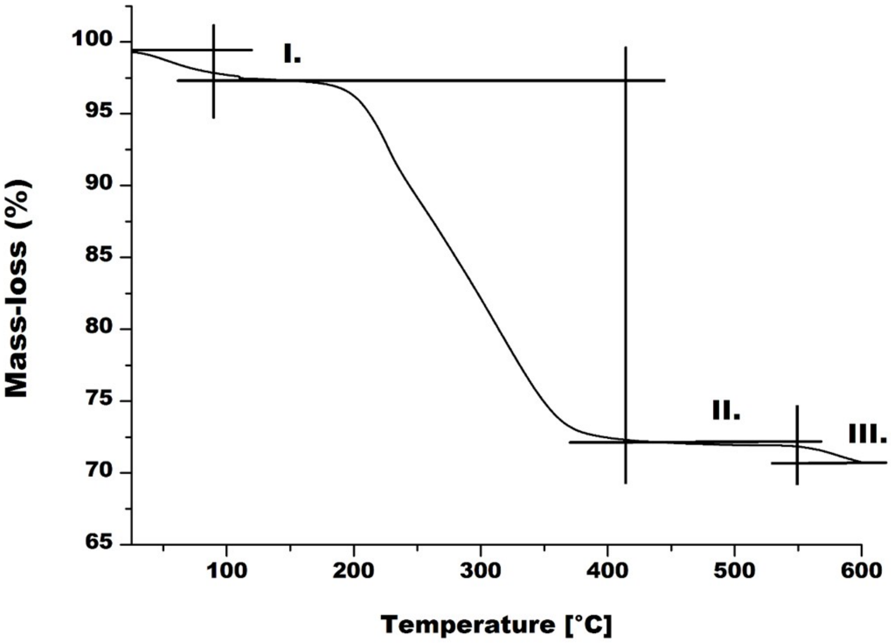

Figure S12. Thermogravimetric curve of $TBA_{13}Na_8$-**$\{Ni_{12}W_{30}\}$ • 13 H_2O** showing three weight-loss steps (**Table S4**).

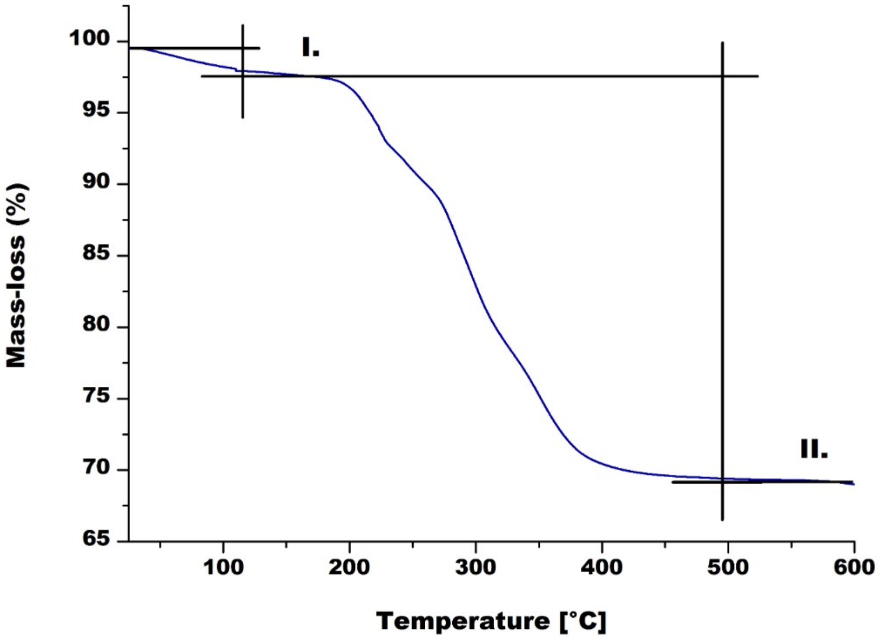

Figure S13. Thermogravimetric curve of $TBA_{13}Na_8$-**$\{Ni_{12}W_{27}\}$ • 11 H_2O** showing two weight-loss steps (**Table S4**).

Table S4. TGA data for $K_{11}Na_{10}$-**$\{Ni_{12}W_{30}\}$ • 98 H_2O,** $K_{14}Na_7$-**$\{Ni_{12}W_{27}\}$ • 44 H_2O**, $TBA_{13}Na_8$-**$\{Ni_{12}W_{30}\}$ • 13 H_2O**, and $TBA_{13}Na_8$-**$\{Ni_{12}W_{27}\}$ • 11 H_2O**.

Compound	Step	T, °C	mass-loss, %	number of molecules corresponding to mass-loss
$K_{11}Na_{10}$-**$\{Ni_{12}W_{30}\}$ • 98 H_2O**	I	20-50	1.0	5 H_2O
	II	50-350	7.9	43 H_2O
	III	350-650	9.1	50 H_2O
$K_{14}Na_7$-**$\{Ni_{12}W_{27}\}$ • 44 H_2O**	I	35-150	4.2	22 H_2O
	II	150-600	4.3	22 H_2O
$TBA_{13}Na_8$-**$\{Ni_{12}W_{30}\}$ • 13 H_2O**	I	25-150	2.7	13 H_2O
	II	150-400	25.3	12 TBA
	III	400-600	2.8	1 TBA
$TBA_{13}Na_8$-**$\{Ni_{12}W_{27}\}$ • 11 H_2O**	I	25-150	2.3	11 H_2O
	II	150-550	28.4	13 TBA

5. Single-Crystal X-ray Diffraction (SXRD)

Single crystal X-ray diffraction studies revealed that **{$Ni_{12}W_{30}$}** crystallizes in the triclinic space group ***P*$\bar{1}$** (**Tables S5-S7**, CCDC 2102166), whereas **{$Ni_{12}W_{27}$}** crystallizes in the monoclinic space group $C_{2/c}$ (**Tables S8**, **S9**, CCDC 2102167). Both anions have a Ni_{12} metal-oxo cluster stabilized by three tri-lacunary phosphotungstate ligands in common. In **{$Ni_{12}W_{30}$}** (**Figure S14A**), three *B*-type $[PW_9O_{34}]^{9-}$ ligands (**Figure S14B**) encapsulate a Ni_{12} scaffold composed of three tri- protonated $\{Ni^{II}{}_4O(OH)_3\}$ cubanes, which are connected by one PO_4^{3-} linker, forming a central $\{Ni^{II}{}_3O_4\}$ quasi-cubane. All 12 Ni^{II} centers display distorted octahedral coordination geometry with Ni-O bond lengths of 1.995(1) to 2.263(9) Å. Three tetrahedrally coordinated WO_4^{2-} capping ligands that are rarely found in the literature[48] complete the structure of **{$Ni_{12}W_{30}$}** (**Figure S14C**). **{$Ni_{12}W_{27}$}** (**Figure S14D**) is composed of one [*B*-$PW_9O_{34}]^{9-}$[49] (**Figure S14B**) and two [*A*- $PW_9O_{34}]^{9-}$ isomers[50] (**Figure S14E**) that encapsulate a Ni_{12} core, which can be regarded as a structural isomer of the Ni_{12} core in **{$Ni_{12}W_{30}$}**. Attributed to the different connectivity types between the Ni^{II} metal centers and the POT isomers (**Figure S14B, E**), the Ni_{12} core in **{$Ni_{12}W_{27}$}** comprises one $\{Ni^{II}{}_4O(OH)_3\}$ cubane connected to the [*B*-$PW_9O_{34}]^{9-}$ ligand and two tetrahedral $\{Ni_4(OH)_3\}$ units coordinated to the two [*A*-$PW_9O_{34}]^{9-}$ isomers. One PO_4^{3-} linker connects the $\{Ni^{II}{}_4O(OH)_3\}$ to both $\{Ni_4(OH)_3\}$ motifs, forming a central $\{Ni^{II}{}_3O_4\}$ quasi-cubane. All Ni^{II} display distorted octahedral coordination geometry with Ni-O bond lengths of 1.998(1) to 2.314(1) Å. The structure of **{$Ni_{12}W_{27}$}** is completed by three tetrahedral monoprotonated $[HPO_4]^{2-}$ capping ligands (**Figure S14F**). Note that **{$Ni_{12}W_{27}$}** represents a rare example of a POT composed of two different types of lacunary ligand-isomers (**Table S1**).

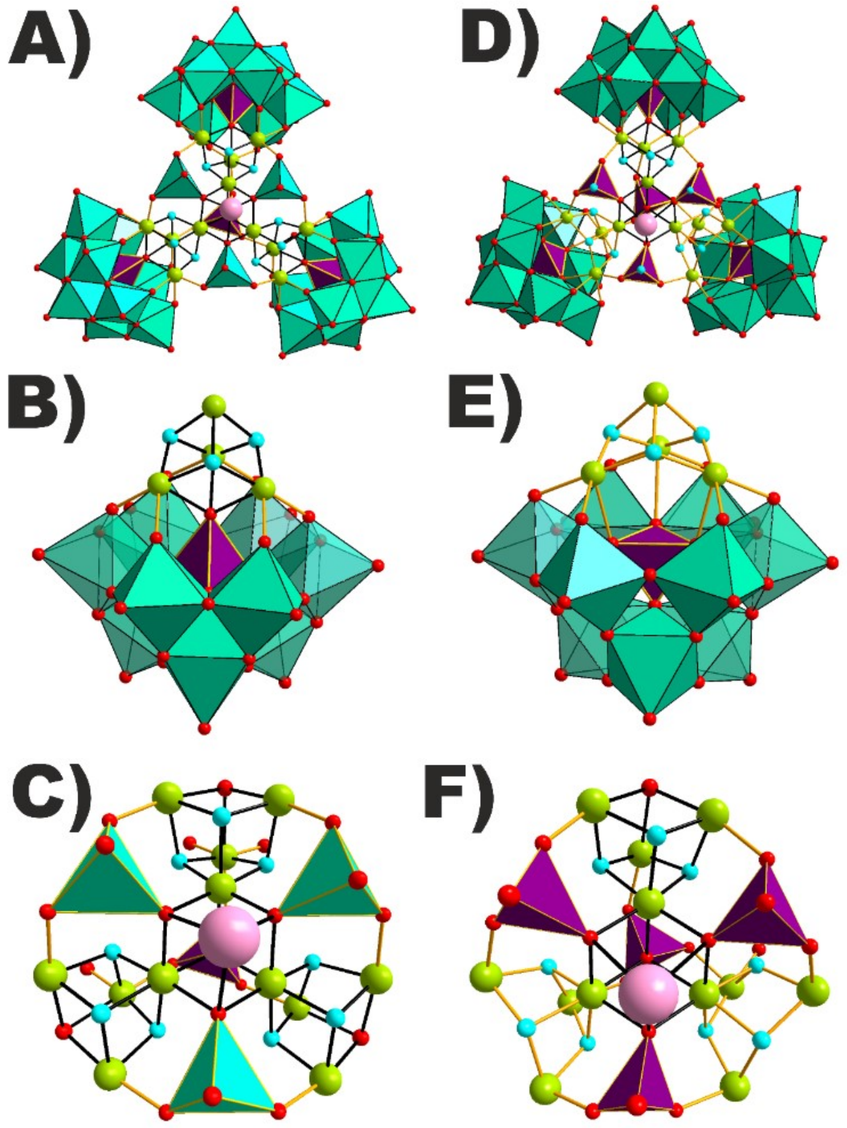

Figure S14. Polyhedral representation of **A) {$Ni_{12}W_{30}$}**, which is composed entirely of **B)** [B-PW_9O_{34}]$^{9-}$ units enclosing a **C)** Ni_{12} scaffold that comprises three tri- protonated {$Ni^{II}_4O(OH)_3$} cubanes. In contrast, the architecture of **D) {$Ni_{12}W_{27}$}** represents a trimer of one [*B*-PW_9O_{34}]$^{9-}$ and two **E)** [*A*-PW_9O_{34}]$^{9-}$ isomers leading to a **F)** Ni_{12} core with two {$Ni^{II}_4(OH)_3$} tetrahedra and one {$Ni^{II}_4O(OH)_3$}. Color Code, Polyhedra: turquoise for {WO_6}/{WO_4} and purple for {PO_4}. Balls: red for oxygen lime green for NiII and sky-blue for OH^- ions, respectively.

Table S5. Experimental parameter and CCDC-Codes.

Sample	Source	Temp.	Detector Distance	Time/ Frame	#Frames	Frame width	CCDC
		[K]	[mm]	[s]		[°]	
$K_{11}Na_{10}$-**{$Ni_{12}W_{30}$}**	Mo	100	40	25	3062	0.5	2102166
$K_{14}Na_7$-**{$Ni_{12}W_{27}$}**	Mo	100	30	10	2293	0.5	2102167

Table S6. Sample and crystal data of $K_{11}Na_{10}$-**{$Ni_{12}W_{30}$}**.

**Chemical formula**	$K_{11}Na_{10}Ni_{12}W_{30}H_{205}O_{225}P_4$	**Crystal system**	triclinic	
**Formula weight [g/mol]**	10809.88	**Space group**	$P\bar{1}$	
**Temperature [K]**	100.0	**Z**	4	
**Measurement method**	φ and ω scans	**Volume [Å³]**	17317.0(6)	
**Radiation (Wavelength [Å])**	MoKα (λ = 0.71073)	**Unit cell dimensions [Å] and [°]**	22.0527(5)	85.0088(8)
**Crystal size / [mm³]**	0.1 × 0.07 × 0.03		22.7367(5)	84.9745(8)
**Crystal habit**	clear colorless rod		35.1499(7)	81.5541(8)
**Density (calculated) / [g/cm³]**	3.782	**Absorption coefficient / [mm⁻¹]**	21.431	
**Abs. correction T_{min}**	0.223	**Abs. correction T_{max}**	0.566	
**Abs. correction type**	multi-scan	**F(000) [e⁻]**	17444.0	

Table S7. Data collection and structure refinement of $K_{11}Na_{10}$-**{$Ni_{12}W_{30}$}**.

**Index ranges**	-26 ≤ h ≤ 26, -27 ≤ k ≤ 27, -42 ≤ l ≤ 42	**Theta range for data collection [°]**	4.26 to 50.7	
**Reflections number**	729078	**Data / restraints / parameters**	63391/216/4526	
**Refinement method**	Least squares	**Final R indices**	all data	R_1 = 0.0542, wR_2 = 0.1112
**Function minimized**	$\Sigma\ w(F_o^2 - F_c^2)^2$		I>2σ(I)	R_1 = 0.0433, wR_2 = 0.1045
**Goodness-of-fit on F^2**	1.055	**Weighting scheme**	$w=1/[\sigma^2(F_o^2)+(0.0409P)^2+761.5953P]$	

**Largest diff. peak and hole [e Å^{-3}]**	5.68/-3.46		where P=(F_o^2+2F_c^2)/3

Table S8. Sample and crystal data of $K_{14}Na_7$-**{$Ni_{12}W_{27}$}**.

**Chemical formula**	$K_{14}Na_7Ni_{12}W_{27}H_{100}O_{171}P_7$	**Crystal system**	monoclinic	
**Formula weight [g/mol]**	9429.81	**Space group**	*C*2/*c*	
**Temperature [K]**	100.0	**Z**	8	
**Measurement method**	φ and ω scans	**Volume [Å^3]**	29947(5)	
**Radiation (Wavelength [Å])**	MoKα (λ = 0.71073)	**Unit cell dimensions [Å] and [°]**	37.424(4)	90
**Crystal size / [mm^3]**	0.15 × 0.12 × 0.08		22.3451(18)	117.365(7)
**Crystal habit**	clear yellow plate		40.324(4)	90
**Density (calculated) / [g/cm^3]**	3.980	**Absorption coefficient / [mm^{-1}]**	22.436	
**Abs. correction T_{min}**	0.134	**Abs. correction T_{max}**	0.267	
**Abs. correction type**	multi-scan	**F(000) [e$^-$]**	31795.0	

Table S9. Data collection and structure refinement of $K_{14}Na_7$-**{$Ni_{12}W_{27}$}**.

**Index ranges**	-45 ≤ h ≤ 45, -26 ≤ k ≤ 26, -48 ≤ l ≤ 48	**Theta range for data collection [°]**	4.104 to 50.832	
**Reflections number**	523135	**Data / restraints / parameters**	27499/84/2062	
**Refinement method**	Least squares	**Final R indices**	all data	R_1 = 0.0626, wR_2 = 0.1481
**Function minimized**	Σ w(F_o^2 - F_c^2)^2		I>2σ(I)	R_1 = 0.0516, wR_2 = 0.1365
**Goodness-of-fit on F^2**	1.024	**Weighting scheme**	w=1/[σ^2(F_o^2)+(0.0801P)^2+1356.9673P]	
**Largest diff. peak and hole [e Å^{-3}]**	4.48/-2.10		where P=(F_o^2+2F_c^2)/3	

Table S10. Bond lengths and angles of Ni^{II} metal centers in $K_{11}Na_{10}$-**{$Ni_{12}W_{30}$}**.

Ni-center	Distances [Å]		Angles [°]	
Ni1	Ni1-O29	2.066(13)	O15-Ni1-O1	159.3(5)
	Ni1-O28	2.080(13)	O15-Ni1-O28	89.8(5)
	Ni1-O15	2.017(13)	O15-Ni1-O29	90.4(5)
	Ni1-O27	2.004(13)	O15-Ni1-O30	96.7(5)
	Ni1-O30	2.044(11)	O27-Ni1-O1	101.1(5)
	Ni1-O1	2.311(11)	O27-Ni1-O15	94.1(5)
			O27-Ni1-O28	96.0(5)
			O27-Ni1-O29	173.9(5)
			O27-Ni1-O30	80.7(5)
			O28-Ni1-O1	74.8(5)
			O29-Ni1-O1	75.7(5)
			O29-Ni1-O28	88.2(5)
			O30-Ni1-O1	99.6(4)
			O30-Ni1-O28	172.9(5)
			O30-Ni1-O29	94.6(5)
Ni2	Ni2-O3	2.313(13)	O25-Ni2-O3	161.1(4)
	Ni2-O25	2.035(11)	O25-Ni2-O31	96.6(5)
	Ni2-O27	2.026(13)	O25-Ni2-O32	87.5(5)
	Ni2-O31	2.046(12)	O27-Ni2-O3	100.8(5)
	Ni2-O32	2.050(12)	O27-Ni2-O25	88.9(5)
	Ni2-O33	1.996(10)	O27-Ni2-O31	172.7(6)
			O27-Ni2-O32	95.9(5)
			O31-Ni2-O3	75.3(5)
			O31-Ni2-O32	89.2(5)
			O32-Ni2-O3	75.5(5)
			O33-Ni2-O3	100.1(4)
			O33-Ni2-O25	97.4(4)

			O33-Ni2-O27	81.0(5)
			O33-Ni2-O31	93.4(5)
			O33-Ni2-O32	174.2(5)
**Ni3**	Ni3-O2	2.295(12)	O21-Ni3-O2	158.0(4)
	Ni3-O21	2.009(10)	O21-Ni3-O30	99.7(4)
	Ni3-O30	2.013(10)	O21-Ni3-O33	91.0(4)
	Ni3-O33	2.032(10)	O21-Ni3-O34	94.6(5)
	Ni3-O34	2.040(13)	O21-Ni3-O35	85.5(5)
	Ni3-O35	2.060(11)	O30-Ni3-O2	100.4(4)
			O30-Ni3-O33	81.8(4)
			O30-Ni3-O34	94.8(5)
			O30-Ni3-O35	173.8(5)
			O33-Ni3-O2	100.6(4)
			O33-Ni3-O34	173.9(5)
			O33-Ni3-O35	94.8(5)
			O34-Ni3-O2	74.9(5)
			O34-Ni3-O35	88.2(5)
			O35-Ni3-O2	75.1(5)
**Ni4**	Ni4-O14	2.106(13)	O22-Ni4-O14	85.0(5)
	Ni4-O22	2.102(10)	O26-Ni4-O14	89.6(5)
	Ni4-O26	2.096(10)	O26-Ni4-O22	80.9(4)
	Ni4-O27	2.052(13)	O27-Ni4-O14	94.2(5)
	Ni4-O30	2.063(11)	O27-Ni4-O22	173.8(5)
	Ni4-O33	2.060(9)	O27-Ni4-O26	105.2(5)
			O27-Ni4-O30	79.1(5)
			O27-Ni4-O33	78.9(5)
			O30-Ni4-O14	100.0(5)
			O30-Ni4-O22	94.9(4)

			O30-Ni4-O26	169.2(4)
			O33-Ni4-O14	173.0(5)
			O33-Ni4-O22	102.0(4)
			O33-Ni4-O26	91.2(4)
			O33-Ni4-O30	79.9(4)
**Ni5**	Ni5-O6	2.254(12)	O13-Ni5-O6	166.0(5)
	Ni5-O13	2.023(13)	O36-Ni5-O6	91.7(5)
	Ni5-O36	1.990(12)	O36-Ni5-O13	98.5(6)
	Ni5-O37	2.009(11)	O36-Ni5-O37	87.7(5)
	Ni5-O38	1.997(10)	O36-Ni5-O38	171.9(5)
	Ni5-O39	1.982(9)	O37-Ni5-O6	94.1(5)
			O37-Ni5-O13	95.8(5)
			O38-Ni5-O6	80.4(4)
			O38-Ni5-O13	89.0(5)
			O38-Ni5-O37	94.7(4)
			O39-Ni5-O6	78.5(4)
			O39-Ni5-O13	91.4(5)
			O39-Ni5-O36	92.8(4)
			O39-Ni5-O37	172.6(5)
			O39-Ni5-O38	83.8(4)
**Ni6**	Ni6-O6	2.274(13)	O17-Ni6-O6	167.1(4)
	Ni6-O17	2.046(11)	O38-Ni6-O6	79.9(4)
	Ni6-O38	2.000(11)	O38-Ni6-O17	92.2(4)
	Ni6-O40	2.010(11)	O38-Ni6-O40	93.9(4)
	Ni6-O41	1.990(12)	O40-Ni6-O6	91.8(5)
	Ni6-O42	1.985(10)	O40-Ni6-O17	99.0(5)
			O41-Ni6-O6	91.7(5)
			O41-Ni6-O17	95.9(5)

			O41-Ni6-O38	171.5(5)
			O41-Ni6-O40	87.5(5)
			O42-Ni6-O6	78.2(4)
			O42-Ni6-O17	90.9(4)
			O42-Ni6-O38	83.9(4)
			O42-Ni6-O40	170.0(5)
			O42-Ni6-O41	93.3(5)
**Ni7**	Ni7-O23	2.044(10)	O23-Ni7-O6	163.7(4)
	Ni7-O39	1.995(10)	O39-Ni7-O6	78.3(4)
	Ni7-O42	2.033(10)	O39-Ni7-O23	86.0(4)
	Ni7-O97	2.003(12)	O39-Ni7-O42	82.7(4)
	Ni7-O100	2.035(11)	O39-Ni7-O97	95.4(5)
	Ni7-O6	2.253(12)	O39-Ni7-O100	172.7(5)
			O42-Ni7-O6	77.8(4)
			O42-Ni7-O23	96.0(4)
			O42-Ni7-O100	93.7(4)
			O97-Ni7-O6	94.5(5)
			O97-Ni7-O23	91.3(5)
			O97-Ni7-O42	172.3(5)
			O97-Ni7-O100	87.4(5)
			O100-Ni7-O6	94.8(5)
			O100-Ni7-O23	100.7(5)
**Ni8**	Ni8-O14	2.063(13)	O14-Ni8-O19	95.5(5)
	Ni8-O19	2.079(10)	O14-Ni8-O22	86.7(5)
	Ni8-O22	2.079(10)	O22-Ni8-O19	86.6(4)
	Ni8-O38	2.021(10)	O38-Ni8-O14	98.9(5)
	Ni8-O39	2.029(10)	O38-Ni8-O19	93.5(4)
	Ni8-O42	1.997(10)	O38-Ni8-O22	174.4(4)

			O38-Ni8-O39	82.0(4)
			O39-Ni8-O14	88.1(5)
			O39-Ni8-O19	174.7(4)
			O39-Ni8-O22	97.5(4)
			O42-Ni8-O14	170.3(5)
			O42-Ni8-O19	93.9(4)
			O42-Ni8-O22	91.4(4)
			O42-Ni8-O38	83.0(4)
			O42-Ni8-O39	82.7(4)
**Ni9**	Ni9-O12	2.317(11)	O18-Ni9-O12	159.9(4)
	Ni9-O18	2.012(11)	O18-Ni9-O44	90.0(5)
	Ni9-O43	1.999(10)	O18-Ni9-O45	89.7(5)
	Ni9-O44	2.074(12)	O18-Ni9-O46	94.2(5)
	Ni9-O45	2.050(12)	O43-Ni9-O12	98.7(4)
	Ni9-O46	2.046(11)	O43-Ni9-O18	96.7(4)
			O43-Ni9-O44	94.6(4)
			O43-Ni9-O45	172.9(5)
			O43 Ni9 O46	81.2(4)
			O44-Ni9-O12	76.0(4)
			O45-Ni9-O12	75.8(4)
			O45-Ni9-O44	88.5(5)
			O46-Ni9-O12	100.8(4)
			O46-Ni9-O44	174.4(5)
			O46-Ni9-O45	95.2(5)
**Ni10**	Ni10-O10	2.372(11)	O24-Ni10-O10	158.4(4)
	Ni10-O24	2.032(11)	O24-Ni10-O46	99.7(4)
	Ni10-O46	2.034(11)	O24-Ni10-O48	86.5(5)
	Ni10-O47	2.026(12)	O46-Ni10-O10	99.8(4)

	Ni10-O48	2.063(11)	O46-Ni10-O48	173.1(5)
	Ni10-O49	2.033(11)	O47-Ni10-O10	74.4(4)
			O47-Ni10-O24	95.0(5)
			O47-Ni10-O46	94.2(5)
			O47-Ni10-O48	88.1(5)
			O47-Ni10-O49	170.6(5)
			O48-Ni10-O10	74.6(4)
			O49-Ni10-O10	97.8(4)
			O49-Ni10-O24	94.1(5)
			O49-Ni10-O46	81.7(4)
			O49-Ni10-O48	95.0(5)
**Ni11**	Ni11-O19	2.077(10)	O19-Ni11-O22	84.8(4)
	Ni11-O22	2.153(10)	O19-Ni11-O26	88.1(4)
	Ni11-O26	2.106(10)	O19-Ni11-O43	92.8(4)
	Ni11-O43	2.105(10)	O26-Ni11-O22	79.5(4)
	Ni11-O46	2.057(11)	O26-Ni11-O43	175.4(4)
	Ni11-O49	2.066(11)	O43-Ni11-O22	105.0(4)
			O46-Ni11-O19	101.8(4)
			O46-Ni11-O22	172.5(4)
			O46-Ni11-O26	97.0(4)
			O46-Ni11-O43	78.5(4)
			O46-Ni11-O49	80.3(4)
			O49-Ni11-O19	170.8(4)
			O49-Ni11-O22	93.7(4)
			O49-Ni11-O26	100.5(4)
			O49-Ni11-O43	78.9(4)
**Ni12**	Ni12-O11	2.282(12)	O20-Ni12-O11	160.8(4)
	Ni12-O20	1.975(11)	O20-Ni12-O43	89.3(4)

	Ni12-O43	2.015(11)	O20-Ni12-O49	97.5(4)
	Ni12-O49	2.024(11)	O20-Ni12-O50	86.5(5)
	Ni12-O50	2.054(11)	O20-Ni12-O51	96.1(5)
	Ni12-O51	2.045(12)	O43-Ni12-O11	100.2(4)
			O43-Ni12-O49	82.0(4)
			O43-Ni12-O50	95.2(5)
			O43-Ni12-O51	173.5(5)
			O49-Ni12-O11	100.3(4)
			O49-Ni12-O50	175.0(5)
			O49-Ni12-O51	93.6(5)
			O50-Ni12-O11	76.1(4)
			O51-Ni12-O11	75.7(5)
			O51-Ni12-O50	88.8(5)

Table S11. Bond lengths and angles of Ni^{II} metal centers in $K_{14}Na_7$-**{$Ni_{12}W_{27}$}**.

Ni-center	Distances [Å]		Angles [°]	
Ni1	Ni1-O3	2.182(8)	O28-Ni1-O3	92.4(3)
	Ni1-O28	2.013(10)	O28-Ni1-O37	97.6(4)
	Ni1-O37	2.019(9)	O28-Ni1-O40	92.5(4)
	Ni1-O40	2.071(9)	O28-Ni1-O351	86.0(4)
	Ni1-O351	2.018(10)	O37-Ni1-O3	81.2(3)
	Ni1-O358	2.004(9)	O37-Ni1-O40	91.8(4)
			O40-Ni1-O3	171.9(4)
			O351-Ni1-O3	94.4(3)
			O351-Ni1-O37	174.4(4)
			O351-Ni1-O40	92.3(4)
			O358-Ni1-O3	82.1(3)
			O358-Ni1-O28	174.5(3)
			O358-Ni1-O37	81.7(3)
			O358-Ni1-O40	93.0(4)
			O358-Ni1-O351	94.3(4)
Ni2	Ni2-O3	2.263(9)	O7BA-Ni2-O3	167.9(3)
	Ni2-O7BA	2.054(9)	O22-Ni2-O3	91.7(3)
	Ni2-O22	2.008(8)	O22-Ni2-O7BA	99.1(3)
	Ni2-O36	2.008(9)	O36-Ni2-O3	80.3(3)
	Ni2-O350	2.008(9)	O36-Ni2-O7BA	93.7(4)
	Ni2-O358	2.006(8)	O36-Ni2-O22	93.0(3)
			O36-Ni2-O350	171.9(4)
			O350-Ni2-O3	91.6(3)
			O350-Ni2-O7BA	94.3(3)
			O350-Ni2-O22	86.6(3)

			O358-Ni2-O3	80.0(3)
			O358-Ni2-O7BA	89.1(3)
			O358-Ni2-O22	171.7(4)
			O358 Ni2 O36	85.5(4)
			O358 Ni2 O350	93.7(4)
Ni3	Ni3-O3	2.198(9)	O25-Ni3-O3	93.1(3)
	Ni3-O25	2.013(8)	O25-Ni3-O34	86.9(4)
	Ni3-O34	2.022(10)	O25-Ni3-O36	94.0(4)
	Ni3-O36	2.014(9)	O25-Ni3-O361	91.1(4)
	Ni3-O37	1.998(8)	O34-Ni3-O3	92.2(4)
	Ni3-O361	2.075(9)	O34-Ni3-O361	94.7(4)
			O36-Ni3-O3	81.8(3)
			O36-Ni3-O34	174.0(4)
			O36-Ni3-O361	91.2(4)
			O37-Ni3-O3	81.2(3)
			O37-Ni3-O25	173.4(4)
			O37-Ni3-O34	96.7(4)
			O37-Ni3-O36	81.9(3)
			O37-Ni3-O361	94.1(4)
			O361-Ni3-O3	172.1(4)
Ni4	Ni4-O36	2.032(9)	O36-Ni4-O89	93.1(3)
	Ni4-O37	2.023(9)	O36-Ni4-O368	95.6(3)
	Ni4-O89	2.132(8)	O36-Ni4-O370	174.9(4)
	Ni4-O358	2.024(9)	O37-Ni4-O36	80.8(4)
	Ni4-O368	2.085(9)	O37-Ni4-O89	173.9(3)
	Ni4-O370	2.057(8)	O37-Ni4-O358	81.1(4)
			O37-Ni4-O368	94.0(4)

			O37-Ni4-O370	98.9(3)
			O358-Ni4-O36	84.4(3)
			O358-Ni4-O89	97.9(3)
			O358-Ni4-O368	175.0(4)
			O358-Ni4-O370	90.5(3)
			O368-Ni4-O89	87.1(3)
			O370-Ni4-O89	87.1(3)
			O370-Ni4-O368	89.6(3)
**Ni5**	Ni5-O43	2.075(10)	O43-Ni5-O48	172.4(4)
	Ni5-O48	2.198(10)	O71-Ni5-O43	92.5(4)
	Ni5-O71	2.006(9)	O71-Ni5-O48	93.1(4)
	Ni5-O81	2.014(9)	O71-Ni5-O81	86.8(4)
	Ni5-O82	2.010(9)	O71-Ni5-O82	173.8(4)
	Ni5-O84	2.008(9)	O71-Ni5-O84	94.2(4)
			O81-Ni5-O43	93.4(4)
			O81-Ni5-O48	92.0(4)
			O82-Ni5-O43	92.3(4)
			O82-Ni5-O48	81.9(3)
			O82-Ni5-O81	96.9(4)
			O84-Ni5-O43	92.6(4)
			O84-Ni5-O48	81.8(4)
			O84-Ni5-O81	173.8(4)
			O84-Ni5-O82	81.6(3)
**Ni6**	Ni6-O48	2.203(9)	O77-Ni6-O48	93.1(4)
	Ni6-O77	2.018(9)	O77-Ni6-O80	94.1(4)
	Ni6-O79	1.995(10)	O79-Ni6-O48	92.0(4)

	Ni6-O80	2.099(9)	O79-Ni6-O77	87.0(4)
	Ni6-O82	2.003(9)	O79-Ni6-O80	91.7(4)
	Ni6-O83	2.005(9)	O79-Ni6-O82	172.4(4)
			O79-Ni6-O83	92.6(4)
			O80-Ni6-O48	172.1(4)
			O82-Ni6-O48	81.9(4)
			O82-Ni6-O77	97.9(4)
			O82-Ni6-O80	93.8(4)
			O82-Ni6-O83	82.0(4)
			O83-Ni6-O48	81.3(4)
			O83-Ni6-O77	174.4(4)
			O83-Ni6-O80	91.6(4)
**Ni7**	Ni7-O48	2.250(9)	O65-Ni7-O48	92.7(4)
	Ni7-O65	2.008(10)	O65-Ni7-O68	86.3(4)
	Ni7-O68	2.039(9)	O65-Ni7-O83	93.7(4)
	Ni7-O83	2.019(9)	O65-Ni7-O85	97.1(4)
	Ni7-O84	2.006(9)	O68-Ni7-O48	92.6(4)
	Ni7-O85	2.051(9)	O68-Ni7-O85	94.9(4)
			O83-Ni7-O48	79.8(3)
			O83-Ni7-O68	172.4(4)
			O83-Ni7-O85	92.6(3)
			O84-Ni7-O48	80.6(4)
			O84-Ni7-O65	173.3(4)
			O84-Ni7-O68	94.3(4)
			O84-Ni7-O83	84.7(4)
			O84-Ni7-O85	89.6(4)
			O85-Ni7-O48	168.0(3)

**Ni8**	Ni8-O82	2.019(9)	O82-Ni8-O83	81.2(3)
	Ni8-O83	2.021(9)	O82-Ni8-O84	80.6(3)
	Ni8-O84	2.039(8)	O82-Ni8-O88	95.2(3)
	Ni8-O88	2.084(8)	O82-Ni8-O89	173.5(3)
	Ni8-O89	2.106(8)	O82-Ni8-O368	97.8(3)
	Ni8-O368	2.055(9)	O83-Ni8-O84	83.8(4)
			O83-Ni8-O88	95.5(4)
			O83-Ni8-O89	92.4(3)
			O83-Ni8-O368	174.6(3)
			O84-Ni8-O88	175.8(4)
			O84-Ni8-O89	97.9(3)
			O84-Ni8-O368	90.8(4)
			O88-Ni8-O89	86.2(3)
			O368-Ni8-O88	89.9(3)
			O368-Ni8-O89	88.6(3)
**Ni9**	Ni9-O46	2.083(10)	O46-Ni9-O93	172.9(3)
	Ni9-O93	2.215(9)	O118-Ni9-O46	93.6(4)
	Ni9-O118	2.010(9)	O118-Ni9-O93	92.6(3)
	Ni9-O120	2.012(9)	O118-Ni9-O120	87.3(4)
	Ni9-O126	2.000(8)	O120-Ni9-O46	91.9(4)
	Ni9-O127	2.003(9)	O120-Ni9-O93	92.0(3)
			O126-Ni9-O46	92.5(4)
			O126-Ni9-O93	81.3(3)
			O126-Ni9-O118	173.9(4)
			O126-Ni9-O120	93.4(3)
			O126-Ni9-O127	81.4(3)
			O127-Ni9-O46	93.8(4)
			O127-Ni9-O93	81.8(3)

			O127-Ni9-O118	97.4(4)
			O127-Ni9-O120	172.4(4)
Ni10	Ni10-O86	2.073(9)	O86-Ni10-O93	170.7(4)
	Ni10-O93	2.200(9)	O110-Ni10-O86	94.0(4)
	Ni10-O110	2.031(9)	O110-Ni10-O93	92.8(4)
	Ni10-O113	2.009(9)	O113-Ni10-O86	94.7(4)
	Ni10-O124	2.010(8)	O113-Ni10-O93	92.1(4)
	Ni10-O127	2.018(9)	O113-Ni10-O110	85.9(4)
			O113-Ni10-O124	174.5(4)
			O113-Ni10-O127	97.5(4)
			O124-Ni10-O86	90.8(4)
			O124-Ni10-O93	82.3(3)
			O124-Ni10-O110	94.7(4)
			O124-Ni10-O127	81.3(4)
			O127-Ni10-O86	91.0(4)
			O127-Ni10-O93	81.8(3)
			O127-Ni10-O110	173.7(4)
Ni11	Ni11-O93	2.250(9)	O107-Ni11-O93	91.6(3)
	Ni11-O107	2.016(9)	O107-Ni11-O124	94.3(4)
	Ni11-O123	2.003(9)	O107-Ni11-O125	94.4(4)
	Ni11-O124	2.019(9)	O123-Ni11-O93	91.8(3)
	Ni11-O125	2.052(8)	O123-Ni11-O107	86.6(4)
	Ni11-O126	2.012(9)	O123-Ni11-O124	172.7(4)
			O123-Ni11-O125	98.8(3)
			O123-Ni11-O126	93.7(4)
			O124-Ni11-O93	80.9(3)
			O124-Ni11-O125	88.4(3)
			O125-Ni11-O93	168.1(3)

			O126-Ni11-O93	80.2(3)
			O126-Ni11-O107	171.8(4)
			O126-Ni11-O124	84.4(4)
			O126-Ni11-O125	93.6(3)
**Ni12**	Ni12-O88	2.041(9)	O88-Ni12-O89	86.9(3)
	Ni12-O89	2.121(8)	O88-Ni12-O370	89.4(3)
	Ni12-O124	2.036(9)	O124-Ni12-O88	91.2(4)
	Ni12-O126	2.001(8)	O124-Ni12-O89	97.9(3)
	Ni12-O127	2.025(9)	O124-Ni12-O370	175.5(3)
	Ni12-O370	2.087(9)	O126-Ni12-O88	175.4(4)
			O126-Ni12-O89	93.0(3)
			O126-Ni12-O124	84.2(3)
			O126-Ni12-O127	80.8(3)
			O126-Ni12-O370	95.2(3)
			O127-Ni12-O88	99.1(3)
			O127-Ni12-O89	173.7(3)
			O127-Ni12-O124	80.5(3)
			O127-Ni12-O370	95.0(3)
			O370-Ni12-O89	86.6(3)

6. Powder X-ray Diffraction (PXRD)

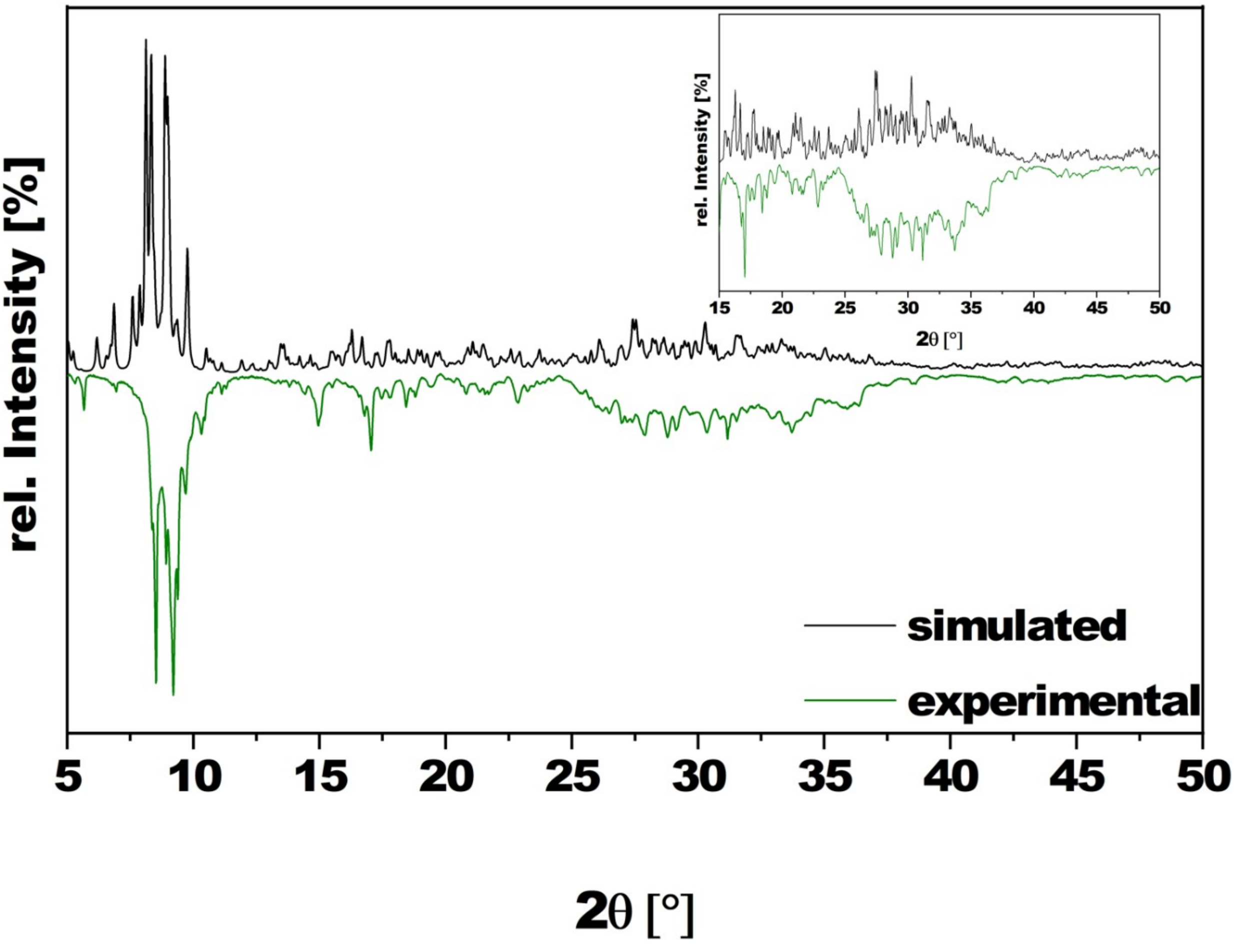

Figure S15. Comparison of the experimental and simulated PXRD pattern of $K_{11}Na_{10}$-**{Ni$_{12}$W$_{30}$}**. Note that differences between the simulated and the experimental PXRD patterns may be due to factors such as scanning speed, preferred orientation, and efflorescence of the crystals, which lose solvent molecules further leading to the collapse of the lattice.

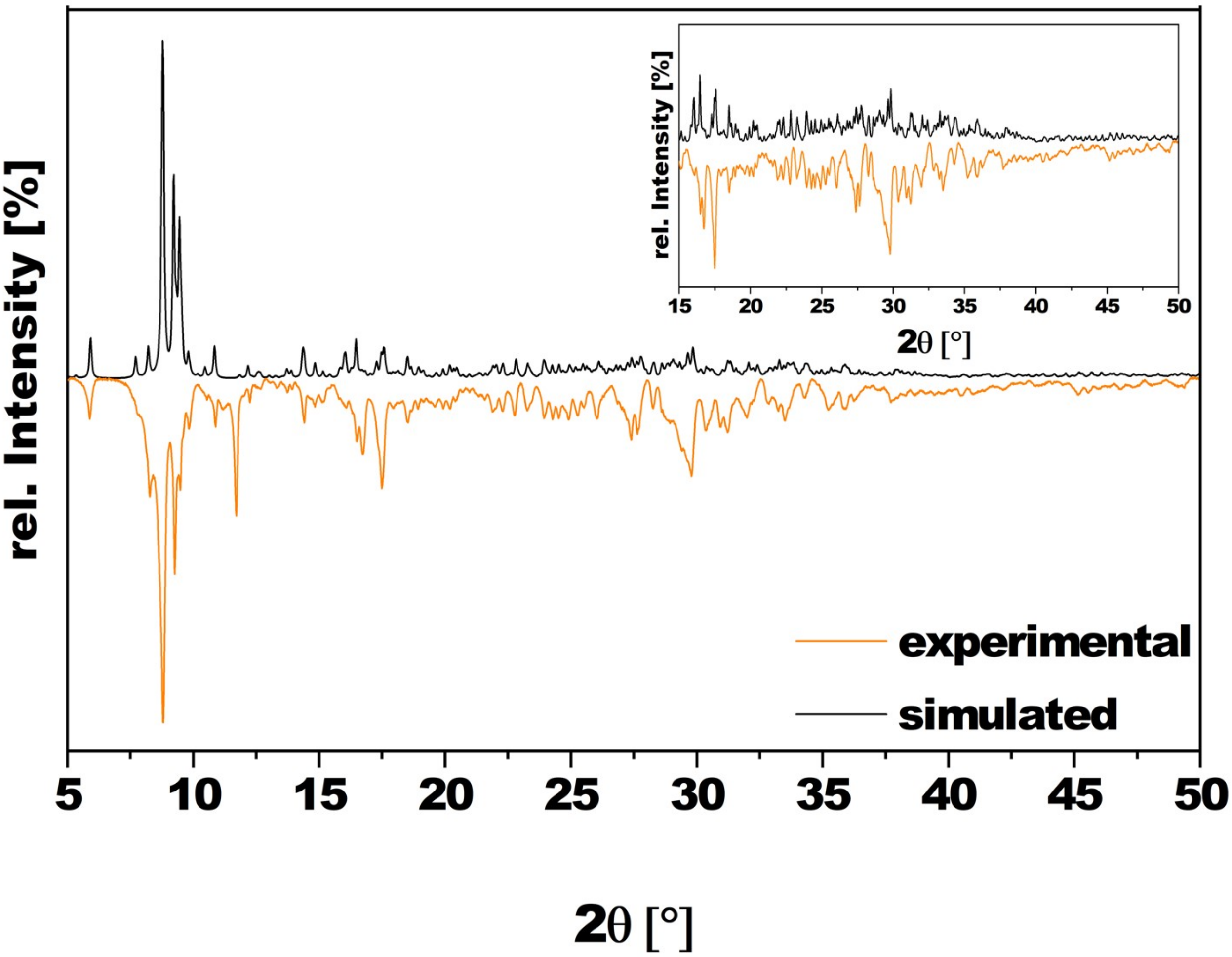

Figure S16. Comparison of the experimental and simulated PXRD pattern of $K_{14}Na_7$-**$\{Ni_{12}W_{27}\}$**. Note that differences between the simulated and the experimental PXRD patterns may be due to factors such as scanning speed, preferred orientation, and efflorescence of the crystals, which lose solvent molecules further leading to the collapse of the lattice.

7. Optical transition

7.1. Estimation of E_g using diffuse reflectance spectroscopy (DRS)

Diffuse reflectance spectroscopic (DRS) measurements in the range from 200 to 1000 nm were carried out on powdered samples of TBA-**{Ni$_4$W$_{18}$}**, TBA$_{13}$Na$_8$-**{Ni$_{12}$W$_{30}$}** and TBA$_{13}$Na$_8$-**{Ni$_{12}$W$_{27}$}** (**Figures S17 - S19**). The band gaps E_g of TBA-**{Ni$_4$W$_{18}$}**, TBA$_{13}$Na$_8$-**{Ni$_{12}$W$_{30}$}** and TBA$_{13}$Na$_8$-**{Ni$_{12}$W$_{27}$}** could be estimated by using the Tauc plot against the energy E [eV] derived from the DRS spectra by applying **Equations S1 and S2** and determining the intersection point between the energy axis and the line extrapolated from the linear portion of the absorption edge (**Figures S20 – S22**). The estimated optical transition values reveal the trend E_g (TBA$_{13}$Na$_8$-**{Ni$_{12}$W$_{27}$}**), E_g (TBA$_{13}$Na$_8$-**{Ni$_{12}$W$_{30}$}**) < E_g (TBA-**{Ni$_4$W$_{18}$}**) thereby further supporting the experimental and findings of the WRC studies. Additionally, the observed band gap trend E_g(TBA$_{13}$Na$_8$-**{Ni$_{12}$W$_{27}$}**) < E_g (TBA$_{13}$Na$_8$-**{Ni$_{12}$W$_{30}$}**) is in accordance with the ground spin states of **{Ni$_{12}$W$_{30}$}** (S = 0, low-spin) and **{Ni$_{12}$W$_{27}$}** (S = 6, high-spin) confirmed by theory (see chapter 10 “Magnetism”).

$$F(R_\infty) = \frac{K}{S} = \frac{(1 - R_\infty)^2}{2R_\infty}$$

, where K is the absorption coefficient,

S is the scattering factor and R is the reflectance [%]

obtained from the DRS spectrum (**Equation S1**)

$$(\propto h\nu)^2 = (F(R_\infty) \times E)^{1/2}$$ where $E = \frac{1239.7}{\lambda}$, $\lambda = \frac{1}{\nu}$, with λ being the corresponding x-axis value (nm) in the DRS spectrum (**Equation S2**)

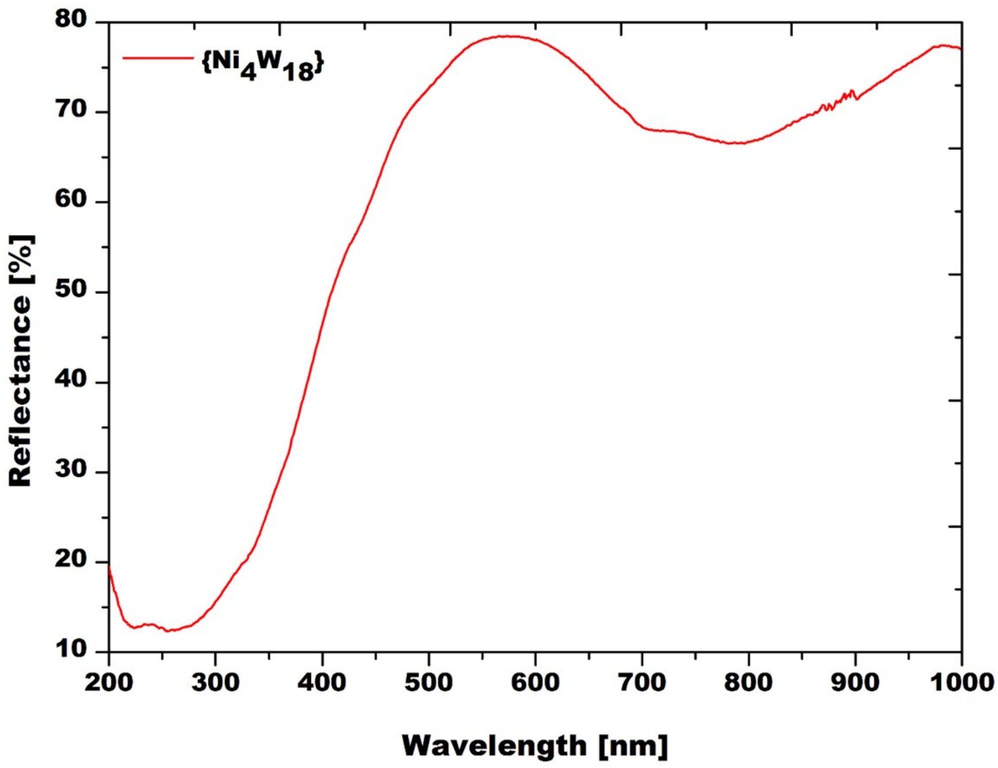

Figure S17. Diffuse reflectance spectrum of TBA-**{Ni_4W_{18}}**.

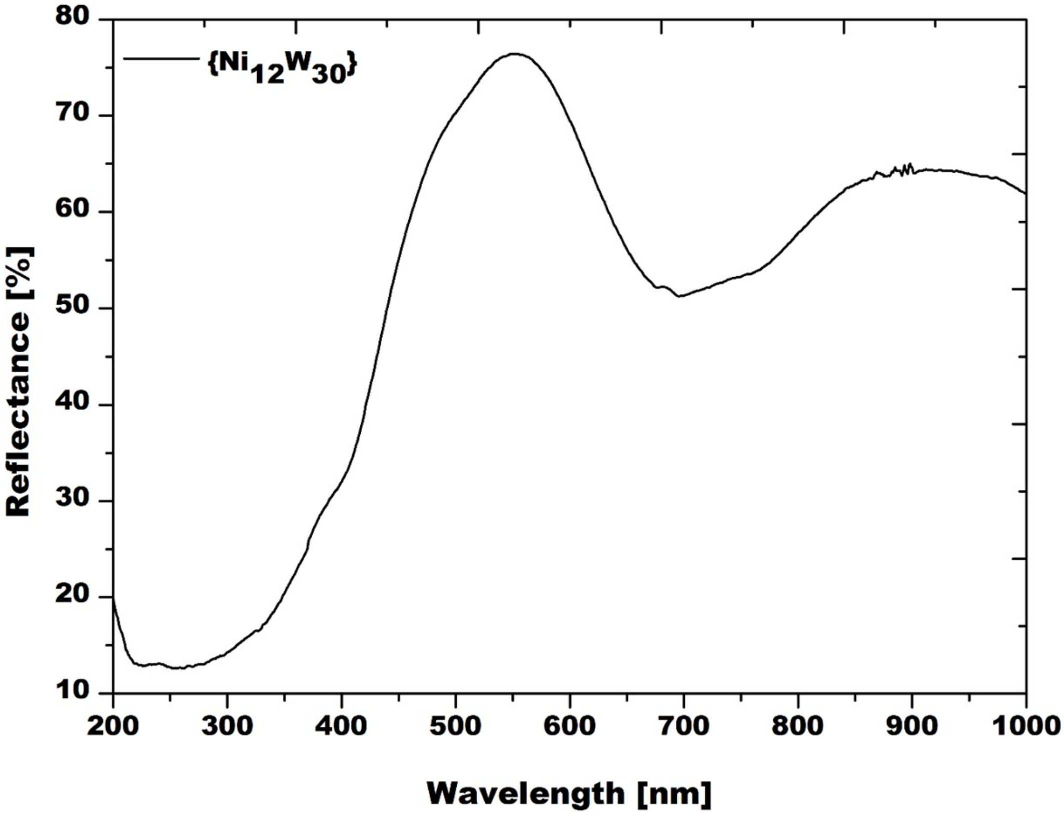

Figure S18. Diffuse reflectance spectrum of $TBA_{13}Na_8$-**{$Ni_{12}W_{30}$}**.

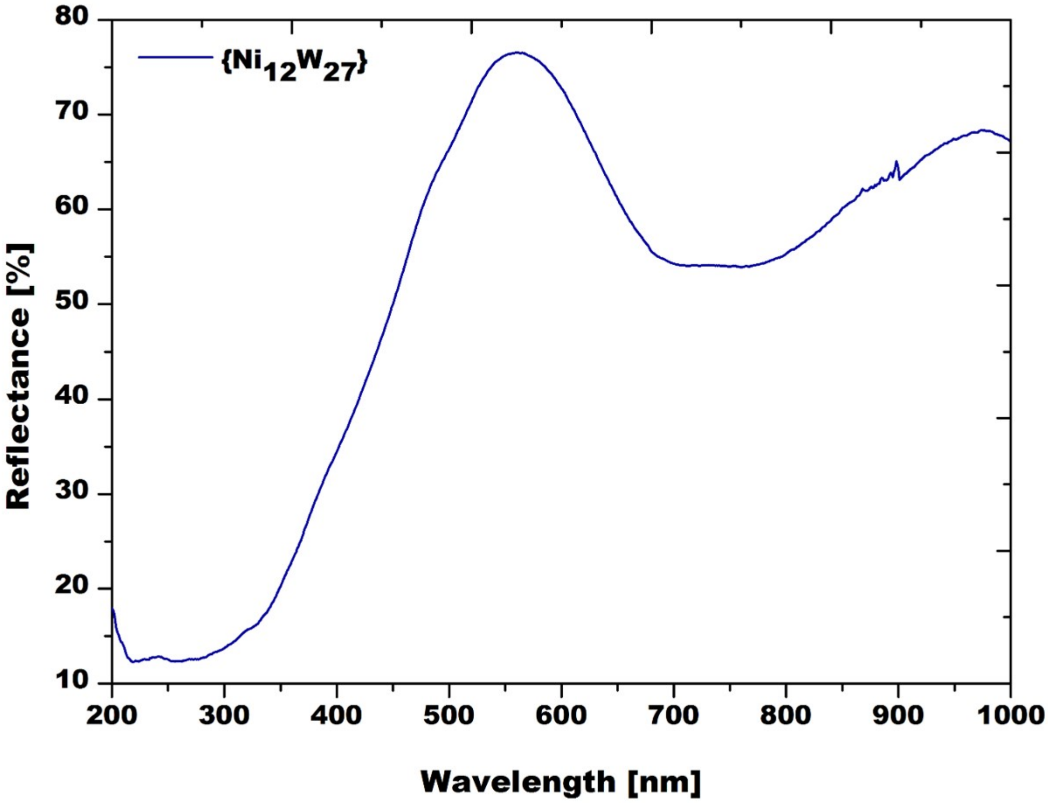

Figure S19. Diffuse reflectance spectrum of $TBA_{13}Na_8$-**{$Ni_{12}W_{27}$}**.

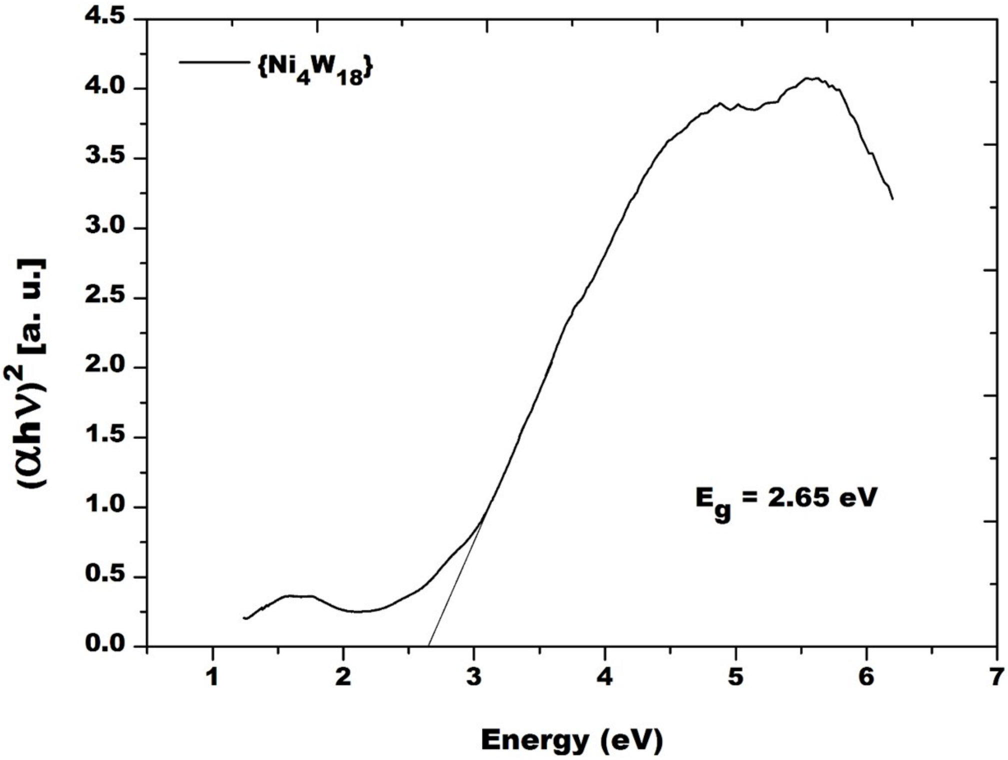

Figure S20. Tauc plot obtained from the diffuse reflectance spectrum of TBA-**{Ni_4W_{18}}** showing the HOMO-LUMO gap value E_g = 2.65 eV.

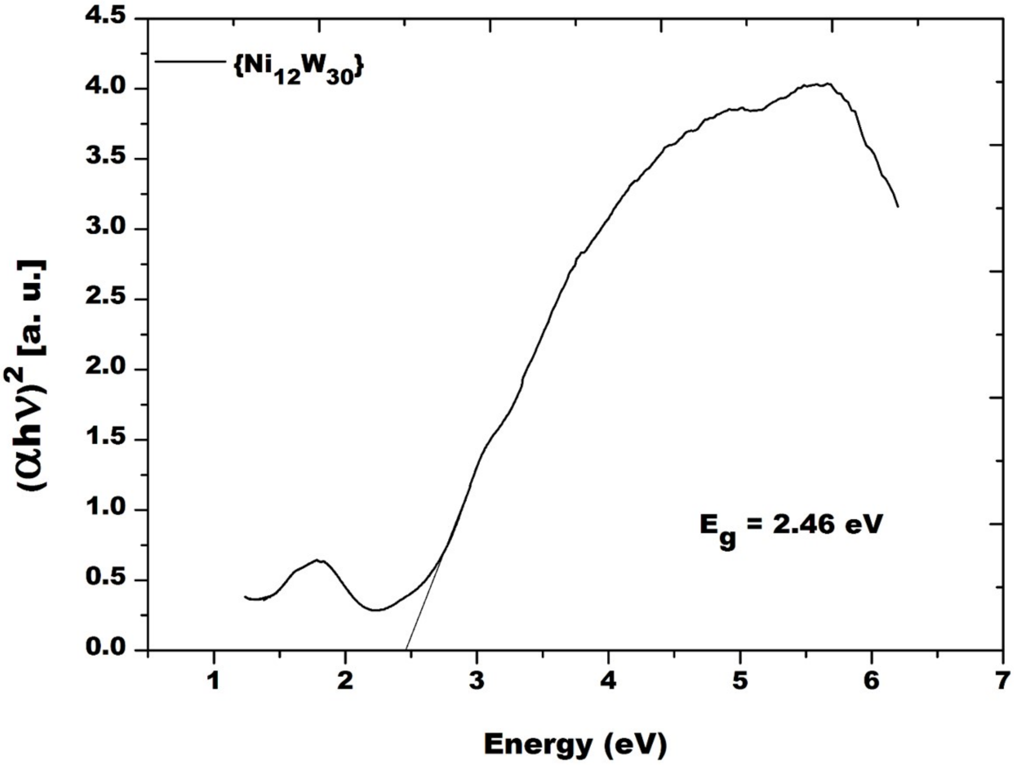

Figure S21. Tauc plot obtained from the diffuse reflectance spectrum of $TBA_{13}Na_8$-**{$Ni_{12}W_{30}$}** showing the HOMO-LUMO gap value E_g = 2.46 eV.

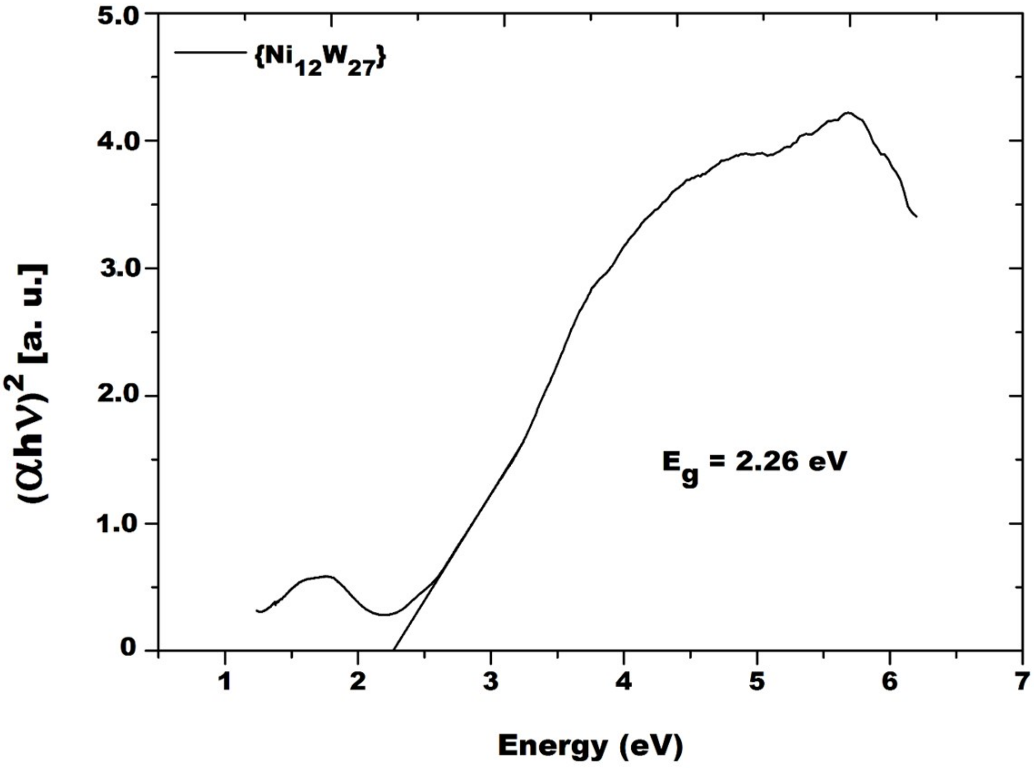

Figure S22. Tauc plot obtained from the diffuse reflectance spectrum of $TBA_{13}Na_8$-**{$Ni_{12}W_{27}$}** showing the HOMO-LUMO gap value E_g = 2.26 eV.

7.2. Estimation of band gap position using cyclic voltammetry (CV)

Considering that the LUMOs of POMs are formally a nonbonding combination of symmetry-adapted d_{xy} like orbitals centering on the metal (W) centers,[51] the LUMO levels can be estimated by finding out the applied onset potential for the first reduction of TBA-**{Ni_4W_{18}}**, $TBA_{13}Na_8$-**{$Ni_{12}W_{30}$}**, and $TBA_{13}Na_8$-**{$Ni_{12}W_{27}$}**,[52] allowing for the HOMOs to be calculated according to **Equation S3**:

$$E[LUMO(vs\ Vacuum)] = E[HOMO(vs\ Vacuum)] + E_g$$ (**Equation S3**)

Taking into account that the reduction potentials of POMs in the cyclic voltammograms are dependent on the applied test environment, all electrochemical experiments were carried out in CH_3CN/DMF (1/3) deaerated with Ar; glassy carbon working electrode, Pt wire auxiliary electrode, non-aqueous Ag^+/Ag reference electrode (0.1 M $TBAPF_6$ and 0.01 M $AgNO_3$) calibrated with the ferrocene/ferrocenium redox couple, T = 25°C, to eliminate the significant influence on the electrochemical response as far as possible. Based on the experimental setup, the ground and excited energy levels of **{Ni_4W_{18}}**, **{$Ni_{12}W_{30}$}**, and **{$Ni_{12}W_{27}$}** could be determined and calculated (**Figures S23 - 25**). By applying **Eq. 3**, estimated HOMO levels of -3.31 V (**{Ni_4W_{18}}**), -2.51 V (**{$Ni_{12}W_{27}$}**), and -2.58 V (**{$Ni_{12}W_{30}$}**) were obtained, thereby suggesting that the estimated HOMO levels of **{$Ni_{12}W_{27}$}** and **{$Ni_{12}W_{30}$}** lie higher in energy than the ones of **{Ni_4W_{18}}** which is in accordance with the observed activity trend of the HER experiments.

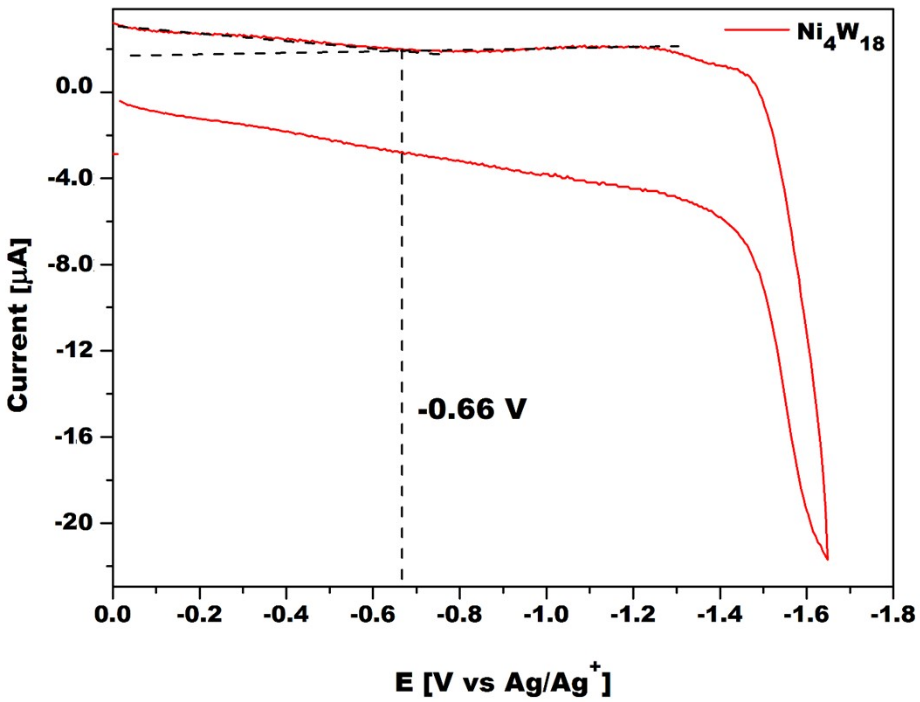

Figure S23. Cathodic range of the cyclic voltammogram of TBA-**{Ni_4W_{18}}** in ACN/DMF (1/3) at a 100 mV s^{-1} scan rate. The intersection point of the dotted line and the x-axis corresponds to the onset reduction potential of **{Ni_4W_{18}}**.

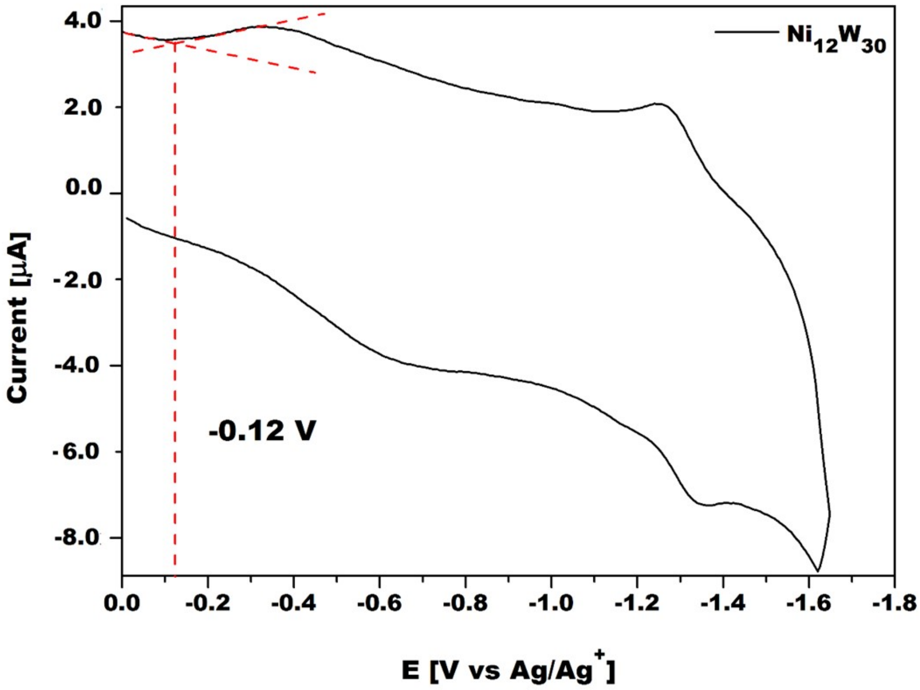

Figure S24. Cathodic range of the cyclic voltammogram of $TBA_{13}Na_8$-**{$Ni_{12}W_{30}$}** in ACN/DMF (1/3) at a 100 mV s^{-1} scan rate. The intersection point of the dotted line and the x-axis corresponds to the onset reduction potential of **{$Ni_{12}W_{30}$}**.

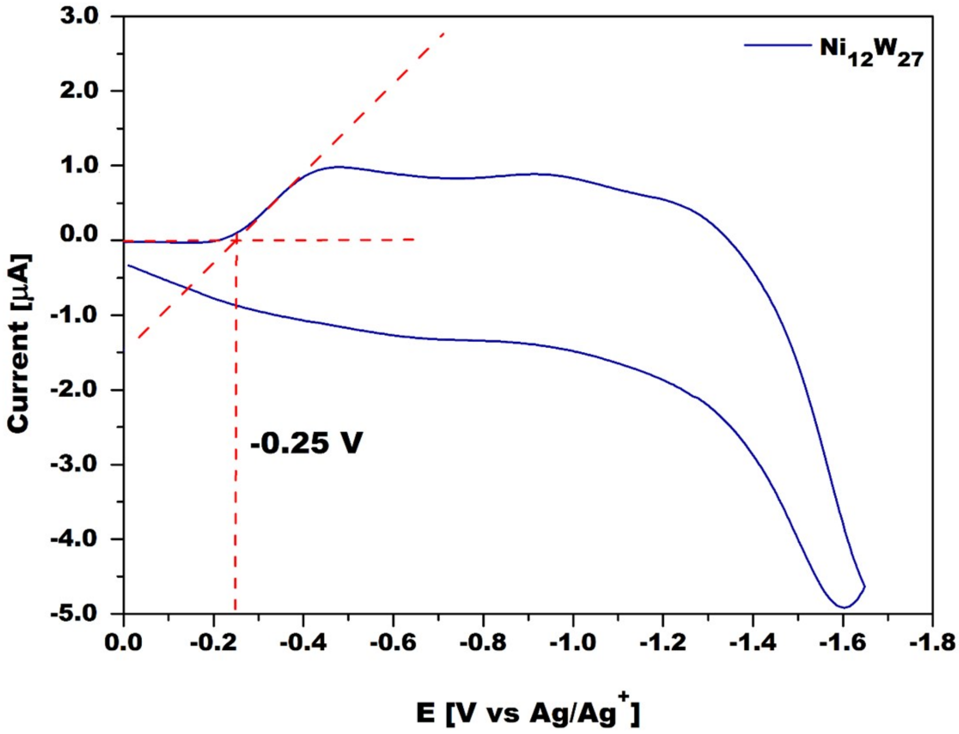

Figure S25. Cathodic range of the cyclic voltammogram of TBA$_{13}$Na$_8$-**{Ni$_{12}$W$_{27}$}** in ACN/DMF (1/3) at a 100 mV s^{-1} scan rate. The intersection point of the dotted line and the x-axis corresponds to the onset reduction potential of **{Ni$_{12}$W$_{27}$}**.

8. Cyclic voltammetry

Cyclic voltammograms of deaerated ACN/DMF (1/3) solvent mixtures containing 2.0 mM $TBA_{13}Na_8$-**$\{Ni_{12}W_{30}\}$** or $TBA_{13}Na_8$-**$\{Ni_{12}W_{27}\}$** were conducted thereby showing quasi-reversible, poorly resolved redox waves in the range of 0 to −1.65 V (**Figures S26, S28**), which are consistent with slow electron transfer rates likely due to high reorganization energies associated with Ni-PT based redox processes. A substantial overlap of the negative domain peaks corresponding to the reductions of W(VI) to W(V), W(V) to W(IV) and Ni(II) to Ni(I) renders them difficult to distinguish.[1b,53,54] The linear dependency of the peak current on the square root of the scan rates (R^2 ~ 0.998, **Figures S27, S29**) is consistent with diffusion-controlled interfacial redox processes.[1b] Addition of 3 M H_2O to a solution containing the corresponding Ni-PT leads to substantial current starting at -1.24 V, indicating onset of electrocatalysis (**Figure S30**).[1b] This observation is further supported by a control experiment in the absence of any Ni-PT catalyst under elsewise identical conditions showing a much lower current enhancement at more negative potential thus indicating that $TBA_{13}Na_8$-**$\{Ni_{12}W_{30}\}$** and $TBA_{13}Na_8$-**$\{Ni_{12}W_{27}\}$** are the electroactive species.

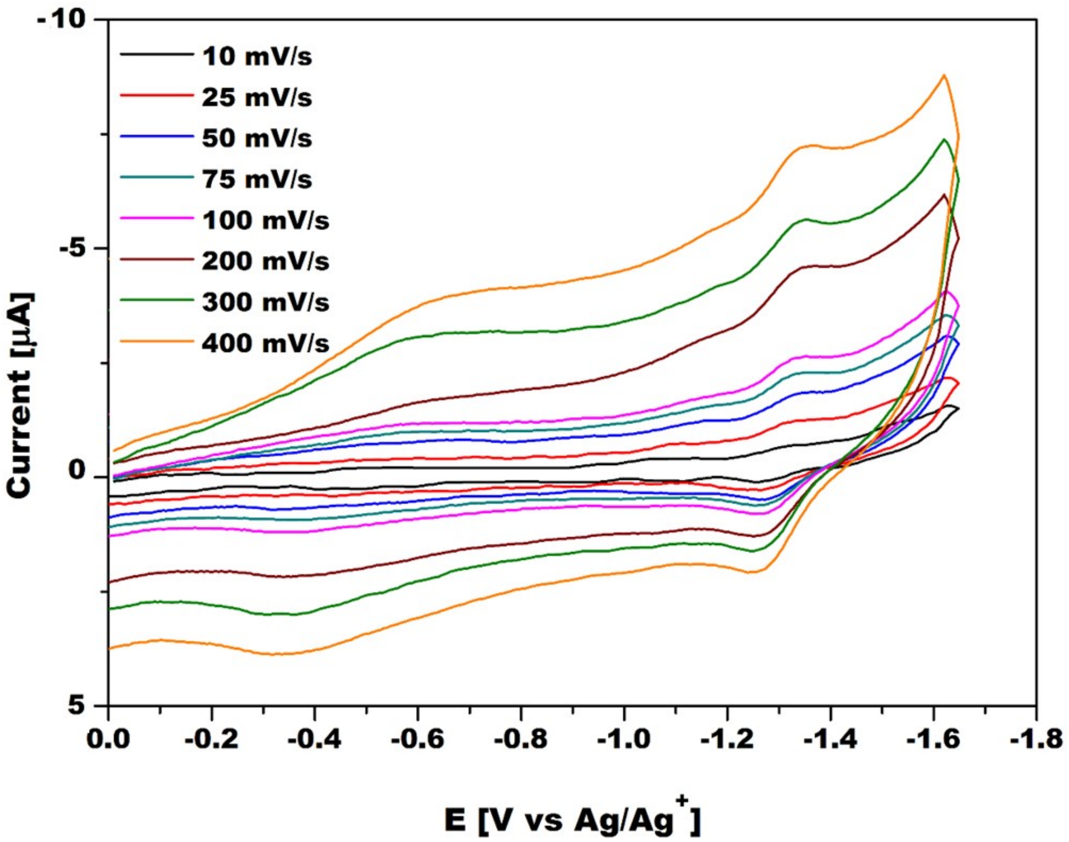

Figure S26. Cyclic voltammograms of 2.0 mM $TBA_{13}Na_8$-**$\{Ni_{12}W_{30}\}$** in deaerated ACN/DMF (1/3) at different scan rates in the range of 0 to −1.65 V.

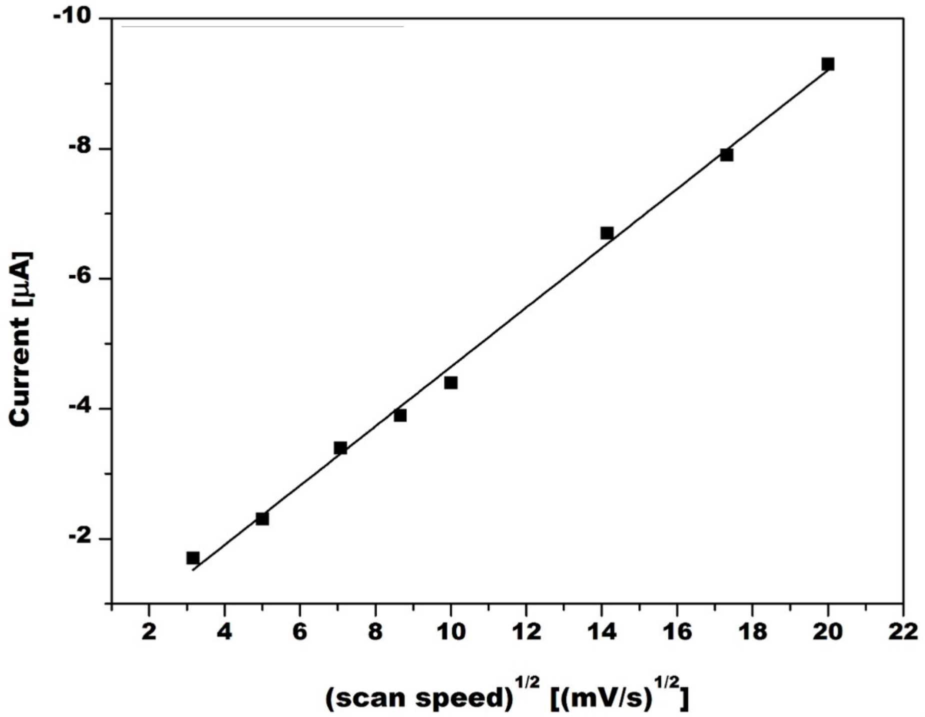

Figure S27. Plot of maximum anodic peak currents versus the square root of scan rates obtained from the cyclic voltammogram of $TBA_{13}Na_8$-**{$Ni_{12}W_{30}$}**.

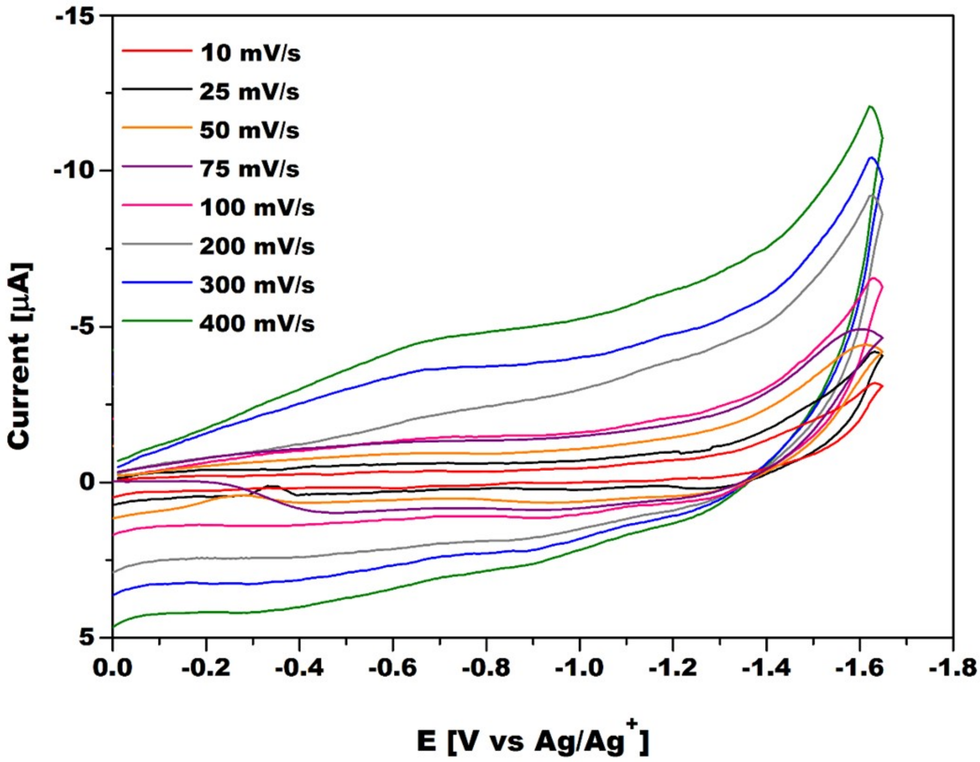

Figure S28. Cyclic voltammograms of 2.0 mM $TBA_{13}Na_8$-**{$Ni_{12}W_{27}$}** in deaerated ACN/DMF (1/3) at different scan rates in the range of 0 to −1.65 V.

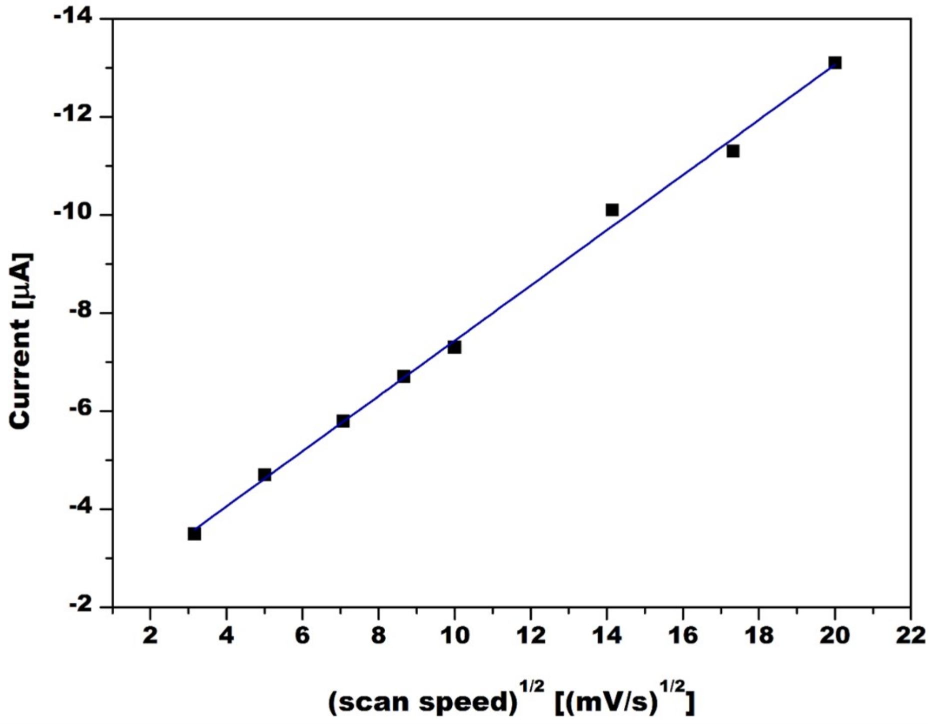

Figure S29. Plot of maximum anodic peak currents versus the square root of scan rates obtained from the cyclic voltammogram of $TBA_{13}Na_8$-**{$Ni_{12}W_{27}$}**.

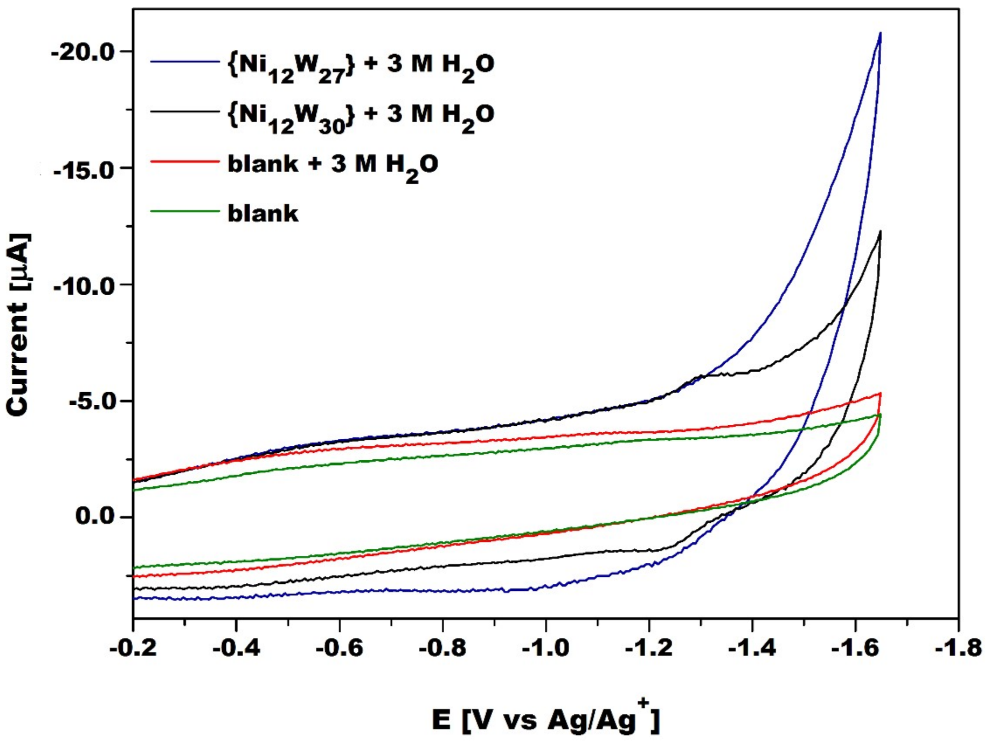

Figure S30. Successive cyclic voltammograms of 2 mM $TBA_{13}Na_8$-**{$Ni_{12}W_{27}$}** and $TBA_{13}Na_8$-**{$Ni_{12}W_{30}$}** in deaerated ACN/DMF (1/3), scan rate 100 mV/s in the presence of 3 M H_2O.

9. UV/vis spectroscopy

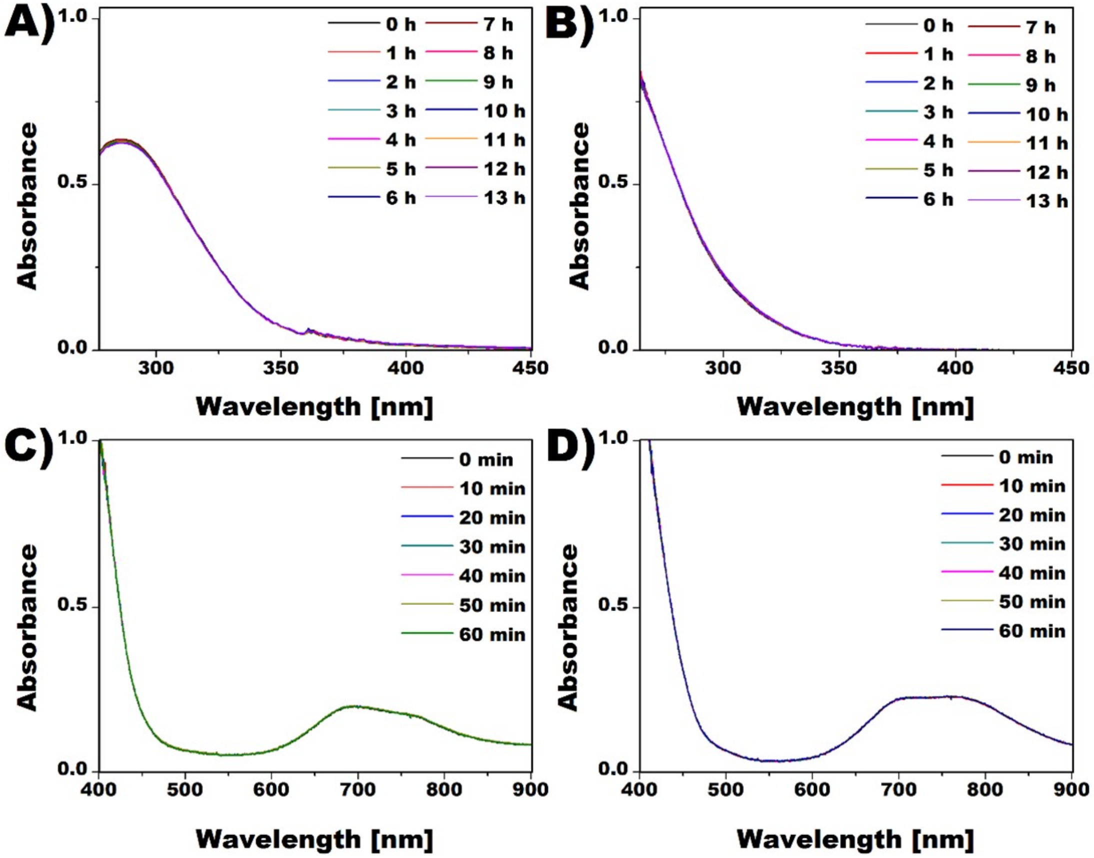

Figure S31. Time-dependent UV/Vis-spectra of **A)** $TBA_{13}Na_8$-**{$Ni_{12}W_{30}$}** (275 – 450 nm), **B)** $TBA_{13}Na_8$-**{$Ni_{12}W_{27}$}** (275 – 450 nm), **C)** $TBA_{13}Na_8$-**{$Ni_{12}W_{30}$}** (400 – 900 nm) and **D)** $TBA_{13}Na_8$-**{$Ni_{12}W_{27}$}** (400 – 900 nm) in DMF : CH_3CN : TEOA : H_2O = 33 : 11 : 2 : 4, v : v : v : v. The spectra show the unchanged O→W ligand−to−metal charge−transfer (LMCT) peak at **A)** 285 nm and **B)** 265 nm as well as the d-d transitions typical for octahedrally coordinated Ni^{II} metal centers at **C)** 688 nm and **D)** 700 nm, respectively, indicating pre-catalytic solution stability of the polyanions within the time range relevant to turnover conditions.

10. Magnetism

10.1. DFT - guided estimation of coupling constants (J_i values)

The OXO exchange paths (X = P or W) are essentially not effective in transmitting the magnetic interaction rendering the monoatomic pathway primary in the mediation of magnetic couplings through μ-OX bridging ligands (J_1-J_7) (**Figures S33**-**S35**). An accidental orthogonality situation in the μ-OX bridging ligands induces a dependence of nature and magnitude of the coupling on the Ni-O-Ni angle (α),[18] resulting in the interaction to become AF when a certain magic angle (*ca* 95°) is exceeded, which is supported by the simulations of $\chi_M T$ vs T for both compounds. Ferromagnetic couplings are more numerous than AF ones in **{Ni$_{12}$W$_{27}$}**, and even more pronounced in **{Ni$_{12}$W$_{30}$}** thereby explaining the increase in $\chi_M T$ for both **Ni-PTs** upon cooling as well as the initial decrease observed for **{Ni$_{12}$W$_{27}$}**. A singlet ground state occurs for **{Ni$_{12}$W$_{30}$}**, with very close triplet and quintet excited states in the Ni_4 subunits and excited states of even higher multiplicity for the Ni_{12} metal-oxo core, which accounts for the observed non-saturation of the magnetization (**Figure S36**). This scenario also occurs for the best-fit parameters: g_{Ni} = 2.24, $J_1 = J_2 = -4.4$ cm^{-1}, $J_3 = +6.6$ cm^{-1}, $J_4 = J_5 = +1.0$ cm^{-1}, $J_6 = +6.2$ cm^{-1}, and $J_7 = +9.2$ cm^{-1} (**Figure 2A**). Slight improvements are obtained with small changes of these parameters upon incorporating an axial zfs parameter ($|D|$ = 0.45 cm^{-1}) or dipolar intermolecular interactions through a mean-field approach ($\theta = -0.30$ K). Considering the crystal structure of **{Ni$_{12}$W$_{30}$}**, the first upgrading seems more adequate. Moreover, this D value represents a minimum amount since the approach of parallel local zfs tensors is like that suggested from CAS calculations ($D = -1.27$ cm^{-1}). The presence of competing interactions in the Ni_4 subunits of **{Ni$_{12}$W$_{27}$}** leads to a spin frustration topology, causing the emergence of a paramagnetic spin ground state (S = 2 or S = 1) for each Ni_4 subunit which is F coupled to reach an S = 6 ground state suggested by the experimental saturation value of the magnetization (**Figure S36**).
In contrast to **{Ni$_{12}$W$_{30}$}** where the bulky diamagnetic spacers separate the Ni_{12} clusters far away from each other in all directions, the crystal structure of **{Ni$_{12}$W$_{27}$}** displays **{Ni$_{12}$W$_{27}$}$_2$** supradimers with short Ni···Ni distances of 6.414 Å (**Ni9**···**Ni9**) and 6.535 Å (**Ni2**···**Ni5**) (**Figure S46**). This structural feature of **{Ni$_{12}$W$_{27}$}** would account for a non-negligible dipolar AF coupling between Ni_{12} units and the sharper downturn of $\chi_M T$, which cannot be reproduced through *zfs* effects exclusively. A similar situation occurs for the best-fit parameters: g_{Ni} = 2.20, $J_1 = J_2 = J_3 = +11.9$ cm^{-1}, $J_{4a} = J_{4b} = J_{5a} = J_{5b} = J_{6a} = J_{6b} = -38.0$ cm^{-1}, $J_{4c} = J_{5c} = J_{6c} = -9.6$ cm^{-1}, $J_7 = +9.4$ cm^{-1}, and $\theta = -1.8$ K (**Figures 2A**, **S37**), suggesting an S = 6 ground state with close excited states (S = 5 and S = 4) at 2.0 and 10.1 cm^{-1} (**Figure S36**).

Although the magnetic behaviors of these Ni_{12} complexes seem simple, their intricate molecular geometries, together with the possibility of a wide variety of magnetic couplings, make rigorous and reliable analysis a difficult task. Therefore, J_i values were first estimated from DFT calculations and used to analyze the experimental magnetic behavior. In both compounds, three Ni_4 groups linked together constitute the Ni_{12} molecular entities. The connections interlinking the Ni_4 units involve PO_4^{3-} and WO_4^{2-} diamagnetic bridging ligands that establish OXO (X = P and W) or even monoatomic O exchange pathways ($J_{7\text{-}13}$). **Figure S32** summarizes the topology of magnetic couplings found for $K_{11}Na_{10}$-**{Ni$_{12}$W$_{30}$}** and $K_{14}Na_7$-**{Ni$_{12}$W$_{27}$}**. A molecular description of these magnetic interactions and the most relevant geometric parameters that define them are detailed in **Tables S12** and **S13** and illustrated in **Figures S33** and **S34**. *A priori*, thirteen different J_i magnetic couplings grouped in six types ($J_{A\text{-}F}$) could describe the magnetic topology of these Ni_{12} systems, but they became thirty-nine because of the lack of symmetry between Ni_4 units (J_{ia}, J_{ib}, and J_{ic}). This feature is more notable in $K_{14}Na_7$-**{Ni$_{12}$W$_{27}$}**, where the encapsulated phosphate group acting as a bridging ligand between three Ni^{II} ions in each Ni_4 unit does so differently in one of them. Although the structural differences between these units are not very significant, $J_{ia\text{-}c}$ may be markedly different since the magnitude and nature of some of these interactions strongly depend on the Ni-O-Ni angle.

DFT calculations on the whole geometry and simplified Ni_{12} and Ni_2Zn_{10} models of $K_{11}Na_{10}$-**{Ni$_{12}$W$_{30}$}** show qualitatively equivalent results with the strongest ferromagnetic (F) and antiferromagnetic (AF) interactions being intensified in the models (**Table S12**). The small standard deviations of the J_i values

in the simplified Ni_{12} model indicate that the possible magnetic coupling between second neighbors, although present, is not relevant and can be ignored. The fact that the J_i values obtained on the Ni_2Zn_{10} model are like those for the simplified Ni_{12} one indicates that no significant electronic effects have been added when replacing Ni^{II} with Zn^{II} ions. However, some standard deviations derived from deleting any coupling between second neighbors prevent a correct estimation of the weakest $J_{8\text{-}13}$ interactions. Consequently, the discussion will be mainly based on the results of the simplified models, and only results from them will be provided for $K_{14}Na_7$-**{Ni$_{12}$W$_{27}$}**.

The weakest magnetic couplings usually occur when only OXO pathways (X = P or W) connect two Ni^{II} ions (J_8-J_{13}), evidencing the unsuitability of these connectors, in contrast to what occurs through carboxylate (OCO). Several factors would be responsible for this different efficiency: the shorter X-O bond length and the more favorable overlap with the oxygen atomic orbitals for X = C with respect to P or W. Finally, it deserves to be noted that the geometric arrangement of the metal ions relative to the OXO group has a great influence on the magnetic coupling. Thus, notable differences in the J_i values are expected in *syn-syn*, *syn-anti*, and *anti-anti* conformations (β angle, **Figure S35**), all of them observed in $K_{11}Na_{10}$-**{Ni$_{12}$W$_{30}$}** and $K_{14}Na_7$-**{Ni$_{12}$W$_{27}$}**. Furthermore, the Ni^{II} ion is located almost in the OXO plane (τ angle, **Figure S34**) or significantly out of it. When each metal ion adopts one of these conformations, but being different from each other, very F interactions are expected in many cases due to an accidental orthogonality.

When μ-hydroxo and carboxylate groups connect two metal ions, particularly Cu^{II}, the phenomenon of orbital counter-complementarity arises,[55] leading even to F couplings despite the fact that each bridging ligand separately favors AF interactions in their molecular conformation. However, phosphate or tungstate bridging ligands do not play this role here, as the magnetic coupling between two Ni^{II} ions is governed only by the hydroxo ligand. In this case, as with the di-μ-hydroxo, di-μ-alkoxo, di-μ-phenoxo or di-μ-azido homodinuclear copper(II) or nickel(II) complexes, there must be a magic angle at which a transition from F to AF will occur.[56] This value will depend on the bridging ligand, being different for μ-OH than for μ-OPO_3 or μ-OWO_3. Here, this magic value seems to be placed at *ca.* α = 95º (**Tables S12** and **S13**). Thus, even though only a single μ-OH pathway transmits the magnetic communication in the J_x coupling of $K_{14}Na_7$-**{Ni$_{12}$W$_{27}$}** because of the highly obtuse α angle (~127º), a strong AF coupling is mediated.

Other structural factors can modify the magnitude of the interaction, such as the butterfly distortion of the central unit Ni_2O_2 (δ) or the out-of-plane displacement of the hydrogen atom (μ-OH) or X (μ-OXO_3) from the Ni_2O_2 plane (γ) (**Figure S33**). However, these parameters do not change much in $K_{11}Na_{10}$-**{Ni$_{12}$W$_{30}$}** and $K_{14}Na_7$-**{Ni$_{12}$W$_{27}$}**. Besides, as observed in the past for other systems, they are usually strongly correlated to the α angle. For example, δ, γ, or even τ, defined as the out-of-plane of the Ni atom from the exchange pathway plane, increases as α decreases (**Table S12**).

Only average values of the most intense couplings obtained by the DFT study were considered in the analysis of the magnetic behaviors of $K_{11}Na_{10}$-**{Ni$_{12}$W$_{30}$}** and $K_{14}Na_7$-**{Ni$_{12}$W$_{27}$}** and the $J_{8\text{-}13}$ couplings were neglected. With this consideration, both compounds can be visualized as Ni_4 units ferromagnetically coupled to each other. Although the searching for best-fit parameters to reproduce the experimental $\chi_M T$ vs T curves is viable for a twelve coupled S = 1 local spin momenta under the framework of a Heisenberg Hamiltonian ($\hat{H} = -J\hat{S}_i\hat{S}_j$) applied on isotropic quantum spins, the required time is too long to be helpful. Suppose these spin momenta undergo *zfs* effects, as occurs for the Ni^{II} ion, the size of the generated matrix is too large to be stored in a conventional computer, and the time of a single simulation without applying highly advanced techniques together with simplifications would evolve from some tens of minutes to possibly quite a few months or years. This gets dramatically worse during a fitting. Thus, an approach based on effective Hamiltonians and developed in the past was applied to fit the spin Heisenberg models applied to recreate the observed behaviors of both compounds.[57] In this approach, some fragments (Ni_4) are exactly solved, and then considered as an effective spin (S_{eff}) with an effective g-factor (g_{eff}), which are temperature-dependent. In the whole system, these fragments are coupled to each other through an effective magnetic coupling (J_{eff}), related to the actual J_7 value. This J_{eff}, also temperature-dependent, is extracted from the energies and wavefunctions of the S states in fragments. The treatment of effective coupling between Ni_4 S_{eff} was done considering a classical spin approach through Langevin functions and the spin interaction topology

of $K_{11}Na_{10}$-**{$Ni_{12}W_{30}$}** and $K_{14}Na_7$-**{$Ni_{12}W_{27}$}** being a triangle.[58] This procedure fails at low temperature because the small size of the cycle allows a fast emergence of an autocorrelation error; that is, a spin momentum vastly is correlated with itself, requiring the more difficult task to develop an exact law. However, it was not needed since our simulation above 2.0 K moved all the time within the limit of applicability of the first technique for a triangular topology ($T/J_{eff}S_{eff}(S_{eff}+1) > 0.57$). The inclusion of a *zfs* for Ni^{II} ions makes the labor more difficult. However, a fast and relatively efficient technique entails considering these effects in the exact solution of the fragments and then adding them on the g_{eff}. Final verification is done for isotropic spin momenta by comparing the exact and approached simulations on the Ni_{12} system with the obtained best-fit parameters.

Applying this methodology, the experimental $\chi_M T$ vs T curve for $K_{11}Na_{10}$-**{$Ni_{12}W_{30}$}** was correctly simulated with the following parameters: g_{Ni} = 2.24, $J_1 = J_2 = -4.4$ cm^{-1}, $J_3 = +6.6$ cm^{-1}, $J_4 = J_5 = +1.0$ cm^{-1}, $J_6 = +6.2$ cm^{-1}, and $J_7 = +9.2$ cm^{-1}, and the agreement factor defined as $F = \sum(\chi T_{exp} - \chi T_{calc})^2 / \sum \chi T_{calc}^2$, equal to 5.2 x 10^{-5}. Small changes in these parameters and slight improvements in the agreement are observed upon incorporation of an axial *zfs* parameter (*D*) for Ni^{II} ions or dipolar intermolecular interactions through a mean-field approach (θ), applying the values $|D|$ = 0.45 cm^{-1} and θ = 0.30 K with the former representing a minimum value due to our model considering local zfs tensors are parallel. According to this result, the strongest $J_{1\text{-}3}$ and J_6 couplings impose a singlet ground state (**Figure S32**) in Ni_4 fragment and Ni_{12} unity, explaining the drop of $\chi_M T$ at low temperature. However, the non-zero J_4 and J_5 F couplings compete with the rest, moving paramagnetic excited states closer (**Figure S32**), a feature that accounts for the experimental dependence of the magnetization on the applied magnetic field and temperature.

The low symmetry of $K_{14}Na_7$-**{$Ni_{12}W_{27}$}** renders the analysis of magnetic behavior even more difficult. In such a case, there is a different thermal dependence of the effective factors, S_{eff} and J_{eff}, for each Ni_4 fragment, forcing the consideration of the classical heterospin model, which to the best of our knowledge has not yet been developed before. Following methodology based on Langevin function and the interaction model shown in **Figure S32**, the following analytical law has been deduced:

$$\chi T = \frac{2g_1^2S_1(S_1+1) + g_2^2(S_2+1)}{24}\left(3 + 2u_1 + 4\kappa u_2 + 4\kappa u_1u_2 + 2\kappa^2u_2^2 + 3\kappa^2u_1u_2^2\right)$$

(**Equation S4**),

where $u_i = Coth(x_i) - 1/x_i$ and $x_i = T/J_i\sqrt{S_a(S_a+1)}\sqrt{S_b(S_b+1)}$. Although not accurate at very low reduced temperatures ($T/J_{eff}S_{eff}(S_{eff}+1)$), it is sufficient for the purpose stated here. It should be noted that the Langevin function, that describes the spin correlation between two coupled centers, is normalized by a κ term that depends on their spin moments and *g*-factors, $\kappa = (M_1+M_2)^2 / (M_1^2+M_2^2) - 1$, M_i being g_iS_i. This term takes the unit value when the two centers are equivalent. The validity of this approximation has been verified against the exact simulation. However, the real spin coupling model is too intricate and has too many parameters to determine. For semiquantitative analysis of the thermal dependence of the magnetic susceptibility, the model has been simplified considering a single *g*-factor and reducing the number of active couplings to those stronger ($J_{1\text{-}7}$). Moreover, from the information provided by DFT study, some equivalences were imposed between parameters ($J_1 = J_2 = J_3$, $J_{4a} = J_{4b} = J_{5a} = J_{5b} = J_{6a} = J_{6b}$, and $J_{4c} = J_{5c} = J_{6c}$). The best-fit was achieved with the values g_{Ni} = 2.227, $J_1 = +11.9$ cm^{-1}, $J_{4a} = -38.0$ cm^{-1}, $J_{4c} = -9.6$ cm^{-1}, $J_7 = +9.4$ cm^{-1}, and $\theta = -1.8$ K. The agreement factor (F = 3.1 x 10^{-4}) was reasonable despite the high degree of simplification of the model. Note that **equation 4** is not an empirical law or a standard polynomial but a physical law for a classical spin approach. Therefore, **equation 4** is with physical meaning without introduction of any overparameterization in the fit process since it only presents a priori the J_i variable. S_{eff} and J_{eff}, which are both temperature dependent variables, are estimated from a previous treatment, hence not being a consequence of the equation above.

The predominant AF interaction in two Ni_4 units produces a slight decrease in $\chi_M T$ from room temperature. The rest of the F interactions are responsible for the significant increase in $\chi_M T$ < x K. The sharp fall in $\chi_M T$ at T < x K cannot be reproduced only by the presence of a *zfs* for Ni^{II} ions, which is undoubtedly acting. Only the dipolar or intermolecular interaction between two neighboring Ni_{12} units creating a supra-dimeric entity can reproduce this abrupt drop. According to these results, the ground state of each Ni_4 unit would be S = 1 or S = 2, depending on the Ni_4 fragment, intermediate spin arising from a spin frustration topology, with other excited states relatively far apart (**Figure S36**). Because these units are ferromagnetically coupled, the ground state of $K_{14}Na_7$-**{$Ni_{12}W_{27}$}** is an S = 6 without any close excited state with larger spin multiplicity. The closest excited state (S = 5) is placed at 2.0 cm^{-1} (**Figure S36**). This result agrees with the observed reduced magnetization curves projecting toward a saturation value corresponding to a ground state S = 6. The non-collapse of these curves is a consequence of the small gap with the first excited states, the weaker intramolecular couplings, but mainly of the widely reported *zfs* in octahedral nickel(II) complexes.[59]

The electronic effects in each Ni site are not equal (**Tables S10**, **S11**). While the surroundings of the vertex of each Ni_4 unity confirming the proposed Ni_3 core of **{$Ni_{12}W_{30}$}** (Ni1, Ni5 and Ni9, **Figure S32**) is composed of one OPO_3, two OWO_3 and three OH groups; four hydroxo, and two OXO_3 groups (P:W ratio for X = 1:1 or 2:0) occur in the rest of the Ni^{II} ions (**Figure S14B**), resulting in the presence of two groups of D values. Thus, in the latter, the coordination sphere can be defined as an elongated octahedron with four hydroxo ligands in the basal plane and OXO_3 of weaker ligand-field in more distant axial positions, leading to positive values of D. Meanwhile, Ni1, Ni2, and Ni3 sites exhibit a more compressed geometry but close to an ideal octahedron, so expecting a smaller value of D but of uncertain sign, which in our case is negative. There is a good correlation between these values and the distortion of the octahedral coordination sphere provided by shape measures (OC-6)[60], even if this parameter also embraces other geometrical factors that do not affect the axial zfs (**Figure S33**). From the calculated orientation of the local zfs tensors (**Table S14**), the D value for an S ground state would be $D = -15.2/S^2$ with E/D = 0.14. In the fit process in polynuclear complexes, it is usual to consider an average local D value in polynuclear complexes, equivalent to arranging all local tensors parallel. According to this approach and previous calculations, the average local D value for each Ni^{II} ion would be $D = -1.27$ cm^{-1}.[61] This value may seem small, but in an ideal tetrahedral Ni_4 arrangement, a null D for any resulting state with D_i therefore being null for the Ni_{12} entities, as well, should be expected.
Simulations of the magnetic behavior were performed using an approach based on an effective Hamiltonian, where effective spin momenta (S_{eff}) of each Ni_4 unity and the effective coupling (J_{eff}) between them are temperature-dependent and obtained from the exact solution of Heisenberg Ni_4 systems. Accordingly, the thermal effect of the zfs of Ni^{II} ions is included from an effective g-factor (g_{eff}) for each Ni_4 fragment. To simplify, the coupling between these fragments was obtained from a classical spin approach, developing $\chi_M T$ as a combination of Langevin functions.

In the less symmetrical **{$Ni_{12}W_{27}$}**, a classical spin law for a heterospin triangle was deduced. Considering the short self-connecting pathway when cycling a triangle, the range of applicability is more limited ($T/(J_{eff}\,S_{eff}\,(S_{eff}+1)) > 0.57$) as compared to a 1D system.[58] However, $J_{eff}\,S_{eff}\,(S_{eff}+1)$ decreases with temperature, ensuring the model's suitability for temperatures up to 2.0 K. Thus, a final verification for isotropic spin momenta shows a complete agreement between exact and approached simulations on the Ni_{12} system with the obtained best-fit parameters.

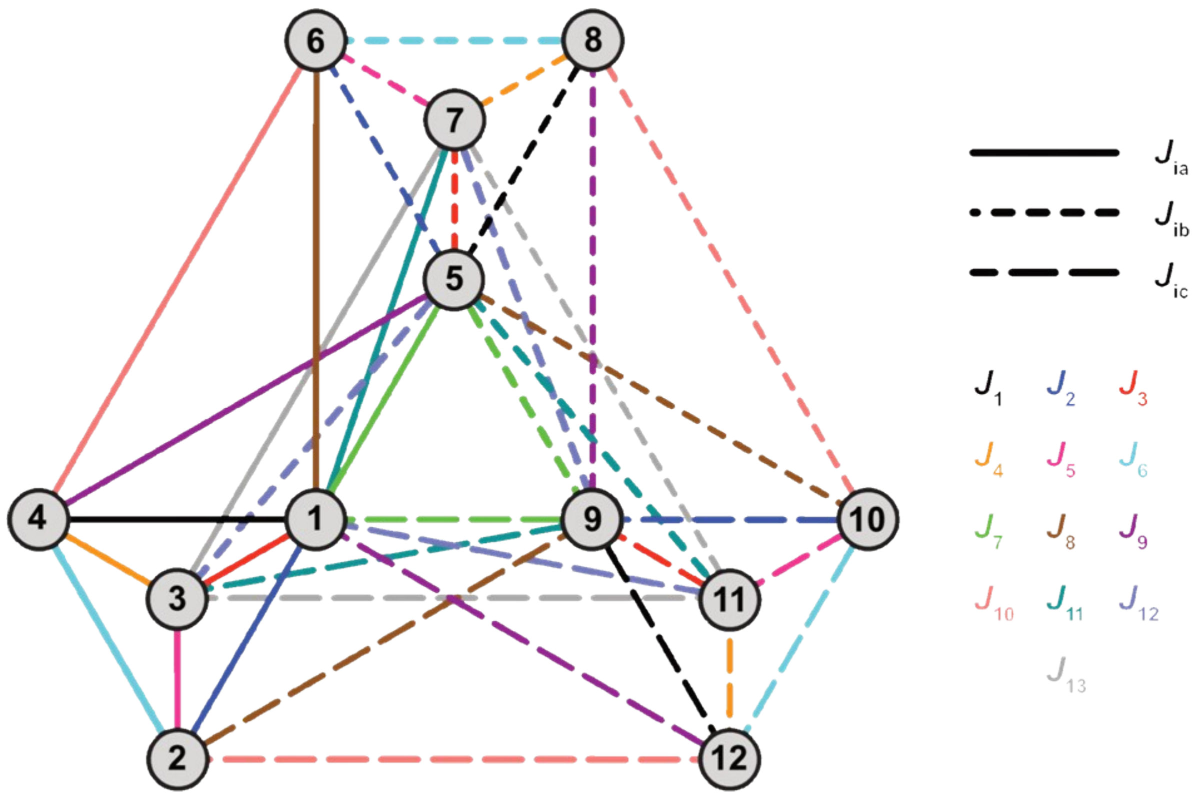

Figure S32. Topology of the spin coupling in **{$Ni_{12}W_{30}$}** and **{$Ni_{12}W_{27}$}**.

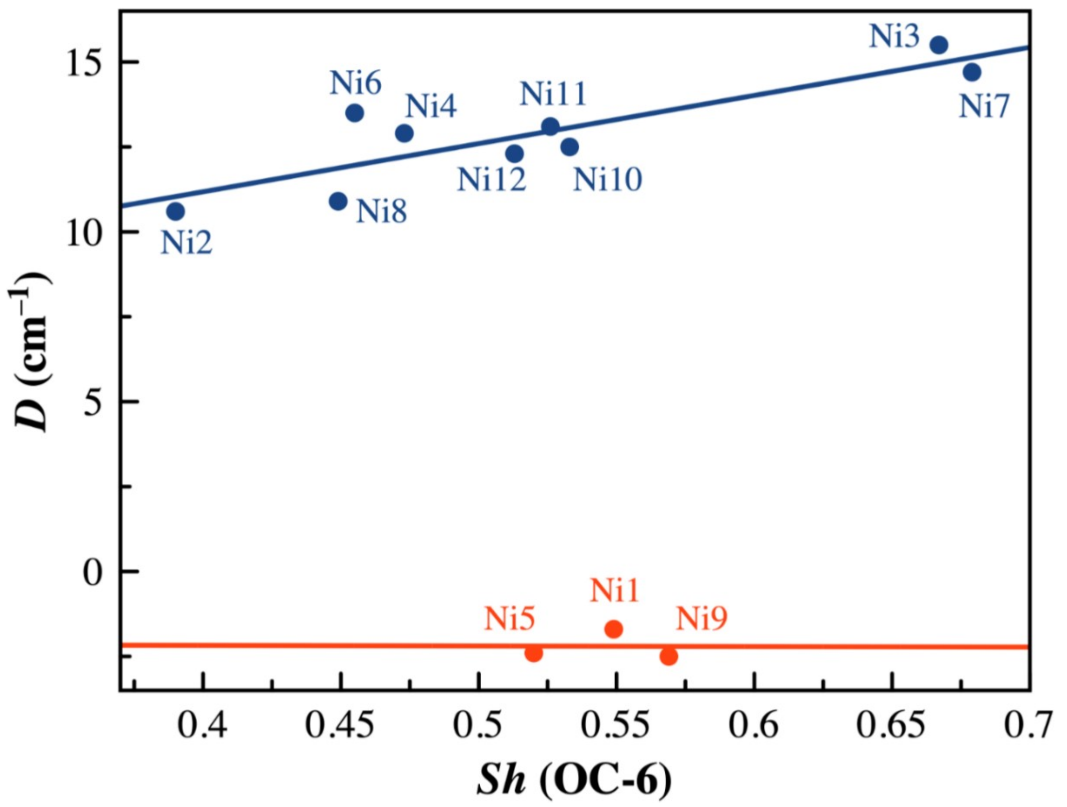

Figure S33. Geometric dependency of the calculated axial zfs shown as *D* vs shape measures (*Sh*) plots for **{$Ni_{12}W_{30}$}** (see **Table S14**). Labels are used to identify the data for each Ni site.

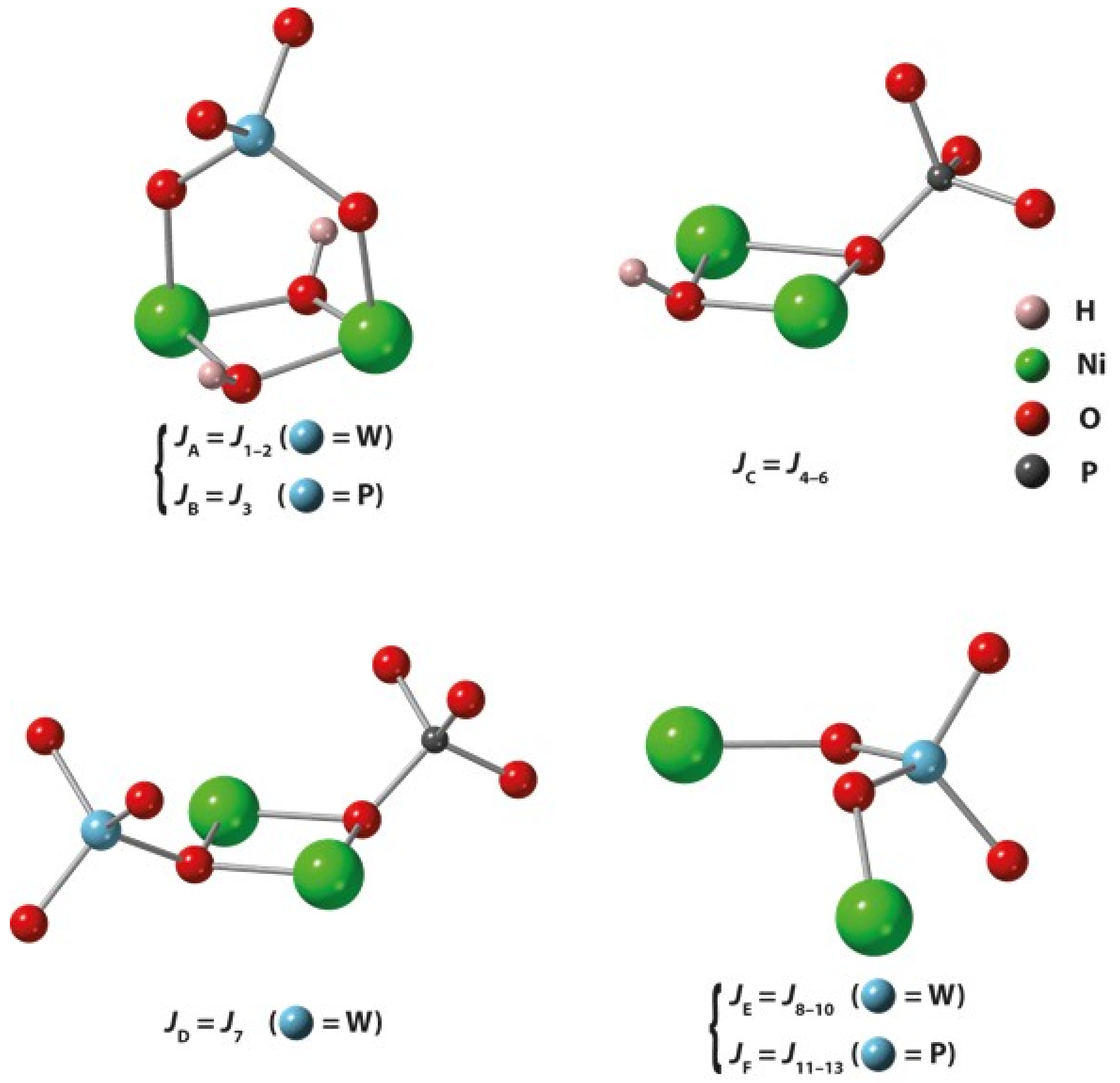

Figure S34. Magnetic coupling pathways referred in **Figure S32**, **Tables S12** and **S13**.

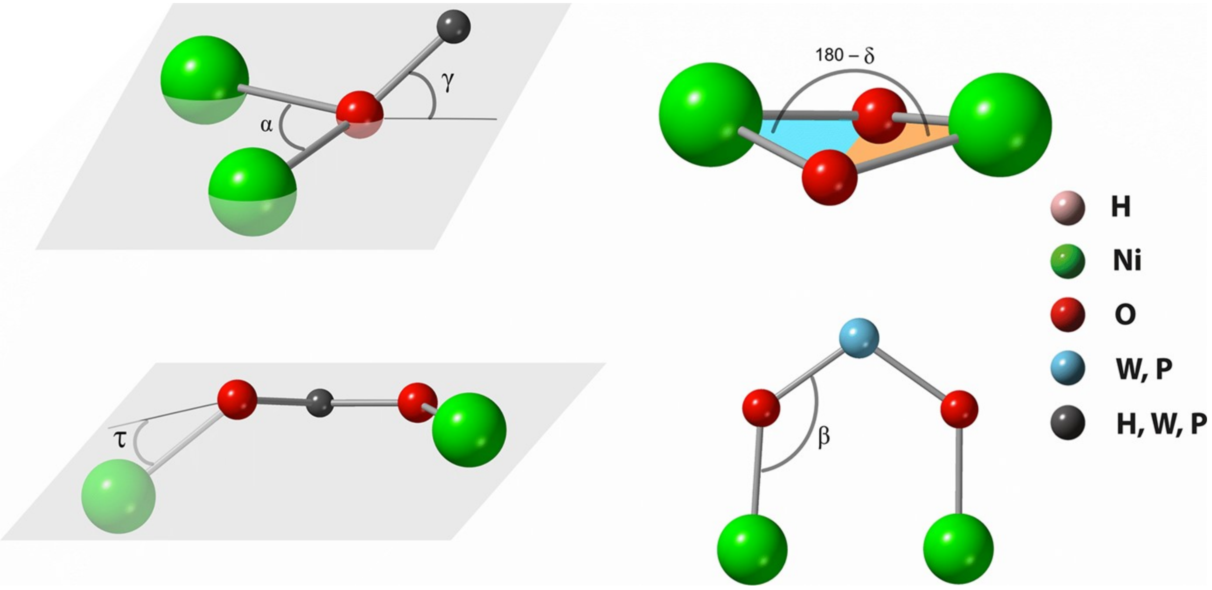

Figure S35. Most important structural parameters tunning the magnetic couplings in **{Ni$_{12}$W$_{30}$}** and **{Ni$_{12}$W$_{27}$}**.

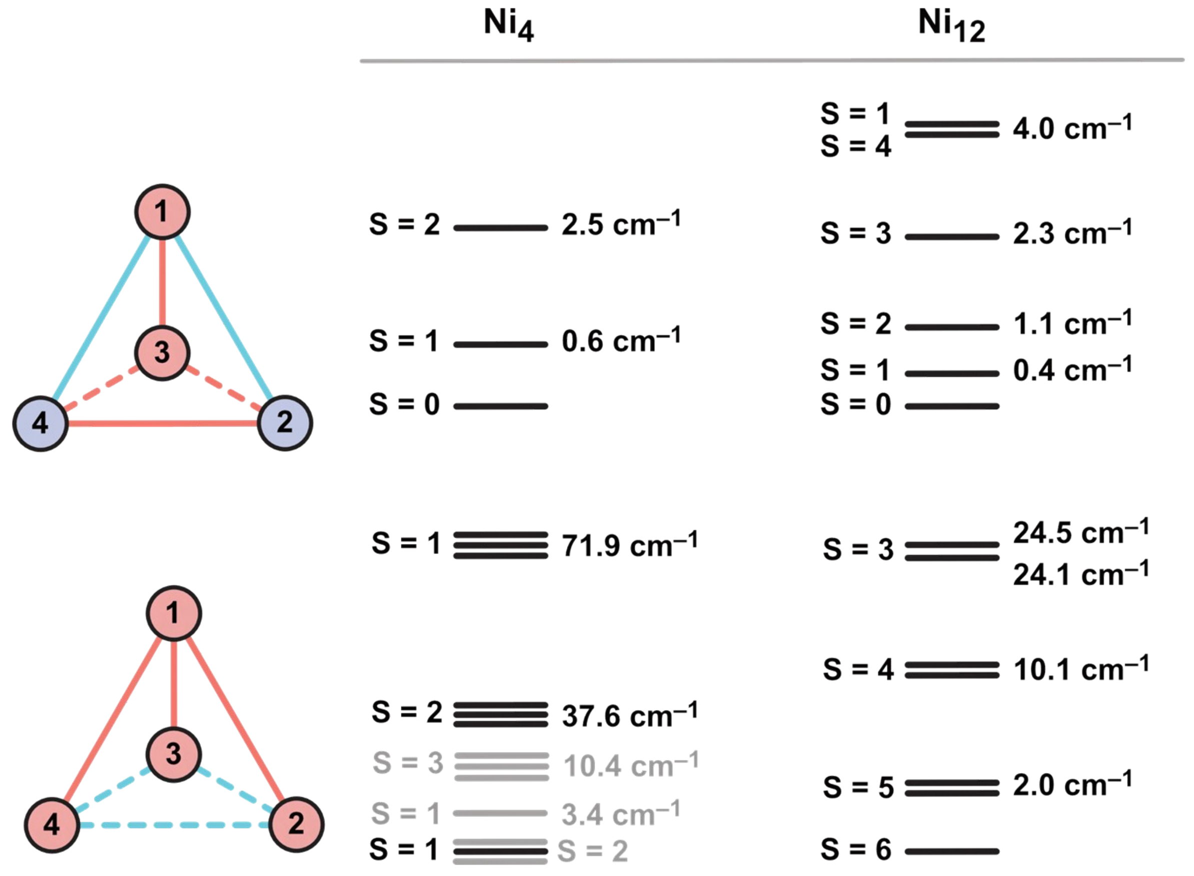

Figure S36. Spin topologies (left) of Ni$_4$ fragments and ordering of low-lying *S* states of Ni$_4$ fragments and Ni$_{12}$ unit (right) for **{Ni$_{12}$W$_{30}$}** (top) and **{Ni$_{12}$W$_{27}$}** (bottom). Red and blue spheres denote positive and negative spin momenta. Red and blue lines denote F and AF couplings. Solid lines are used for the strongest couplings. Dotted lines show the weakest competing couplings that can lead to a spin frustration scenario. For **{Ni$_{12}$W$_{27}$}**, black and grey labels are used for Ni$_4$ fragments appearing twice or once in the Ni$_{12}$ unit.

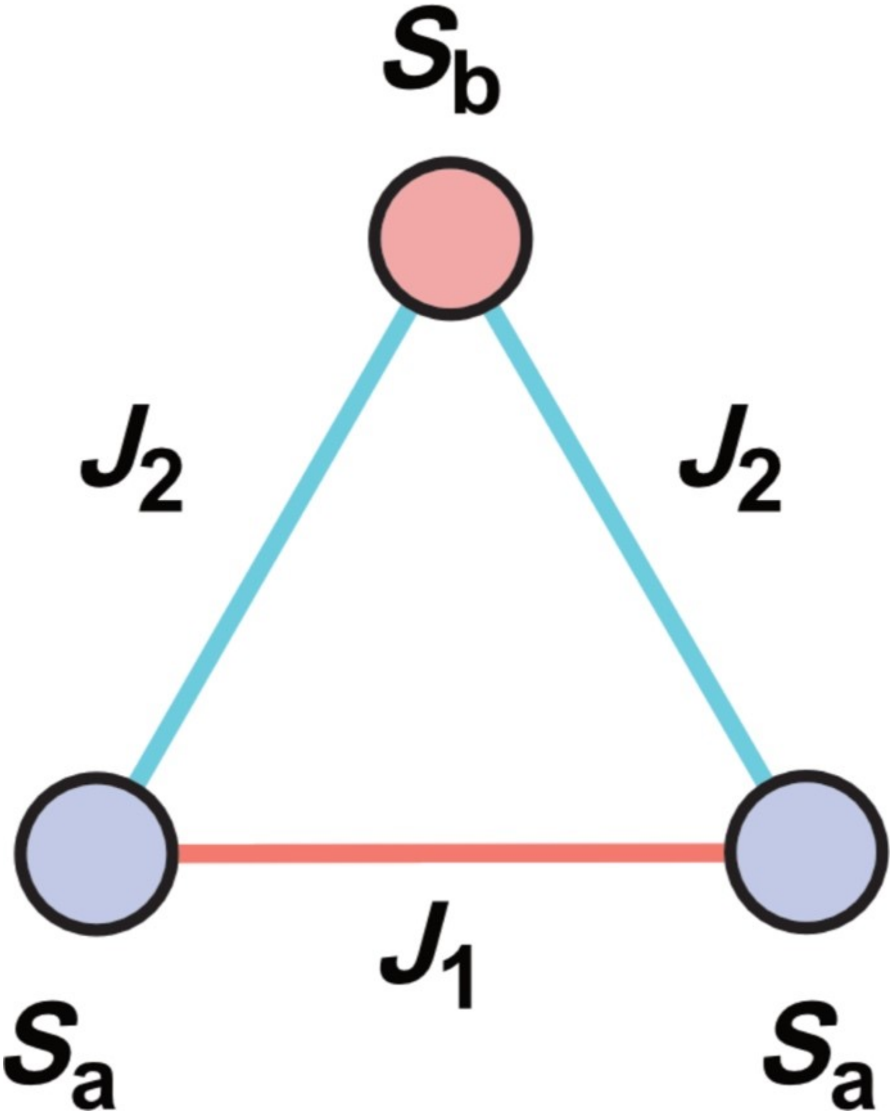

Figure S37. Spin topology for the coupling of the S_{eff} momenta corresponding to each Ni_4 unit in **{$Ni_{12}W_{27}$}**. Different colors are used to note different magnetic nature of each fragment and different magnetic interaction between them.

Table S12. Relevant Structural Parameters Involved in the Different Magnetic Exchange Pathways of **{$Ni_{12}W_{30}$}** along with the *J* values estimated from DFT Calculations.

	Sites [a]	Pathways	dNi–Ni[b]	α [c]	δ [c]	γ [c]	β [c]	τ [c]	*J*[d]	J_{model}[e]	J_{model}[f]
J_{1a}	1,4	μ_3-OH/μ_3-OH/μ-O_2WO_2	3.050	98.2/97.5	13.0	145.1/143.2	124.9/129.0	2.8/1.7		–13.8(5)	–15.5
J_{1b}	5,8	μ_3-OH/μ_3-OH/μ-O_2WO_2	3.054	98.0/98.0	12.3	145.1/143.2	124.3/129.8	2.0/4.1	–2.6(2)	–14.5(6)	–15.4
J_{1c}	9,12	μ_3-OH/μ_3-OH/μ-O_2WO_2	3.043	98.5/97.5	13.0	146.1/142.4	125.3/127.5	3.3/10.3		–12.1(6)	–13.1
J_2	1,2	μ_3-OH/μ_3-OH/μ-O_2WO_2	3.027	97.5/96.9	13.3	149.6/142.7	124.5/129.7	6.3/6.2		–14.2(7)	–15.3
J_{2b}	5,6	μ_3-OH/μ_3-OH/μ-O_2WO_2	3.033	97.2/97.8	13.4	147.5/142.8	126.0/128.3	4.8/3.1	–1.8(2)	–12.8(6)	–14.0
J_{2c}	9,10	μ_3-OH/μ_3-OH/μ-O_2WO_2	3.053	98.9/98.1	12.6	146.9/142.7	124.2/128.9	4.8/4.6		–14.4(5)	–15.1
J_3	1,3	μ_3-OH/μ_3-OH/μ-O_2PO_2	2.954	94.0/94.7	21.3	147.5/147.3	124.9/134.7	6.6/13.1		+21.4(5)	+22.1
J_{3b}	5,7	μ_3-OH/μ_3-OH/μ-O_2PO_2	2.962	93.4/94.0	20.9	147.8/146.9	123.8/136.4	6.4/12.0	+11.9(5)	+19.4(5)	+20.2
J_{3c}	9,11	μ_3-OH/μ_3-OH/μ-O_2PO_2	2.935	94.0/94.1	21.5	150.5/145.9	125.2/135.2	6.5/10.1		+21.5(5)	+21.7
J_4	3,4	μ_3-OH/μ_3-OPO_3	3.230	107.2/91.7	4.4	140.0/145.9				+0.2(5)	+0.70
J_{4b}	7,8	μ_3-OH/μ_3-OPO_3	3.211	106.0/91.9	4.4	139.3/145.6			–1.9(4)	+0.8(6)	+1.8
J_{4c}	11,12	μ_3-OH/μ_3-OPO_3	3.207	106.4/91.8	3.8	135.2/144.5				+2.4(6)	+2.7
J_5	2,3	μ_3-OH/μ_3-OPO_3	3.214	106.7/91.9	3.6	134.5/144.7				+2.2(5)	+2.8
J_{5b}	6,7	μ_3-OH/μ_3-OPO_3	3.222	106.2/91.9	3.7	139.2/144.6			–1.3(4)	+1.9(5)	+2.4
J_{5c}	10,11	μ_3-OH/μ_3-OPO_3	3.221	106.4/92.0	4.5	139.6/145.6				+2.1(5)	+2.2
J_6	2,4	μ_3-OH/μ_3-OPO_3	3.152	102.9/91.6	8.2	146.4/148.3				+5.8(6)	+6.5
J_{6b}	6,8	μ_3-OH/μ_3-OPO_3	3.155	103.2/92.2	8.5	145.7/148.3			+5.3(3)	+6.7(6)	+7.4
J_{6c}	10,12	μ_3-OH/μ_3-OPO_3	3.157	103.2/91.4	9.6	146.5/149.6				+6.6(6)	+7.5
J_7	1,5	μ_3-OPO_3/μ-OWO_3	3.044	92.0/95.1	0.4	144.20/149.12				+9.9(6)	+10.2
J_{7b}	5,9	μ_3-OPO_3/μ-OWO_3	3.041	89.3/94.2	0.2	144.46/146.09			+9.4(3)	+8.9(7)	+9.4
J_{7c}	1,9	μ_3-OPO_3/μ-OWO_3	3.017	91.4/93.2	2.8	145.26/147.93				+8.3(7)	+8.3
J_8	1,6	μ-O_2WO_2	5.214				235.1/128.3	43.0/3.1		+0.0(9)	–0.44
J_{8b}	5,10	μ-O_2WO_2	5.242				235.7/128.9	46.2/4.6	–5.5(4)	–0.9(5)	+0.47
J_{8c}	2,9	μ-O_2WO_2	5.166				234.7/129.7	46.5/6.2		–0.7(6)	+0.70
J_9	4,5	μ-O_2WO_2	5.272				234.0/129.0	40.6/1.7		–0.4(8)	–0.75
J_{9b}	9,8	μ-O_2WO_2	5.271				235.8/129.8	41.1/4.1	–6.2(4)	–2.1(6)	–1.2
J_{9c}	1,12	μ-O_2WO_2	5.212				235.5/127.5	39.9/10.3		–3.6(6)	–1.4
J_{10}	4,6	μ-O_2WO_2	5.993				231.0/231.7	43.6/40.6		–2.2(8)	–0.37
J_{10b}	8,10	μ-O_2WO_2	6.023				230.2/231.1	43.7/38.6	–1.1(7)	–0.1(6)	–0.42
J_{10c}	2,12	μ-O_2WO_2	5.872				230.3/232.5	36.9/49.3		–0.6(2)	–1.0
J_{11}	1,7	μ-O_2PO_2	5.169				235.1/136.4	39.7/12.0		+0.6(2)	+0.69

J_{11b}	5,11	μ-O_2PO_2	5.082	236.2/135.2	40.5/10.1	+5.1(2)	+0.2(11)	+0.70
J_{11c}	9,3	μ-O_2PO_2	5.091	234.8/134.7	41.1/13.1		+0.8(8)	+0.69
J_{12}	5,3	μ-O_2PO_2	5.086	236.2/134.7	49.9/13.1		+0.6(17)	+0.48
J_{12b}	9,7	μ-O_2PO_2	5.162	234.8/136.4	48.8/12.0	+5.1(2)	+0.8(9)	+0.52
J_{12c}	1,11	μ-O_2PO_2	5.022	235.1/135.2	50.3/10.1		+1.2(8)	+0.51
J_{13}	3,7	μ-O_2PO_2	5.763	225.3/223.6	27.8/42.3		–3.8(15)	–4.3
J_{13b}	7,11	μ-O_2PO_2	5.770	223.6/224.8	27.7/42.6	–6.6(3)	–4.5(12)	–4.7
J_{13c}	3,11	μ-O_2PO_2	5.601	235.1/128.3	43.0/3.1		–5.4(9)	–0.44

[a]Labeling in **Figure S34**. [b]Intermetalic distance in angstroms. [c]Angles (in degrees) described in **Figure S35**. [d]Average values in cm^{-1} calculated on the full experimental geometry. Standard deviations in parentheses. [e]Values (in cm^{-1}) obtained on the simplified Ni_{12} model. Standard deviations in parentheses. [f]Values (in cm^{-1}) obtained on the simplified Ni_2Zn_{10} model.

Table S13. Relevant Structural Parameters Involved in the Different Magnetic Exchange Pathways of **{$Ni_{12}W_{27}$}** along with the *J* values estimated from DFT Calculations.

	**Sites** [a]	**Pathways**	**dNi–Ni**[b]	α [c]	δ [c]	γ [c]	β [c]	τ [c]	J_{model} [d]	J_{model} [e]
J_{1a}	1,4	μ_3-OH/μ_3-OH/μ-O_2PO_2H	3.004	94.9/95.5	31.03	138.9/140.3	123.3/126.7	18.2/38.4	+20.6(10)	+20.4
J_{1b}	5,8	μ_3-OH/μ_3-OH/μ-O_2PO_2H	3.035	95.3/95.4	30.78	139.1/140.0	125.1/129.9	14.6/24.3	+17.9(11)	+19.8
J_{1c}	9,12	μ_3-OH/μ_3-OH/μ-O_2PO_2H	2.979	95.7/96.0	17.07	141.8/141.2	123.0/128.0	9.7/12.3	+19.1(11)	+20.3
J_2	1,2	μ_3-OH/μ_3-OH/μ-O_2PO_2H	3.019	96.3/94.7	30.39	139.5/143.2	126.8/134.5	9.2/11.8	+16.5(13)	+19.2
J_{2b}	5,6	μ_3-OH/μ_3-OH/μ-O_2PO_2H	2.980	93.5/93.2	33.67	140.1/140.7	122.9/126.3	27.6/35.4	+18.9(11)	+20.1
J_{2c}	9,10	μ_3-OH/μ_3-OH/μ-O_2PO_2H	2.949	94.4/95.5	18.06	142.2/146.5	124.3/130.1	16.8/18.0	+19.9(10)	+21.2
J_3	1,3	μ_3-OH/μ_3-OH/μ-O_2PO_2	2.983	94.1/93.5	32.76	141.4/140.8	123.1/127.5	24.9/38.6	+21.7(11)	+21.7
J_{3b}	5,7	μ_3-OH/μ_3-OH/μ-O_2PO_2	3.008	94.7/93.8	32.69	140.5/143.6	119.1/129.7	22.6/39.8	+19.9(10)	+21.4
J_{3c}	9,11	μ_3-OH/μ_3-OH/μ-O_2PO_2	2.978	95.2/95.5	20.04	143.4/147.3	121.9/124.0	25.1/39.2	+21.4(11)	+21.2
J_4	3,4	μ_3-OH/μ-O_2PO_2	3.613	127.4		30.56	125.3/125.8	43.6/43.8	–29.2(9)	–31.6
J_{4b}	7,8	μ_3-OH/μ-O_2PO_2	3.620	128.9		34.37	125.5/126.3	44.3/44.5	–36.0(1)	–36.6
J_{4c}	11,12	μ_3-OH/μ_3-OPO_3	3.262	110.2/92.7	5.3	144.0/145.3			–5.0(10)	–6.1
J_5	2,3	μ_3-OH/μ-O_2PO_2	3.626	126.7		33.08	126.3/125.3	44.6/45.7	–30.7(10)	–31.8
J_{5b}	6,7	μ_3-OH/μ-O_2PO_2	3.652	128.4		29.20	125.4/125.5	45.4/45.6	–34.8(10)	–36.3
J_{5c}	10,11	μ_3-OH/μ_3-OPO_3	3.297	110.3/93.5	3.7	142.9/146.4			–6.4(10)	–7.6
J_6	2,4	μ_3-OH/μ-O_2PO_2	3.600	126.7		31.14	126.3/125.8	44.4/45.0	–27.3(12)	–27.7
J_{6b}	6,8	μ_3-OH/μ-O_2PO_2	3.656	127.3		28.53	125.4/126.3	44.4/44.1	–29.4(11)	–30.1
J_{6c}	10,12	μ_3-OH/μ_3-OPO_3	3.236	108.1/91.2	5.9	150.3/144.8			–5.2(11)	–4.8
J_7	1,5	μ_3-OPO_3/μ-OPO_3H	3.232	98.8/100.6	0.4	144.6/150.9			+5.2(11)	+5.3
J_{7b}	5,9	μ_3-OPO_3/μ-OPO_3H	3.073	93.1/95.4	0.2	139.5/149.7			+8.3(14)	+8.8
J_{7c}	1,9	μ_3-OPO_3/μ-OPO_3H	3.057	94.0/94.4	2.8	142.5/147.6			+9.4(13)	+8.8
J_8	1,6	μ-O_2WO_2H	5.072				123.3/126.3	55.4/33.4	+4.8(16)	+0.28
J_{8b}	5,10	μ-O_2WO_2H	4.996				125.1/130.1	51.2/18.0	–1.5(10)	+0.52
J_{8c}	2,9	μ-O_2WO_2H	5.052				134.5/123.0	11.8/49.4	–3.0(11)	+0.54
J_9	4,5	μ-O_2WO_2H	5.277				126.7/122.9	38.4/20.5	+2.9(16)	+0.48
J_{9b}	9,8	μ-O_2WO_2H	5.217				124.3/129.9	27.5/24.3	+1.2(12)	+0.78
J_{9c}	1,12	μ-O_2WO_2H	5.179				126.8/128.0	32.2/20.8	–6.2(12)	+0.67
J_{10}	4,6	μ-O_2WO_2H	5.540				126.7/126.3	49.3/10.4	–5.2(16)	–0.30
J_{10b}	8,10	μ-O_2WO_2H	5.610				129.9/130.1	49.8/21.9	–3.1(11)	–3.4
J_{10c}	2,12	μ-O_2WO_2H	5.624				134.5/128.0	23.3/50.8	+0.8(3)	–5.3
J_{11}	1,7	μ-O_2PO_2	5.287				236.9/129.7	24.0/39.8	–1.58(14)	+0.60

J_{11b}	5,11	μ-O_2PO_2	5.118	240.9/124.0	30.3/39.2	–3(2)	+0.46
J_{11c}	9,3	μ-O_2PO_2	5.151	238.1/127.5	24.7/38.6	+1.0(15)	+0.67
J_{12}	5,3	μ-O_2PO_2	5.052	240.9/127.5	60.6/38.6	+3(3)	+0.08
J_{12b}	9,7	μ-O_2PO_2	5.009	238.1/129.7	58.1/39.8	+1.1(18)	+0.06
J_{12c}	1,11	μ-O_2PO_2	4.983	236.9/124.0	56.9/39.2	+1.2(16)	+0.15
J_{13}	3,7	μ-O_2PO_2	5.523	232.5/129.7	7.6/42.7	–2(3)	+0.72
J_{13b}	7,11	μ-O_2PO_2	5.438	230.3/124.0	2.5/50.8	+3(2)	+0.50
J_{13c}	3,11	μ-O_2PO_2	5.529	236.0/127.5	9.6/47.9	–1.6(17)	+0.33

[a]Labeling in **Figure S34**. [b]Intermetalic distance in angstroms. [c]Angles (in degrees) described in **Figure S35**. [d]Average values in cm^{-1} calculated on the full experimental geometry. [d]Values (in cm^{-1}) obtained on the simplified Ni_{12} model. Standard deviations in parentheses. [e]Values (in cm^{-1}) obtained on the simplified Ni_2Zn_{10} model.

Table S14. Axial (*D*) and rhombic (*E*) contributions to the local *zfs* tensors and the average *g*-factor for the *S* = 1 ground state obtained from CASSCF calculations on the $NiZn_{11}$ model of compound **{$Ni_{12}W_{30}$}**, and geometrical distortions from the ideal octahedron (OC-6) estimated from shape measures (*Sh*). Note that an observable difference in *D* values for each Ni center arises from the central Ni^{II} ions being non-equivalent in their coordination environment to the rest (**Table S10**).[59]

Site[a]	D^b	*E/D*	g_{avg}	*Sh*(OC-6)[c]
Ni_1	–1.7	0.240	2.353	0.549
Ni_2	+10.6	0.025	2.370	0.390
Ni_3	+15.5	0.062	2.390	0.667
Ni_4	+12.9	0.049	2.386	0.473
Ni_5	–2.4	0.223	2.352	0.520
Ni_6	+13.5	0.019	2.379	0.455
Ni_7	+14.7	0.053	2.403	0.679
Ni_8	+10.9	0.082	2.376	0.449
Ni_9	–2.5	0.244	2.344	0.569
Ni_{10}	+12.5	0.137	2.391	0.533
Ni_{11}	+13.1	0.087	2.380	0.526
Ni_{12}	+12.3	0.088	2.381	0.513

[a]Numbering used as in **Table S10**. [b]Values in cm^{-1}. [c]No null and larger values correspond to a greater distortion of the ideal geometry.

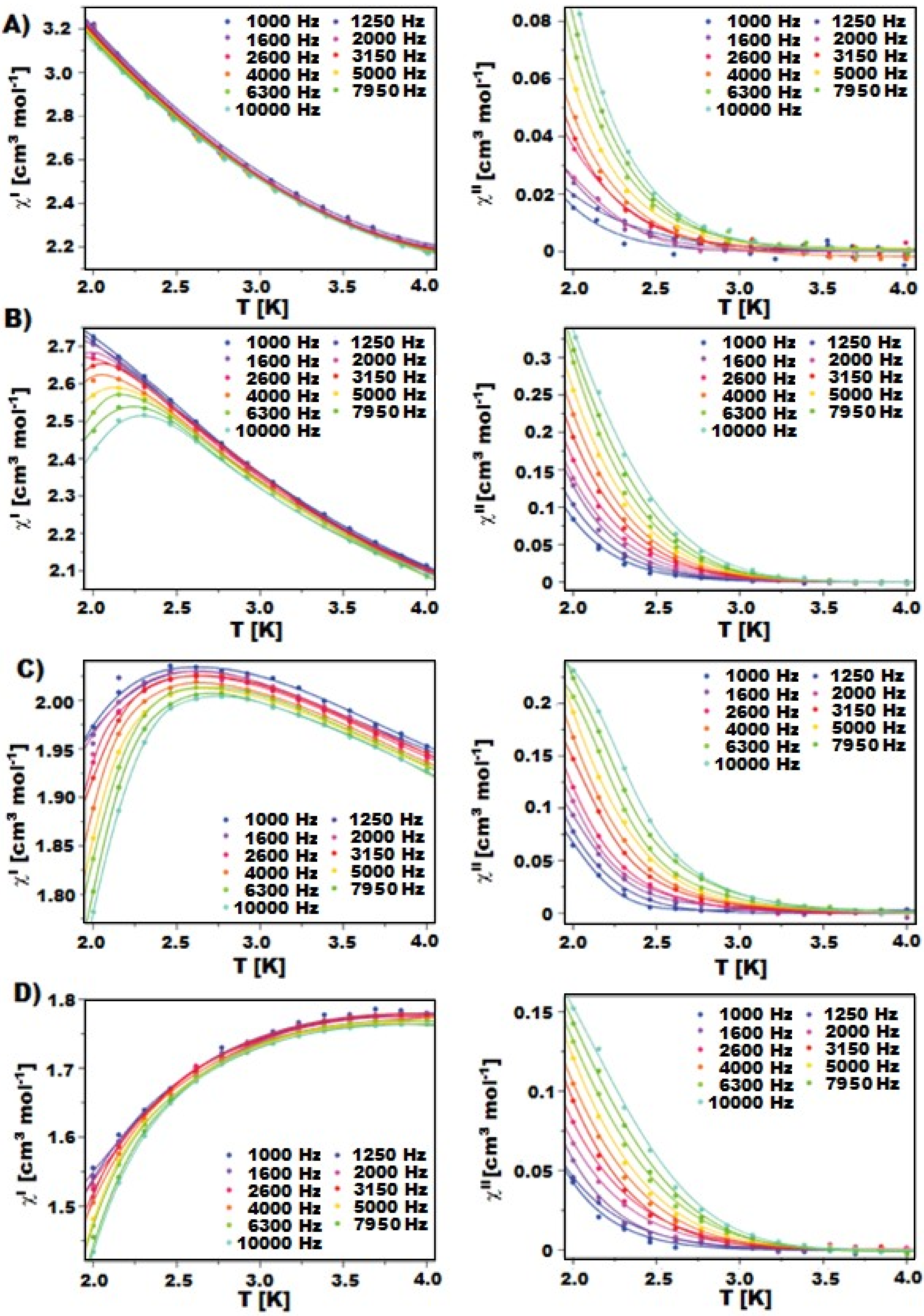

Figure S38. Temperature dependence of χ_M' (left) and χ_M'' (right) ac susceptibilities for $K_{11}Na_{10}$-**{$Ni_{12}W_{30}$}** under dc-applied static fields of (a) 0.00, (b) 0.25, (c) 0.50, and (d) 0.75 T with a ±0.5 mT oscillating field at frequencies in the range 1.0–10 kHz. The solid lines are only eye guides.

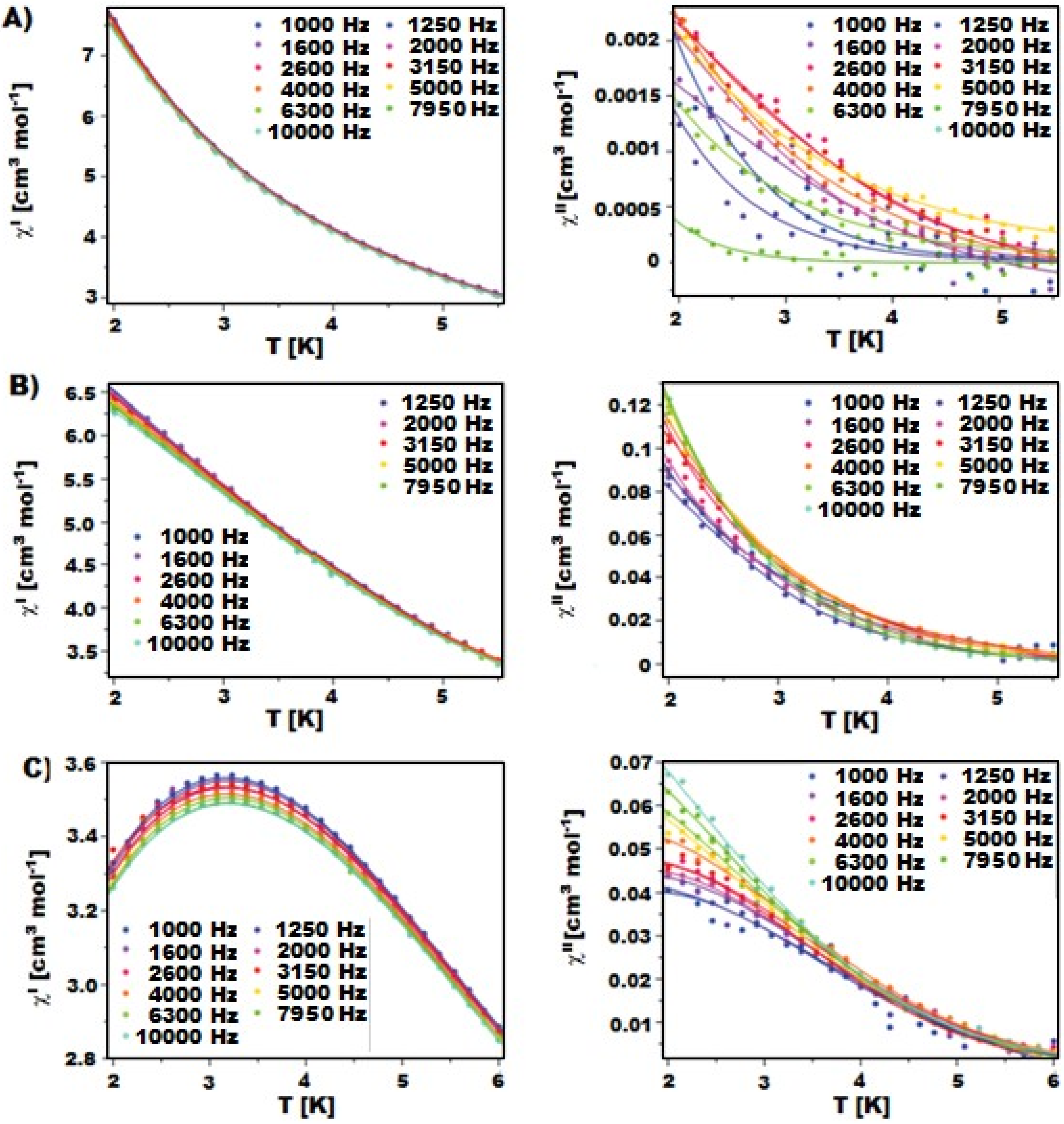

Figure S39. Temperature dependence of χ_M' (left) and χ_M'' (right) ac susceptibilities for $K_{14}Na_7$-**{$Ni_{12}W_{27}$}** under dc-applied static fields of (a) 0.00, (b) 0.25, and (c) 0.50 T with a ±0.5 mT oscillating field at frequencies in the range 1.0–10 kHz. The solid lines are only eye guides.

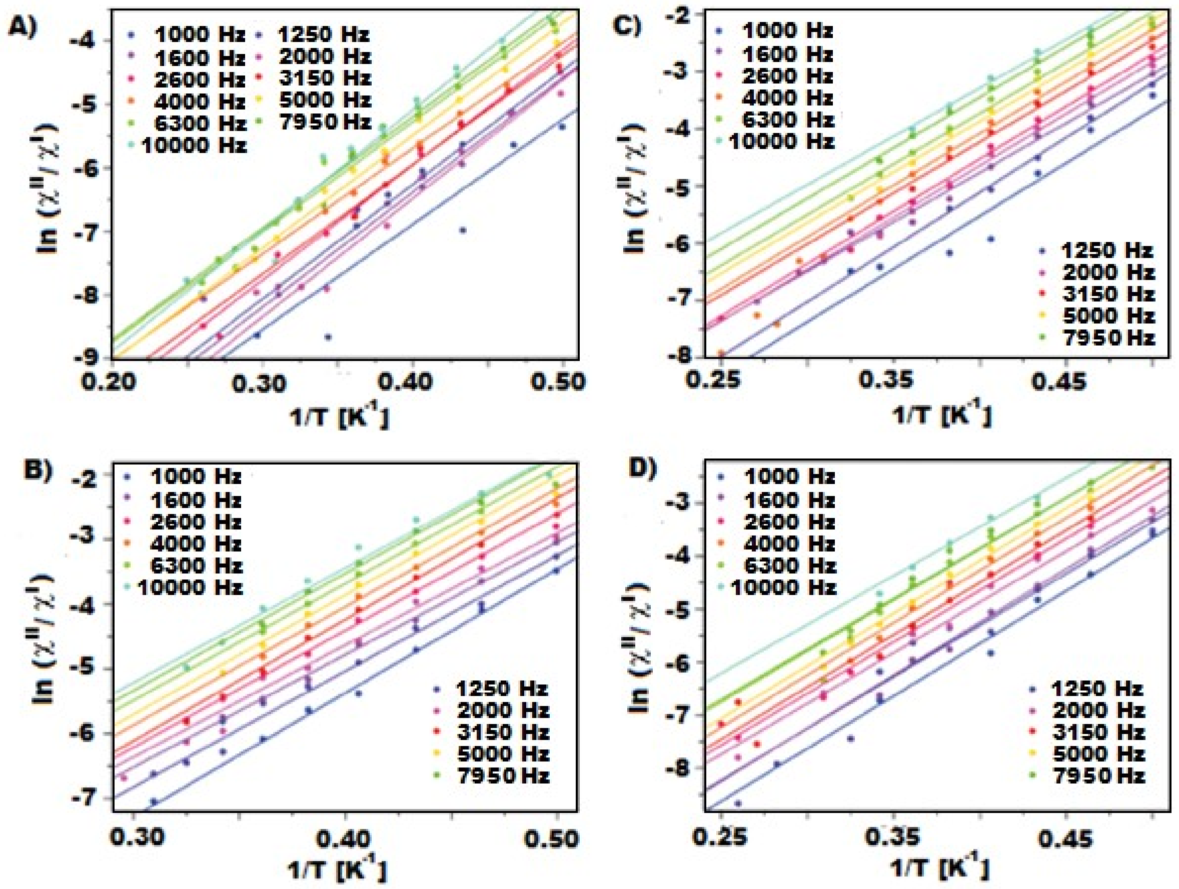

Figure S40. Ln(χ_M''/χ_M') *vs* 1/T plots for $K_{11}Na_{10}$-**{$Ni_{12}W_{30}$}** under applied fields of (a) 0.00, (b) 0.25, (c) 0.50, and (d) 0.75 T in the 1−10 kHz frequency range. The solid lines are the best linear fits for each frequency.

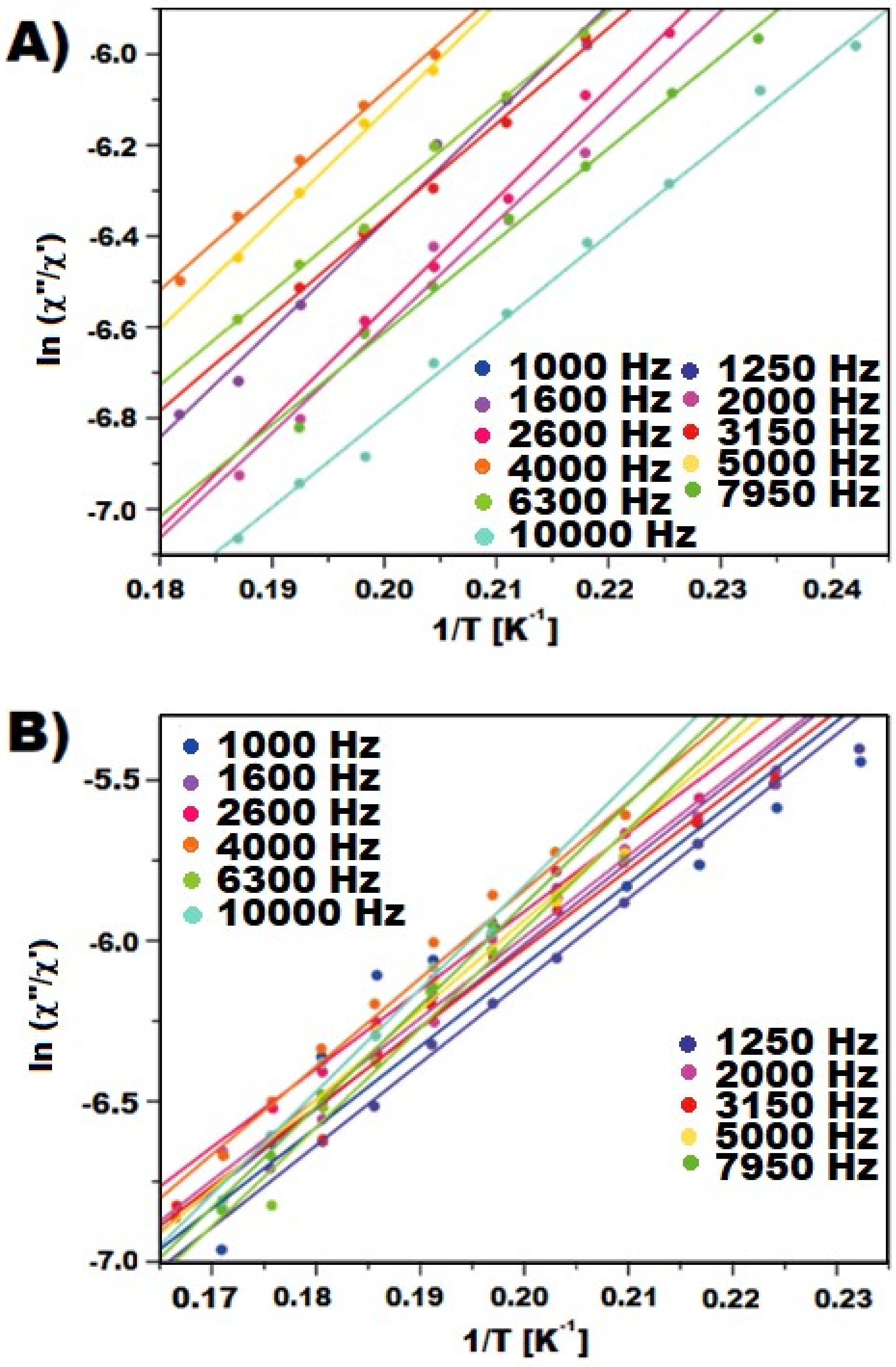

Figure S41. Ln(χ_M''/χ_M') *vs* 1/T plots for $K_{14}Na_7$-**{Ni$_{12}$W$_{27}$}** under applied fields of (a) 0.25, and (b) 0.50 in the 1−10 kHz frequency range. The solid lines are the best linear fits for each frequency.

10.2. Magnetic behavior of $K_{11}Na_{10}$-**{Ni$_{12}$W$_{30}$}** and $K_{14}Na_7$-**{Ni$_{12}$W$_{27}$}** in solution

The Evans method is a powerful technique that can be applied to study the magnetic behavior of solution state metal complexes using NMR spectroscopy.[62] Aqueous solutions containing $K_{14}Na_7$-**{Ni$_{12}$W$_{27}$}** (2.6 mM, in a D_2O/H_2O 50:1 mixture) or $K_{11}Na_{10}$-**{Ni$_{12}$W$_{30}$}** (3.2 mM, in a D_2O/H_2O 50:1 mixture) were prepared and displayed to ^1H NMR spectroscopic measurements along with an internal reference containing the D_2O/H_2O 50:1 solvent mixture in the absence of any paramagnetic solute (**Figure S42**).

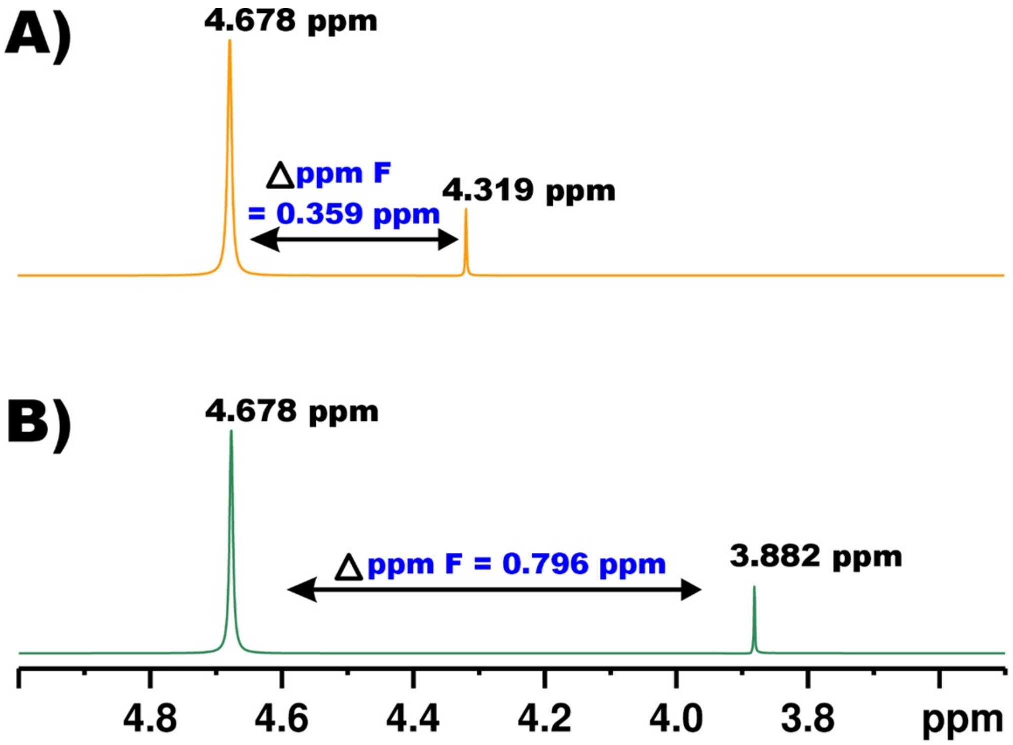

Figure S42. ^1H NMR spectra containing aqueous solutions (D_2O/H_2O 50:1) of **A)** $K_{14}Na_7$-**{Ni$_{12}$W$_{27}$}** and **B)** $K_{11}Na_{10}$-**{Ni$_{12}$W$_{30}$}** with an internal reference (D_2O/H_2O 50:1). Both spectra show the H_2O singlet peak (4.678 ppm) arising from the internal reference and a singlet that is shifted upfield (4.319 ppm in the case of $K_{14}Na_7$-**{Ni$_{12}$W$_{27}$}**, 3.882 ppm in the case of $K_{11}Na_{10}$-**{Ni$_{12}$W$_{30}$}**) due to paramagnetic interactions with the corresponding dissolved polyanion.

By using **equation S5**, the difference in chemical shift of the solvent (**Figure S42**) gives information about the paramagnetic solute's magnetic susceptibility χ_M in solution being χ_M = 0.0603 cm^3/mol ($K_{11}Na_{10}$-**{Ni$_{12}$W$_{30}$}**) and 0.033 cm3/mol ($K_{14}Na_7$-**{Ni$_{12}$W$_{27}$}**), respectively.

$$\chi_M = \frac{3\Delta f}{4\pi F c};$$ (**Equation S5**),

where χ_M is the magnetic susceptibility [cm^3/mol], Δf is the frequency difference [Hz] between the shifted resonance and the pure solvent resonance, F is the spectrometer radiofrequency [Hz], c is the molar concentration of the corresponding solute [mol/mL]

Using **equation S6**, information about the solute's magnetic moment μ in solution can be obtained thereby giving values of μ = 8.89 μ_B ($K_{11}Na_{10}$-**{Ni$_{12}$W$_{30}$}**) and μ = 11.99 μ_B ($K_{14}Na_7$-**{Ni$_{12}$W$_{27}$}**), respectively.

$$\mu = \sqrt{8\chi_M T};$$ (**Equation S6**),

where χ_M is the magnetic susceptibility [cm^3/mol], T is the temperature [K], μ is the magnetic moment measured in units of Bohr magneton, μ_B.

11. High-Frequency/High-Field Electron Paramagnetic Resonance (HFEPR)

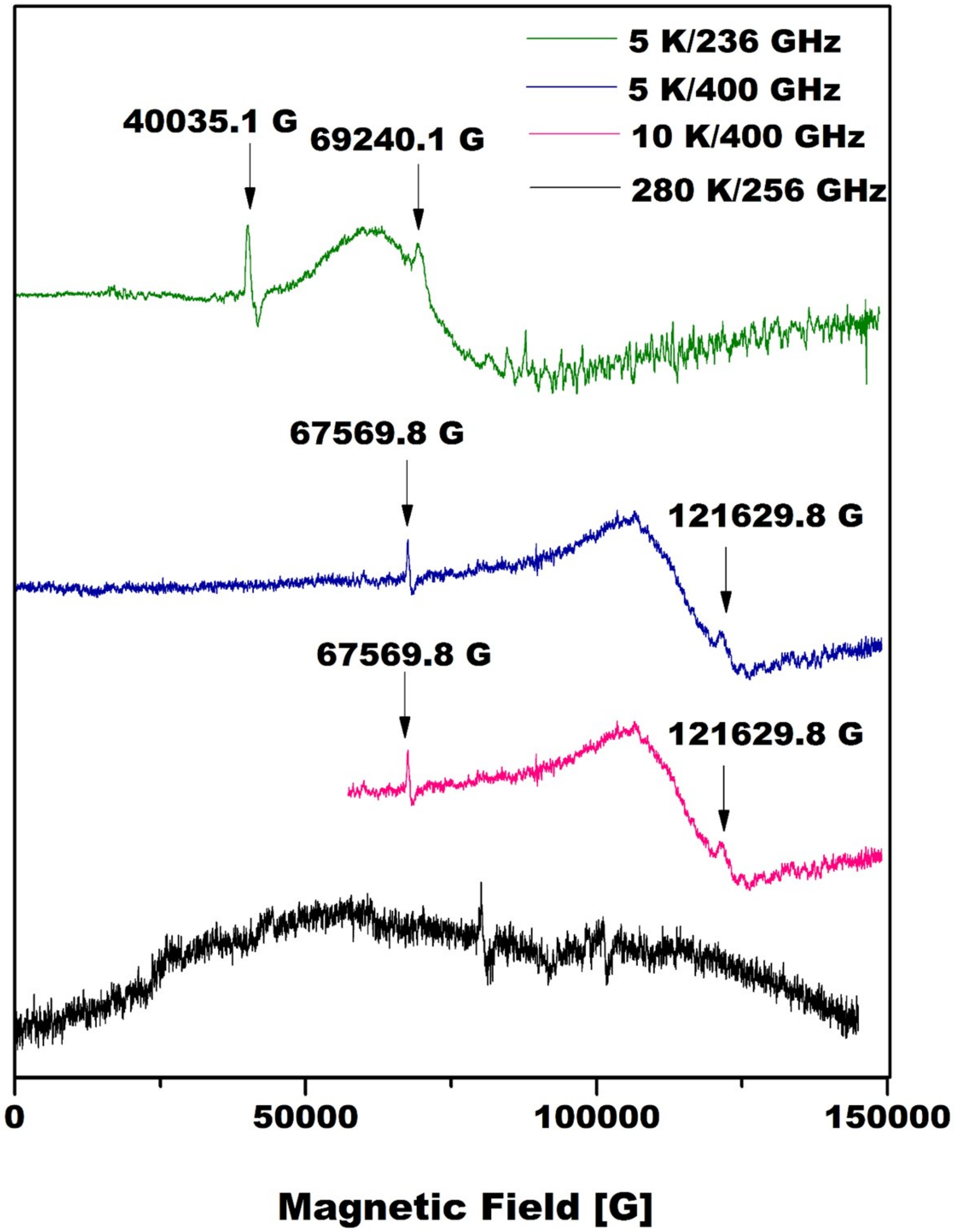

Figure S43. HFEPR spectra of $K_{11}Na_{10}$-**{$Ni_{12}W_{30}$}** at 5 K and 236 GHz (green), 5 K and 400 GHz (blue), 10 K and 400 GHz (magenta), and 280 K and 256 GHz (black).

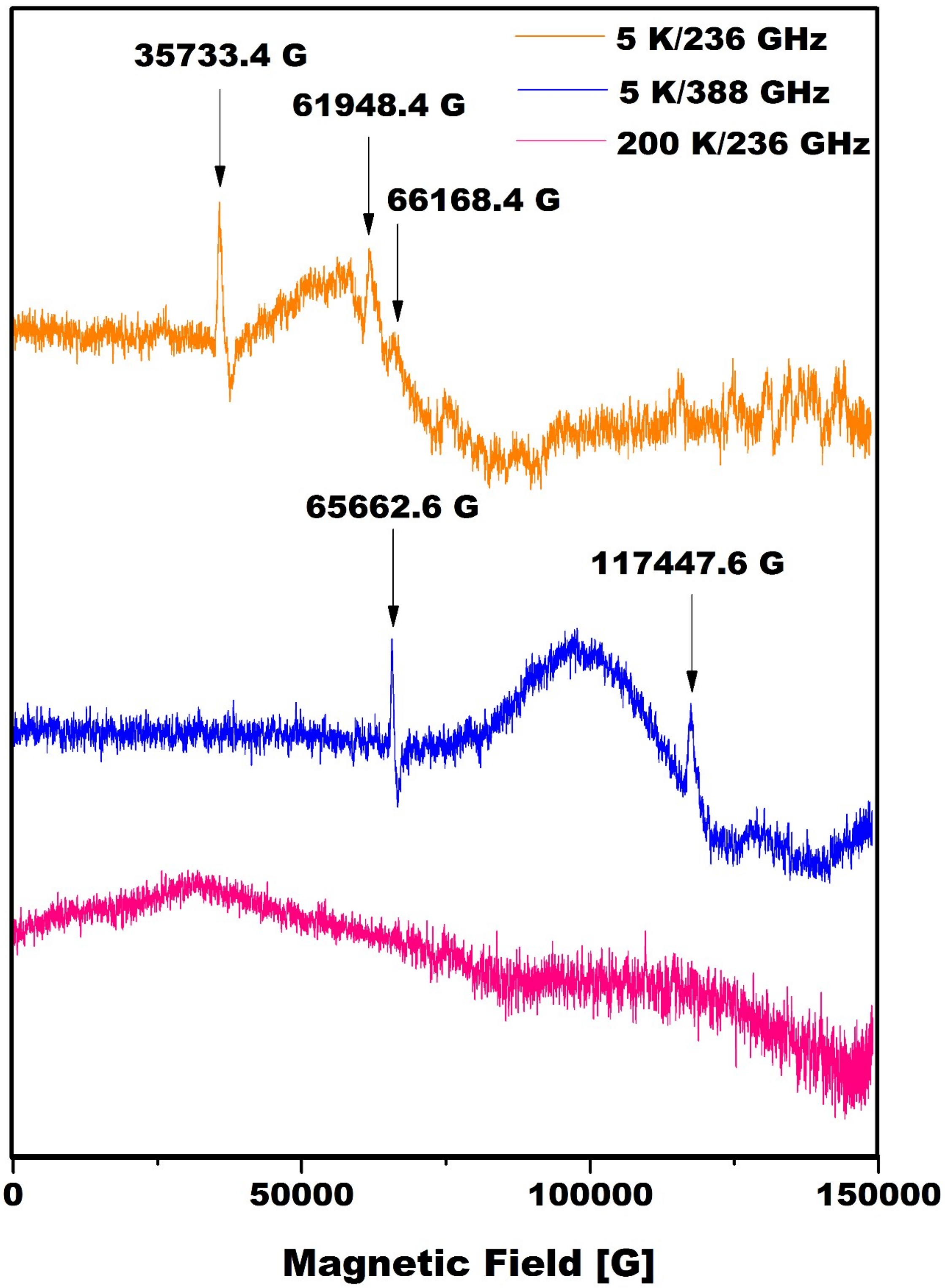

Figure S44. HFEPR spectra of $K_{14}Na_7$-**{Ni$_{12}$W$_{27}$}** at 5 K and 236 GHz (orange), 5 K and 388 GHz (blue), and 200 K and 236 GHz (magenta).

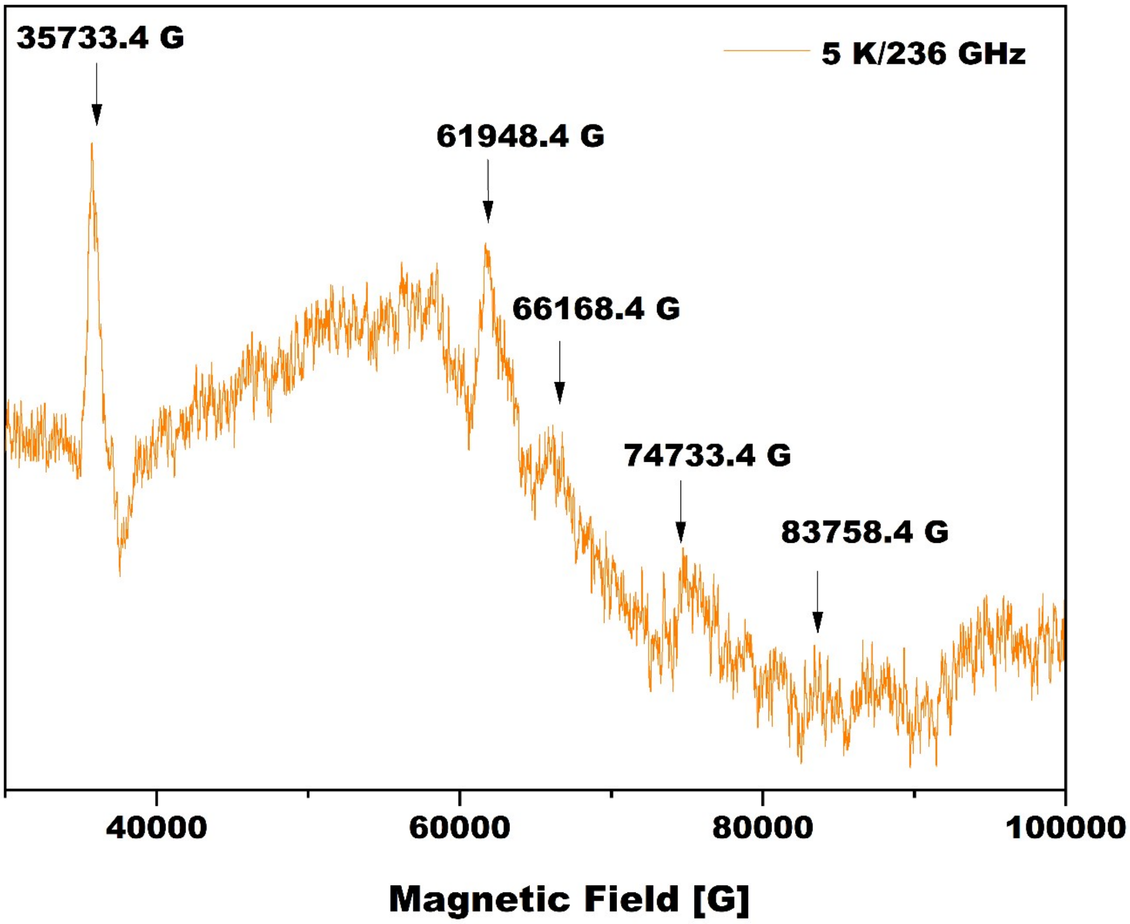

Figure S45. HFEPR spectrum of $K_{14}Na_7$-**{$Ni_{12}W_{27}$}** at 5 K and 236 GHz in the region 30000 – 100000 G.

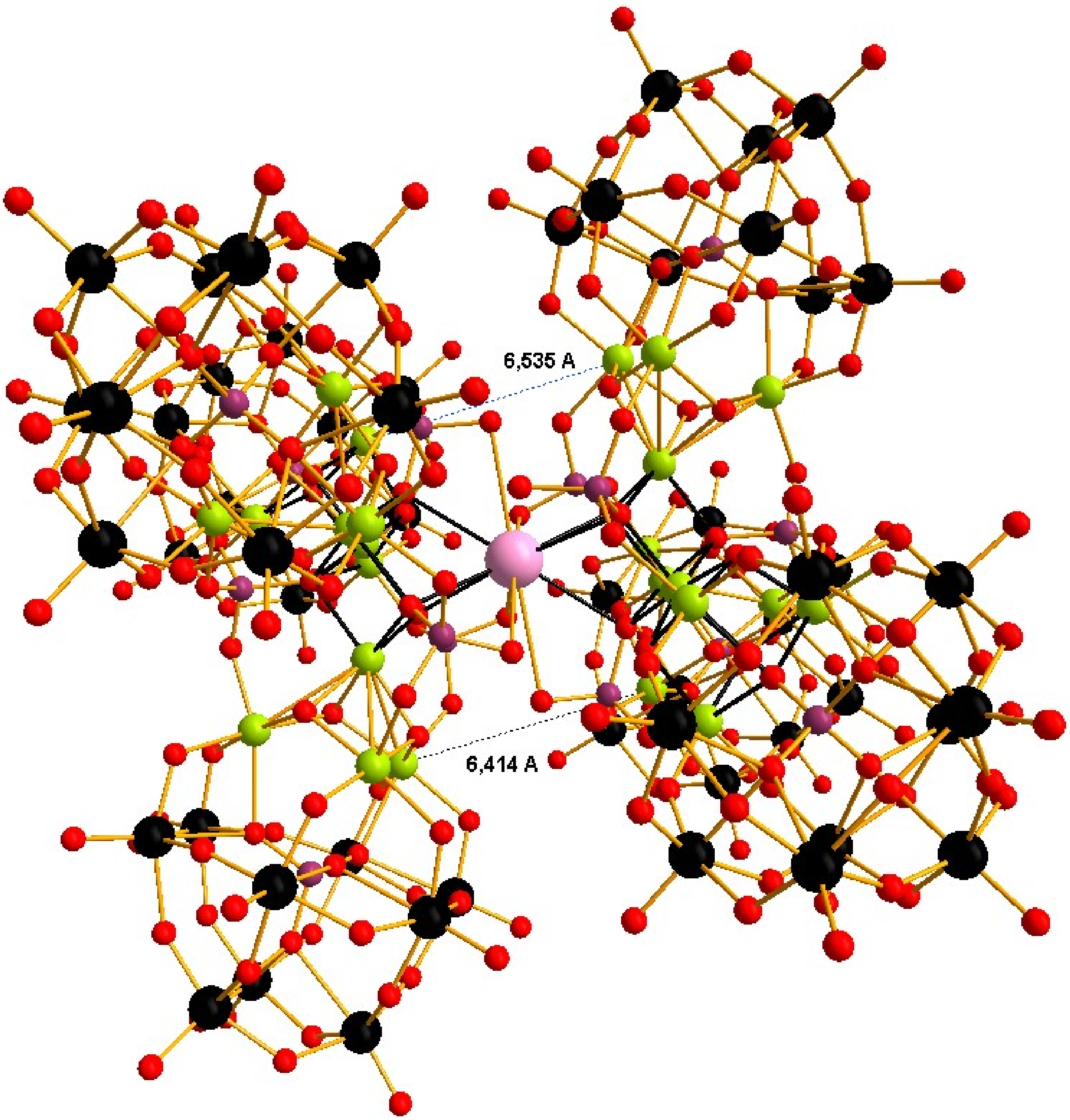

Figure S46. Balls and sticks representation of the **$\{Ni_{12}W_{27}\}_2$** supradimer showing the short Ni…Ni distances of 6.414 Å (**Ni9…Ni9**) and 6.535 Å (**Ni2…Ni5**) giving rise to non-negligible dipolar AF interactions between the Ni_{12} cores of two different **$\{Ni_{12}W_{27}\}$** units which accounts for the weak intensity of the HFEPR signal at ~66168.4 G. Color code. W, black balls; Ni^{II}, lime balls; K^+, candy floss balls; O, red balls; P^V, purple balls.

12. Hydrogen Evolution (HER) experiments

12.1. Pre-catalytic stability study.

To probe the solution stability of **{$Ni_{12}W_{30}$}** and **{$Ni_{12}W_{27}$}**, time-dependent UV-vis spectra were recorded in 11:33:4:2 vol. % CH_3CN/DMF/H_2O/TEOA solvent mixtures. The UV-vis spectra of TBA-**{$Ni_{12}W_{30}$}** and TBA-**{$Ni_{12}W_{27}$}** (TBA = tetrabutylammonium) display absorption maxima at 285 nm (**{$Ni_{12}W_{30}$}**, **Figure S31 A**) and 265 nm (**{$Ni_{12}W_{27}$}**, **Figure S31 B**) corresponding to the O→W ligand−to−metal charge−transfer (LMCT)[63] as well as the d-d transitions typical for octahedrally coordinated NiII metal centers at 688 nm (**{$Ni_{12}W_{30}$}**, **Figure S31 C**) and 700 nm (**{$Ni_{12}W_{27}$}**, **Figure S31 D**), respectively.[64] All observed peaks remain unchanged for at least 60 min, mimicking the photocatalytic conditions and thereby suggesting pre-catalytic stability of **{$Ni_{12}W_{30}$}** and **{$Ni_{12}W_{27}$}** until H_2 saturation is reached in the HER experiments (**Figure 3**).

12.2. HER – activity studies on $TBA_{13}Na_8$-**{$Ni_{12}W_{30}$}** and $TBA_{13}Na_8$-**{$Ni_{12}W_{27}$}**.

Figure 3 shows HER profiles for **{$Ni_{12}W_{30}$}** and **{$Ni_{12}W_{27}$}** obtained from 20 μM catalytic solutions. In both cases, the H_2 evolution follows a sigmoidal profile and reaches saturation after around 60 minutes of illumination. Control experiments with longer light exposure confirmed that no additional H_2 can be generated after this point. Note that only negligible HER (2-3 % of the Ni-PT-catalyzed reaction) could be detected in absence of any catalyst due to direct PS* reduction (**Figure S47**). Moreover, significantly lower H_2 amounts were detected when using 20 μM $Ni(NO_3)_2$ solutions (**Figure S47**), which confirms the catalytic role of the studied **Ni-PT**s. No H_2 was generated in the absence of TEOA or sensitizer (**Figure S47**), demonstrating the validity of the experimental setup.

The amounts of H_2 measured at HER saturation level were translated into turnover numbers (TONs) and yield 36.7 and 38.8 for **{$Ni_{12}W_{30}$}** and **{$Ni_{12}W_{27}$}** (at 20 μM), respectively (**Table 1**). These similar values suggest that the structural differences between **{$Ni_{12}W_{30}$}** and **{$Ni_{12}W_{27}$}** (type of capping ligands, core connectivity) do not contribute to their WRC performance at the investigated catalyst concentration. Importantly, the measured TON values are significantly higher than that of **{Ni_4W_{18}}** (11.4), which manifests the superior performance of the reported **Ni-PT**s over the benchmark WRC even when normalized to the number of Ni-centers. This activity trend is further in line with electronic structure elucidation carried out using diffuse reflectance spectroscopy (DRS) for band gap values (**Figures S17**-**S22**) and cyclic voltammetry for the values of lowest unoccupied and highest occupied molecular orbitals (LUMO/HOMO) of all three compounds (**Figures S23-S25**).

HER performance of **{$Ni_{12}W_{30}$}** and **{$Ni_{12}W_{27}$}** was subsequently evaluated as a function of **Ni-PT** concentration in the lower range between 2 and 10 μM. **Figure S48** indicates that the obtained HER profiles are similar to those from 20 μM experiments shown in **Figure 3**. However, the lower concentrations of **{$Ni_{12}W_{30}$}** and **{$Ni_{12}W_{27}$}** did not yield proportionally lower H_2 amounts considering that the decrease from 20 to 2 μM only resulted in a 2.6 and 3-fold HER drop for **{$Ni_{12}W_{30}$}** and **{$Ni_{12}W_{27}$}**, respectively. This indicates that catalyst concentration is not the limiting factor for the investigated systems and hence suggests limiting contributions by the sensitizer or TEOA concentration.

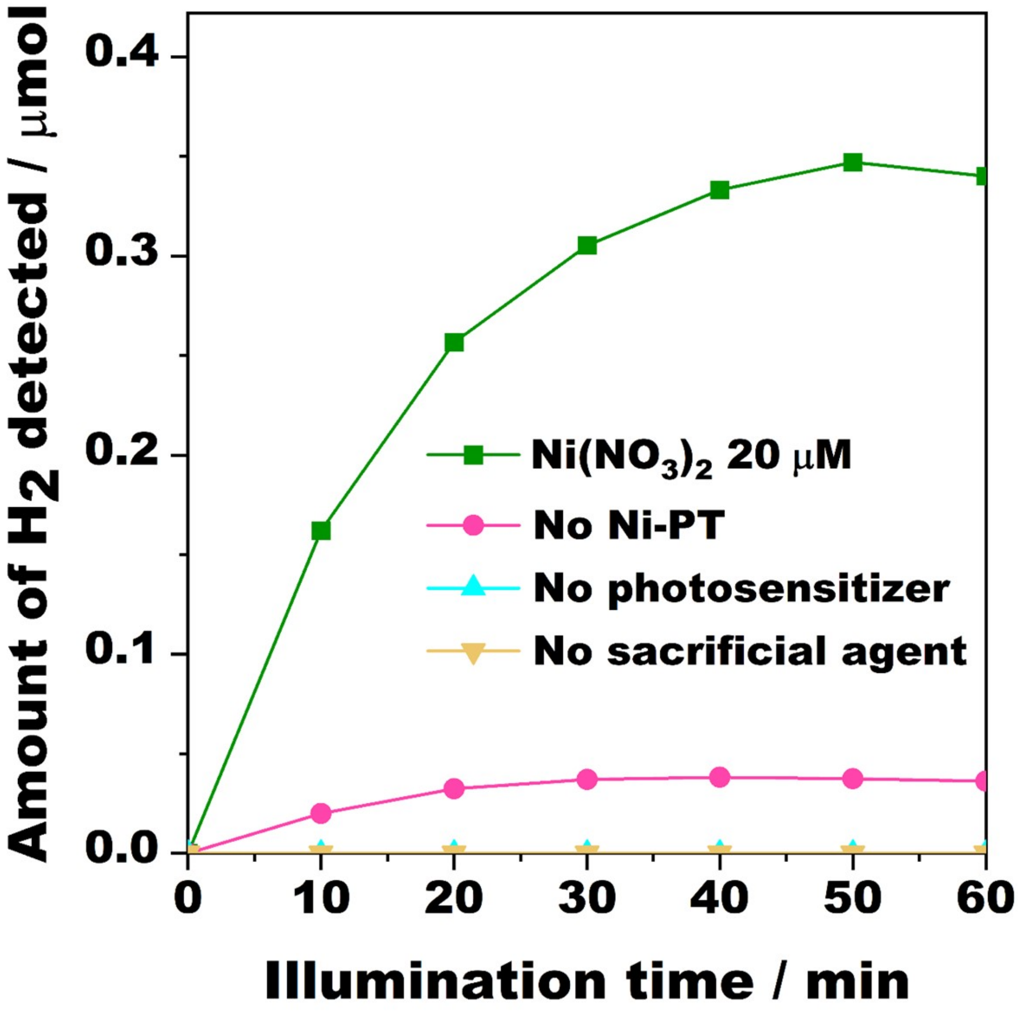

Figure S47. Control experiments aiming to verify the importance of the 3-component system and reveal the potential HER activity due to Ni-leaching. Experiments performed in the absence of any catalyst (*i.e.* only PS, SA, and solvent), sensitizer (*i.e.* only $TBA_{13}Na_8$-**{$Ni_{12}W_{30}$}**, SA and solvent) and sacrificial proton donor (*i.e.* only $TBA_{13}Na_8$-**{$Ni_{12}W_{30}$}**, PS and solvent), as well as the reference HER experiment, performed using 20 μM $Ni(NO_3)_2$ solution charged with the PS and SA. PT = phosphotungstate

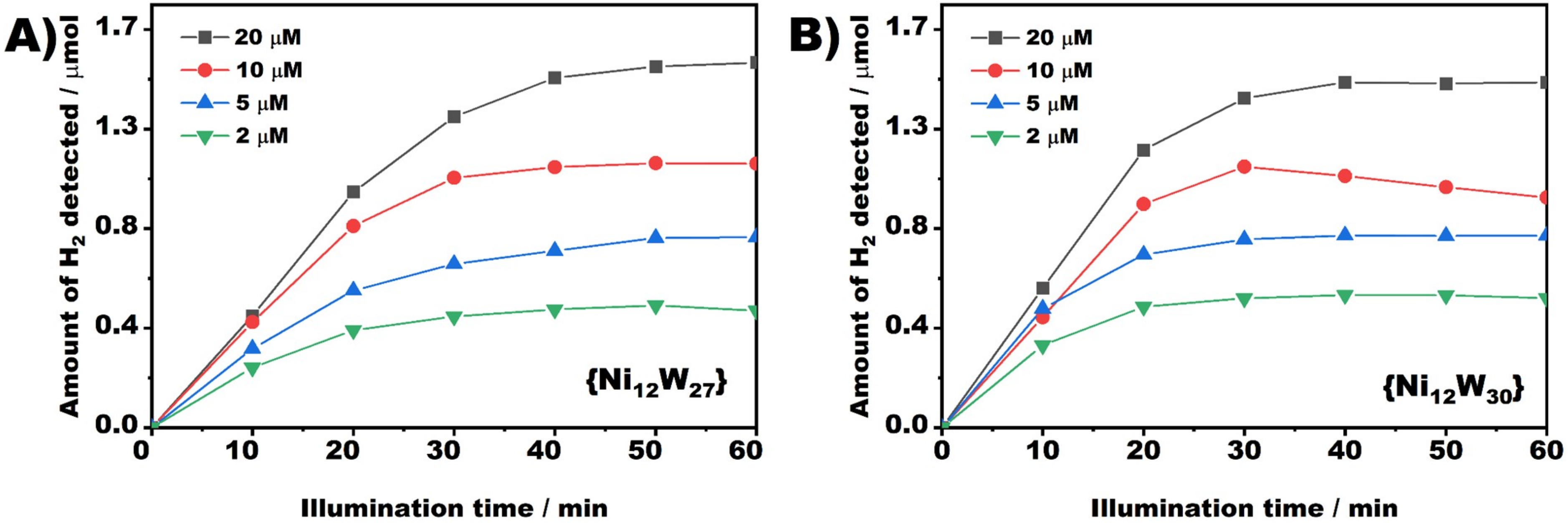

Figure S48. Concentration-dependent HER trends. H_2 evolution profiles for **(A)** TBA$_{13}$Na$_8$-**{Ni$_{12}$W$_{30}$}** and **(B)** TBA$_{13}$Na$_8$-**{Ni$_{12}$W$_{27}$}** obtained from 2-20 µM catalytic solutions. The slight drop of H_2 amount detected for the 10 µM **{Ni$_{12}$W$_{30}$}** sample is related to the sampling procedure which removes a part of the H_2 from the reactor headspace volume (details in the description of the photocatalytic setup given under General Information).

13. POM - HER activity and integrity

13.1 Post-catalytic studies

Post-catalytic studies and re-loading experiments were performed to elucidate the POTs' stability under photocatalytic conditions. First, the catalytic solution of **{$Ni_{12}W_{30}$}** or **{$Ni_{12}W_{27}$}** after the first HER cycle (point x in **Figure 3**) was re-loaded with a sensitizer-TEOA mixture. This second illumination cycle resulted in renewed H_2 evolution, whose extent and profile matched those from the first HER run (**Figure S49 A**). In contrast, the addition of exclusively TEOA yielded only 25% of the original activity (**Figure S49 B**). This proves that the H_2 saturation shown in **Figure 3** is not a result of sole Ni-PT-deactivation but can instead be related to the sensitizer degradation or TEOA depletion. Second, following the completion of HER (point x, **Figure 3**), **{$Ni_{12}W_{30}$}** and **{$Ni_{12}W_{27}$}** were selectively precipitated from the catalytic solution using CsCl.[65] ATR spectra of the isolated Cs-salts match well with the initially recorded tungsten fingerprint areas in the range from 1000-300 cm^{-1} (**Figures S49 A, B**), suggesting structural integrity of both polyanions under turnover conditions. To provide a quantitative assessment of the Ni-PT integrity, the remaining solutions after separating the POT Cs-salts were further analyzed with X-ray fluorescence (XRF) concerning their Ni and W contents (**Table S15**). Elemental data obtained for **{$Ni_{12}W_{30}$}** and **{$Ni_{12}W_{27}$}** show that the catalytic cycle resulted in minor leaching of both Ni and W; however, it can only account for 3 to 10 % of the Ni-PT dissociation, while more than 90 % of the polyanions stayed intact after the catalytic HER cycle. This partial degradation under catalytic conditions have been reported before for similar compounds.[66] Nevertheless, considering the effectiveness of the PS reloading tests in **Figure S50**, the observed Ni-PT degradation seems insignificant compared to the effect of sensitizer instability.

13.2 Post-catalytic precipitation of $TBA_{13}Na_8$-**{$Ni_{12}W_{30}$}** and $TBA_{13}Na_8$-**{$Ni_{12}W_{27}$}**

The photocatalytic reaction was carried out with 100 µM of the corresponding catalyst to obtain the POM in sufficient quantity for post-analysis. After 30 min of illumination, 0.5 mL of a [0.5 M] solution of cesium chloride in a mixture of acetonitrile/H_2O (2:1) was added resulting in the immediate formation of precipitates. The precipitate was centrifuged at 2500 rpm for 5 min and completeness of the precipitation was insured by adding a few drops of the cesium chloride solution to the supernatant. The precipitates were air dried and displayed to IR-spectroscopic analysis (**Figure S49**).

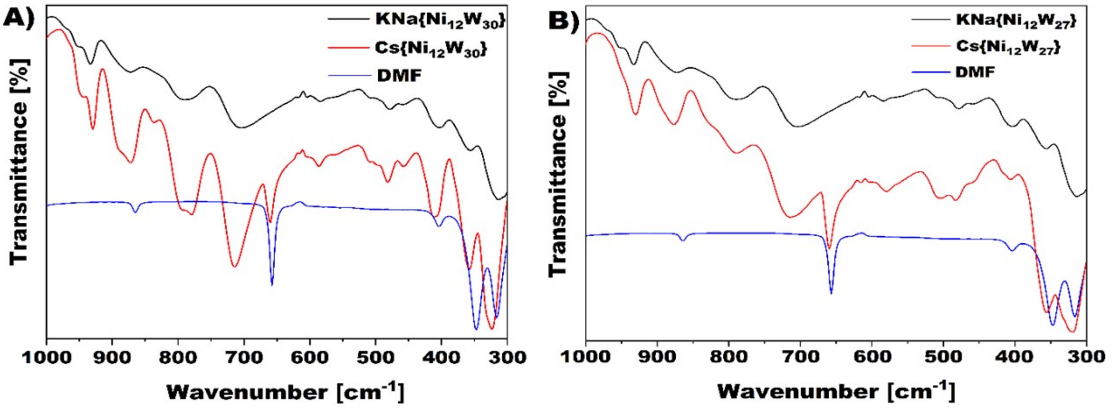

Figure S49. ATR-IR spectra showing the superimposed tungsten fingerprint areas (1000-300 cm^{-1}) of **A)** $K_{11}Na_{10}$-**{$Ni_{12}W_{30}$}** and the precipitated cesium salt Cs**{$Ni_{12}W_{30}$}** as well as **B)** $K_{14}Na_7$-**{$Ni_{12}W_{27}$}** and the precipitated cesium salt Cs**{$Ni_{12}W_{27}$}**. A dominant band at ~660 cm^{-1} in the precipitated Cs-salts arises from residual DMF as shown by the IR spectrum of pure DMF.

13.3 Reloading experiments

To investigate the recyclability of $TBA_{13}Na_8$-**{$Ni_{12}W_{27}$}** and $TBA_{13}Na_8$-**{$Ni_{12}W_{30}$}**, a solution containing $[Ir(ppy)_2(dtbbpy)]^+$ photosensitizer (0.2 mM), TEOA proton donor (0.25 M), and the corresponding POT-WRC (20 µM) in 2 mL of 11:33:4 CH_3CN/DMF/H_2O solvent mixture was irradiated and the H_2 evolution was followed by GC until saturation was reached, indicated by a plateau. The reaction solution was reloaded with 100 µL of a freshly prepared solution of $[Ir(ppy)_2(dtbbpy)]^+$ photosensitizer (3.99 mM) in acetonitrile, 80 µL H_2O and 40 µL TEOA to yield 2.22 mL of a reloaded reaction mixture with 180 µM $[Ir(ppy)_2(dtbbpy)]^+$ photosensitizer, ~137 mM TEOA and 18 µM POT, thereby resembling the initial POT/PS molar ratios of the first reaction cycle. Subsequently, sealing of the reloaded reaction mixture, de-gassing and irradiation initiated the second reaction cycle (**Figure S50**).

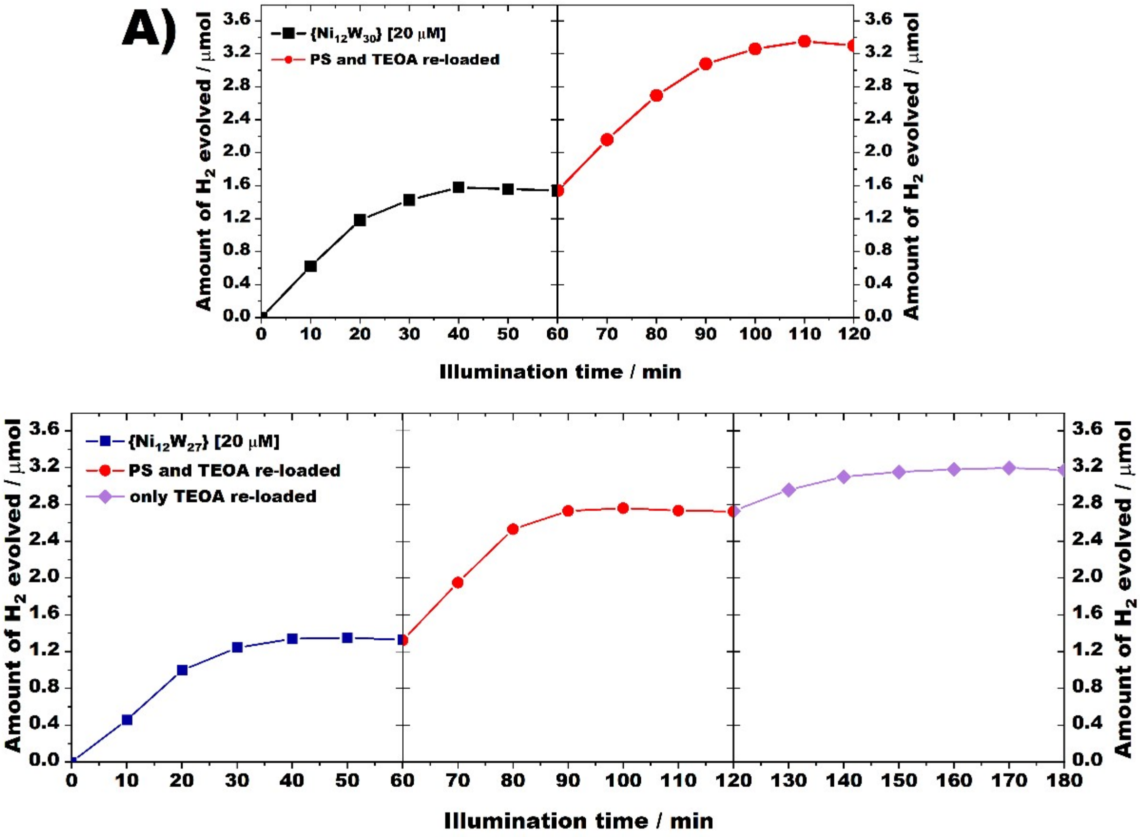

Figure S50. Re-loading experiments aiming to reveal the impact of catalytic solution components on HER performance. For 20 µM **(A)** $TBA_{13}Na_8$-**{$Ni_{12}W_{30}$}** and **(B)** $TBA_{13}Na_8$-**{$Ni_{12}W_{27}$}** after the first 60 min HER cycle (left panels), the reaction volumes were re-charged with a photosensitizer/TEOA/CH_3CN/DMF/water solution (as described above). After degassing, the second photocatalytic run (red curves) yielded significant H_2 evolution close to that of the first HER cycle for both compounds. When only TEOA was re-loaded (lila curve in **B)**, the amount of generated H_2 only accounted to around a quarter of the original activity value.

13.4 Total X-ray fluorescence (TXRF) experiments

Following the post-catalytic precipitation experiments (see subsection 13.1. Post-catalytic precipitation of $TBA_{13}Na_8$-**{$Ni_{12}W_{30}$}** and $TBA_{13}Na_8$-**{$Ni_{12}W_{27}$}**), TXRF analyses of the Ni and W contents present in the isolated supernatants of the corresponding **Ni-PT** solutions were conducted to elucidate on potential leaching and provide a quantitative assessment of the **Ni-PT**s' post-catalytic stability (see General Information section *X-ray fluorescence*). The TXRF results are summarized in the following **Table S15**: The detected Ni/W contents before (**2**, in ppm) and after HER cycle (**4**, in ppm) were evaluated with regard to the theoretical amounts of Ni/W in case complete **Ni-PT** dissociation/decomposition would

take place (**1**, in ppm). The part of the **Ni-PT** that underwent dissociation/leaching before (**3**, in %) and after the HER cycle (**5**, in %) provides a quantitative measure for PT stability. Expressing the Ni/W amounts found by TXRF in % (consideration of mol. % or wt. % would give identical results) of the total amounts of Ni/W present in the original catalytic solutions allows to evaluate instability of the **Ni-PT**s under the turnover condition.

Results. A detectable amount of Ni and W could be measured in the supernatants before photocatalysis (3.1% Ni and 5.4% W for $TBA_{13}Na_8$-**{$Ni_{12}W_{27}$}**; 4.7% Ni and 4.5% W for $TBA_{13}Na_8$-**{$Ni_{12}W_{30}$}**). Considering the precatalytic stability experiments (see sections 8 Cyclic Voltammetry and 9 UV-vis spectroscopy), which suggested long-term stability of both **Ni-PT**s, the observed Ni- and W contents in the solution before HER indicate incomplete precipitation of the anions during the extraction. Hence, the observed pre-catalytic Ni/W contents (**3**, in %) were subtracted from the determined post-catalytic contents (**5**, in %) to assess for the degree of **Ni-PT** leaching/decomposition (**6**, in %). **Table S15** shows (see column **6**) contents of 6.5% for Ni and 10.3% for W for $TBA_{13}Na_8$-**{$Ni_{12}W_{27}$}** as well as 5.0% for Ni and 3.6% for W for $TBA_{13}Na_8$-**{$Ni_{12}W_{30}$}** indicating that not more than ~10% of the Ni/W was leached over the course of the HER cycle. This implies that ~90 % of the polyanions stayed intact allowing for the long-term photocatalytic stability shown in **Figure S50A**.

Table S15. Summary of the TXRF results for $TBA_{13}Na_8$-**{$Ni_{12}W_{27}$}** and $TBA_{13}Na_8$-**{$Ni_{12}W_{30}$}**.

Ni-PT	detected contents	1 [ppm]	2 [ppm]	3 [%]	4 [ppm]	5 [%]	6 [%]
**{$Ni_{12}W_{27}$}**	*Ni*	*12.83*	*0.3975 ± 0.176*	*3.1*	*1.2295 ± 0.193*	*9.6%*	***6.5***
	W	*90.45*	*4.902 ± 0.442*	*5.4*	*14.1605 ± 0.571*	*15.7%*	***10.3***
**{$Ni_{12}W_{30}$}**	*Ni*	*12.83*	*0.5985 ± 0.108*	*4.7*	*1.239 ± 0.179*	*9.7%*	***5.0***
	W	*100.50*	*4.4725 ± 0.269*	*4.5*	*8.1145 ± 0.47*	*8.1%*	***3.6***

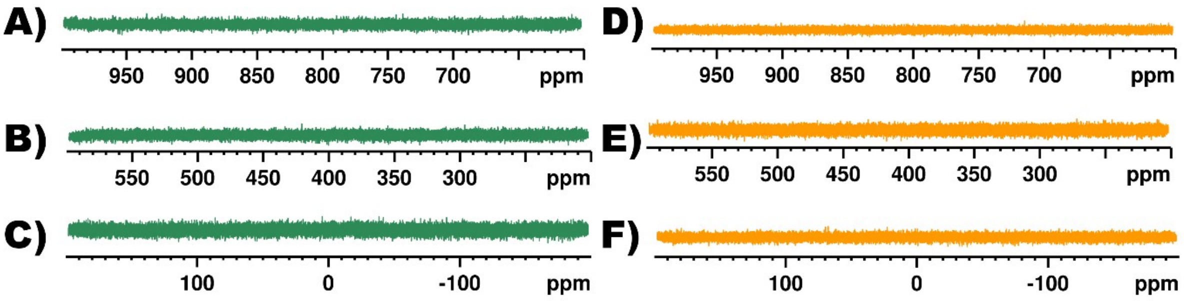

Figure S51. ^{31}P NMR spectra of $TBA_{13}Na_8$-**{$Ni_{12}W_{30}$}** in the range from 1000 to -200 ppm (**A** – **C**) and $TBA_{13}Na_8$-**{$Ni_{12}W_{27}$}** from 1000 to -200 ppm (**D** – **F**). The absence of signals can be attributed to the presence of paramagnetic Ni(II) and the comparably low number of incorporated P centers.

14. Photoluminescence (PL) emission spectroscopy

To explore the photocatalytic mechanism of hydrogen evolution reaction (HER), and to understand the electron transfer kinetics between the reaction solution components, photoluminescence (PL) emission spectroscopy was employed. The PL properties of $[Ir(ppy)_2(dtbbpy)]^+$ in N_2-deaerated CH_3CN:DMF:H_2O (11:33:4) solution were observed using a light excitation at 445 nm. **Figure S52** demonstrates that the photosensitizer's PL emission is quenched by TEOA, $TBA_{13}Na_8$-**$\{Ni_{12}W_{30}\}$** and $TBA_{13}Na_8$-**$\{Ni_{12}W_{27}\}$** in a linear Stern–Volmer behavior depending on the quencher's concentrations (**Figure S52**).

The linear fitting of the Stern-Volmer plot demonstrates that the rate constant for reductive quenching by TEOA is deduced to be 2.7 x 10^7 M^{-1} s^{-1}, whereas rate constants of 8.9 x 10^9 M^{-1} s^{-1} ($TBA_{13}Na_8$-**$\{Ni_{12}W_{27}\}$**) and 13.2 x 10^9 M^{-1} s^{-1} ($TBA_{13}Na_8$-**$\{Ni_{12}W_{30}\}$**) were calculated for the oxidative quenching by the corresponding Ni-PT suggesting that $[Ir(ppy)_2(dtbbpy)]^{+*}$ can undergo both reductive and oxidative quenching if TEOA and the corresponding Ni-PT are present in equimolar concentrations (**Scheme S1**). Considering the 12.5 – fold higher concentration of TEOA (250 μM) as compared to the highest investigated Ni-PT concentration (20 μM) under turnover conditions, a reductive quenching mechanism is proposed for the investigated reaction system. This conclusion is further supported by time-resolved PL experiments allowing to explore the decay kinetics of $[Ir(ppy)_2(dtbbpy)]^{+*}$. The PL decay of $[Ir(ppy)_2(dtbbpy)]^+$ fitted using a biexponential function gives an excited state lifetime of ~117 ns. The lifetime decreases in the presence of TEOA, $TBA_{13}Na_8$-**$\{Ni_{12}W_{27}\}$** and $TBA_{13}Na_8$-**$\{Ni_{12}W_{30}\}$** to yield values of 70.4, 107.6 and 108.1 ns, respectively. The decrease - trend illustrates that TEOA accelerates the quenching kinetics of $[Ir(ppy)_2(dtbbpy)]^+$ most effectively out of the investigated quenchers additionally supporting the proposed reductive quenching pathway.

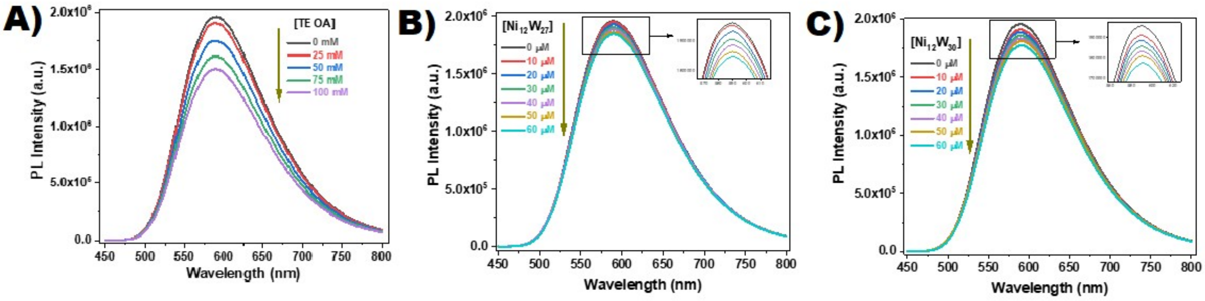

Figure S52. PL emission of $[Ir(ppy)_2(dtbbpy)]^+$ (0.2 mM) excited at 445 nm with the addition of different amounts of **A)** TEOA (0-100 mM), **B)** $TBA_{13}Na_8$-**$\{Ni_{12}W_{27}\}$** (0-60 μM) and **C)** $TBA_{13}Na_8$-**$\{Ni_{12}W_{30}\}$** (0-60 μM). K_q values calculated from linear fitting of Stern-Volmer plots are (**A**) 2.7 x 10^7 M^{-1} s^{-1}, (**B**) 8.9 x 10^9 M^{-1} s^{-1} and (**C**) 13.2 x 10^9 M^{-1} s^{-1}, respectively (**Figure S53**).

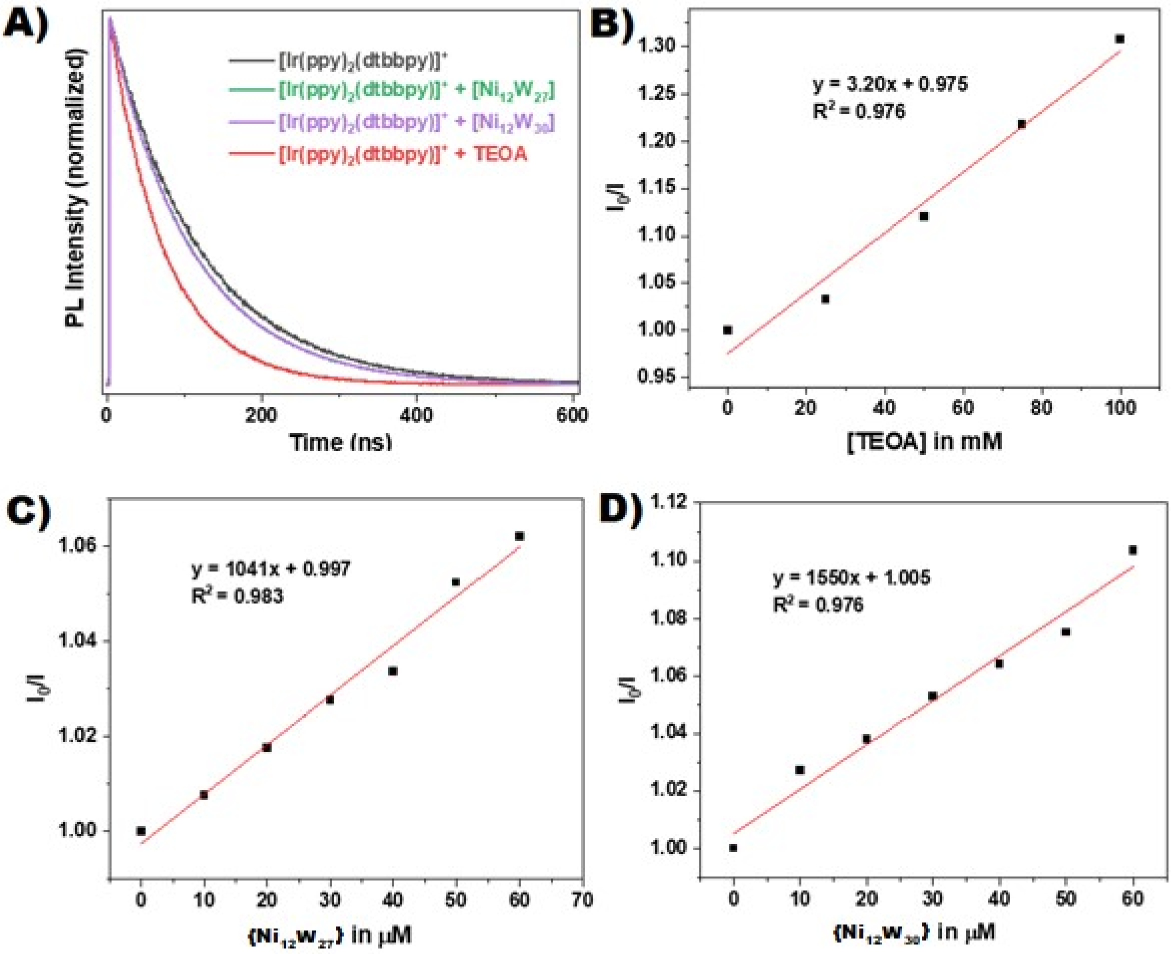

Figure S53. Superimposed time-resolved PL spectra of **A)** $[Ir(ppy)_2(dtbbpy)]^+$ [0.2 mM] without added quencher (black) and after addition of TEOA [0.25 M] (red), $TBA_{13}Na_8$-**$\{Ni_{12}W_{27}\}$** [20 μM] (green), or $TBA_{13}Na_8$-**$\{Ni_{12}W_{30}\}$** [20 μM] (purple); Stern-Volmer plots and linear fits for the emission of $[Ir(ppy)_2(dtbbpy)]^+$ [20 μM] quenched by **B)** TEOA, **C)** $TBA_{13}Na_8$-**$\{Ni_{12}W_{27}\}$** and **D)** $TBA_{13}Na_8$-**$\{Ni_{12}W_{30}\}$**.

Scheme S2. Proposed mechanism for visible-light-driven hydrogen evolution catalyzed by $TBA_{13}Na_8$-**{$Ni_{12}W_{27}$}** and $TBA_{13}Na_8$-**{$Ni_{12}W_{30}$}**. Upon visible-light driven excitation (**I.**) $[Ir(ppy)(dtbbpy)]^+$ is excited to $[Ir(ppy)(dtbbpy)]^{*+}$. The excited photosensitizer can undergo both reductive and oxidative quenching. Oxidative quenching: In a subsequent step, $[Ir(ppy)(dtbbpy)]^{*+}$ is oxidized to $[Ir(ppy)(dtbbpy)]^{2+}$ (**II.**) upon reduction of the corresponding Ni-PT, which is re-oxidized to the initial species by reducing H^+ and forming H_2 (**III.**). The oxidized photosensitizer $[Ir(ppy)(dtbbpy)]^{2+}$ is consecutively reduced to $[Ir(ppy)(dtbbpy)]^+$ by the sacrificial agent TEOA (**IV.**). Reductive quenching: The excited $[Ir(ppy)(dtbbpy)]^{*+}$ is quenched upon reduction by TEOA to give [Ir(ppy)(dtbbpy)] (**II.**). In a consecutive **III.** step, [Ir(ppy)(dtbbpy)] is re-oxidized to $[Ir(ppy)(dtbbpy)]^+$ by the corresponding Ni-PT which reduces H^+ to ultimately form H_2 (**IV.**).

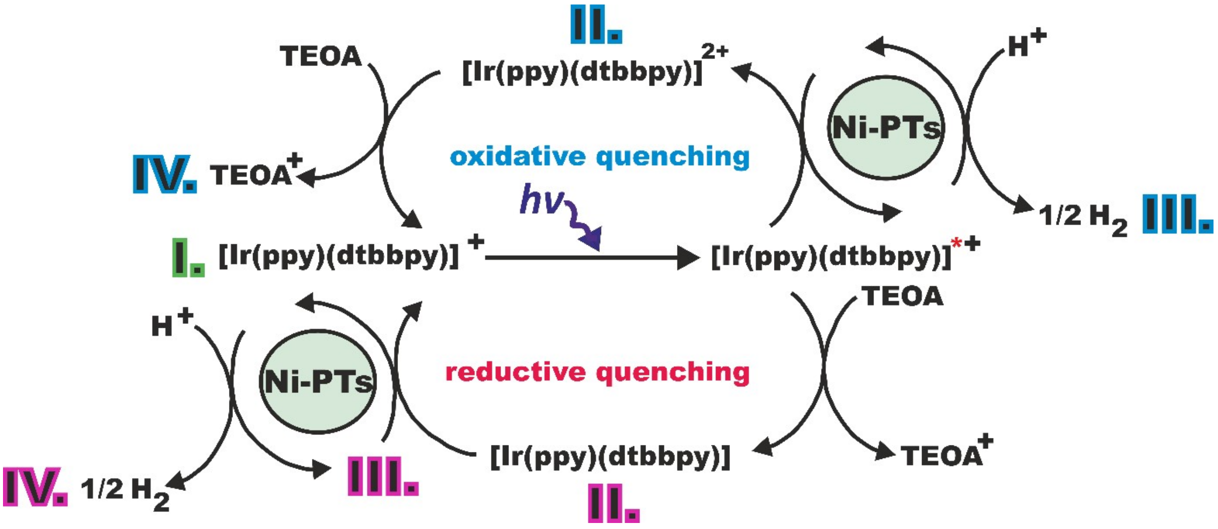

15. References

1 a) C. M. Tourné and G. F. Tourné, *J. Chem. Soc., Dalton Trans.*, 1988, 2411–2420, b) H. Lv, W. Guo, K. Wu, Z. Chen, J. Bacsa, D. G. Musaev, Y. V. Geletii, S. M. Lauinger, T. Lian and C. L. Hill, *J. Am. Chem. Soc.*, 2014, **136**, 14015–14018.

2 Bruker SAINT v7.68A Copyright © 2005-2016 Bruker AXS.

3 G.M. Sheldrick SADABS University of Göttingen, Germany (1996).

4 Sheldrick, G. M. (1996). SHELXS. University of Göttingen, Germany.

5 Sheldrick, G. M. (1996). SHELXL. University of Göttingen, Germany.

6 Dolomanov, O.V., Bourhis, L.J., Gildea, R.J, Howard, J.A.K. & Puschmann, H. , OLEX2, *J. Appl. Cryst.,* **2009**, *42*, 339-341.

7 C. B. Huebschle, G. M. Sheldrick and B. Dittrich, *J. Appl. Cryst.*, **2011**, *44*, 1281-1284.

8 Frisch, M. J.; Trucks, G. W.; Schlegel, H. B.; Scuseria, G. E.; Robb, M. A.; Cheeseman, J. R.; Scalmani, G.; Barone, V.; Mennucci, B.; Petersson, G. A.; Nakatsuji, H.; Caricato, M.; Li, X.; Hratchian, H. P.; Izmaylov, A. F.; Bloino, J.; Zheng, G.; Sonnenberg, J. L.; Hada, M.; Ehara, H.; Toyota, K.; Nakai, H.; Vreven, T.; Montgomery, J. A. J.; Peralta, J. E.; Ogliaro, F.; Bearpark, M.; Heyd, J. J.; Brothers, E.; Kudin, K. N.; Staroverov, V. N.; Kobayashi, R.; Normand, J.; Raghavachari, K.; Rendell, A.; Burant, J. C.; Iyengar, S. S.; Tomasi, J.; Cossi, M.; Rega, N.; Millam, J. M.; Klene, M.; Knox, J. E.; Cross, J. B.; Bakken, V.; Adamo, C.; Jaramillo, J.; Gomperts, R.; Stratmann, R. E.; Yazyev, O.; Austin, A. J.; Cammi, R.; Pomelli, C.; Ochterski, J. W.; Martin, R. L.; Morokuma, K.; Zakrzewski, V. G.; Voth, G. A.; Salvador, P.; Dannerberg, J. J.; Dapprich, S.; Daniels, A. D.; Farkas, Ö.; Foresman, J. B.; Ortiz, J. V.; Cioslowski, J.; Fox, D. J. *Gaussian 09*; *Gaussian*, Inc.: Wallingford CT, **2009**.

9 a) A. D. Becke, *Phys. Rev. A*, 1988, **38**, 3098–3100, b) C. Lee, W. Yang and R. G. Parr, *Phys. Rev. B*, 1988, **37**, 785–789.

10 a) A. Schäfer, H. Horn and R. Ahlrichs, *J. Chem. Phys.*, 1992, **97**, 2571–2577, b) A. Schäfer, C. Huber and R. Ahlrichs, *J. Chem. Phys.*, 1994, **100**, 5829–5835, c) P. J. Hay, *J. Chem. Phys.*, 1977, **66**, 4377–4384, d) P. J. Hay and W. R. Wadt, *J. Chem. Phys.*, 1985, **82**, 270–283, e) W. R. Wadt and P. J. Hay, *J. Chem. Phys.*, 1985, **82**, 284–298, f) P. J. Hay and W. R. Wadt, *J. Chem. Phys.*, 1985, **82**, 299–310.

11 J. Tomasi, B. Mennucci and R. Cammi, *Chem. Rev.*, 2005, **105**, 2999–3094.

12 a) E. Ruiz, A. Rodríguez-Fortea, J. Cano, S. Alvarez and P. Alemany, *J. Comput. Chem.*, 2003, **24**, 982–989, b) E. Ruiz, J. Cano, S. Alvarez and P. Alemany, *J. Am. Chem. Soc.*, 1998, **120**, 11122–11129, c) E. Ruiz, P. Alemany, S. Alvarez and J. Cano, *J. Am. Chem. Soc.*, 1997, **119**, 1297–1303, d) E. Ruiz, S. Alvarez, J. Cano and V. Polo, *J. Chem. Phys.*, 2005, **123**, 164110.

13 F. Neese, *WIREs Comput Mol Sci*, 2012, **2**, 73–78.

14 a) K. Eichkorn, O. Treutler, H. Öhm, M. Häser and R. Ahlrichs, *Chem. Phys. Lett.*, 1995, **240**, 283–290, b) K. Eichkorn, O. Treutler, H. Öhm, M. Häser and R. Ahlrichs, *Chem. Phys. Lett.*, 1995, **242**, 652–660, c) K. Eichkorn, F. Weigend, O. Treutler and R. Ahlrichs, *Theor Chem Acta*, 1997, **97**, 119–124.

15 S. Vancoillie, J. Chalupský, U. Ryde, E. I. Solomon, K. Pierloot, F. Neese and L. Rulíšek, *J. Phys. Chem. B*, 2010, **114**, 7692–7702.

16 W. H. Harman, T. D. Harris, D. E. Freedman, H. Fong, A. Chang, J. D. Rinehart, A. Ozarowski, M. T. Sougrati, F. Grandjean, G. J. Long, J. R. Long and C. J. Chang, *J. Am. Chem. Soc.*, 2010, **132**, 18115–18126.

17 E. Ermilov, C. Oelsner, F. Birke, D. Gerber, V. Buschmann, A. Devaux and R. Erdmann, *Rev. Sci. Inst*, 2020, **91**, 069502.

18 C. Lian, H.-L. Li and G.-Y. Yang, *Inorg. Chem.*, 2022, **61**, 11335–11341.

19 R. I. Maksimovskaya and G. M. Maksimov, *Coord. Chem. Rev.* 2019, **385**, 81–99.

20 W. Chen, H. Li, J. Song, Y. Zhao, P. Ma, J. Niu and J. Wang, *Inorg. Chem.*, 2022, **61**, 2076–2085.

21 N. Burzlaff In Advances in Inorganic Chemistry; *Academic Press*: Cambridge, MA, USA, **2008**; *60*, 101–165.

22 U. Kortz, A. Tézé and G. Hervé, *Inorg. Chem.*, 1999, **38**, 2038–2042.

23 C. Pichon, P. Mialane, A. Dolbecq, J. Marrot, E. Rivière, B. S. Bassil, U. Kortz, B. Keita, L. Nadjo and F. Sécheresse, *Inorg. Chem.*, 2008, **47**, 11120–11128.
24 Z. Zhang, E. Wang, Y. Qi, Y. Li, B. Mao and Z. Su, *Cryst. Growth Des*, 2007, **7**, 1305–1311.
25 H.-M. Zhang, Y.-G. Li, Y. Lu, R. Clérac, Z.-M. Zhang, Q. Wu, X.-J. Feng and E.-B. Wang, *Inorg. Chem.*, 2009, **48**, 10889–10891.
26 X.-B. Han, Y.-G. Li, Z.-M. Zhang, H.-Q. Tan, Y. Lu and E.-B. Wang, *J. Am. Chem. Soc.*, 2015, **137**, 5486–5493.
27 H. Lv, Y. Chi, J. van Leusen, P. Kögerler, Z. Chen, J. Bacsa, Y. V. Geletii, W. Guo, T. Lian and C. L. Hill, *Chem. Eur. J.*, 2015, **21**, 17363–17370.
28 J. M. Clemente-Juan, E. Coronado, J. R. Galán-Mascarós and C. J. Gómez-García, *Inorg. Chem.*, 1999, **38**, 55–63.
29 S.-T. Zheng, D.-Q. Yuan, H.-P. Jia, J. Zhang and G.-Y. Yang, *Chem. Commun.*, 2007, 1858–1860.
30 S.-T. Zheng, J. Zhang, J. M. Clemente-Juan, D.-Q. Yuan and G.-Y. Yang, *Angew. Chem. Int. Ed.*, 2009, **48**, 7176–7179.
31 X.-B. Han, C. Qin, X.-L. Wang, Y.-Z. Tan, X.-J. Zhao and E.-B. Wang, *Appl. Catal. B*, 2017, **211**, 349–356.
32 Y. Chen, Z.-W. Guo, Y.-P. Chen, Z.-Y. Zhuang, G.-Q. Wang, X.-X. Li, S.-T. Zheng and G.-Y. Yang, *Inorg. Chem. Front.*, 2021, **8**, 1303–1311.
33 W. Guo, H. Lv, J. Bacsa, Y. Gao, J. S. Lee and C. L. Hill, *Inorg. Chem.*, 2016, **55**, 461–466.
34 J. Goura, B. S. Bassil, X. Ma, A. Rajan, E. Moreno-Pineda, J. Schnack, M. Ibrahim, A. K. Powell, M. Ruben, J. Wang, L. Ruhlmann and U. Kortz, *Chem. Eur. J.*, 2021, **27**, 15081–15085.
35 S. Cao and L. Piao, *Angew. Chem. Int. Ed.*, 2020, **59**, 18312–18320.
36 K. von Allmen, R. Moré, R. Müller, J. Soriano-López, A. Linden and G. R. Patzke, *ChemPlusChem*, 2015, **80**, 1389–1398.
37 H. Lv, J. Song, H. Zhu, Y. V. Geletii, J. Bacsa, C. Zhao, T. Lian, D. G. Musaev and C. L. Hill, *J. Catal.*, 2013, **307**, 48–54.
38 Z. Zhang, Q. Lin, S.-T. Zheng, X. Bu and P. Feng, *Chem. Commun.*, 2011, **47**, 3918–3920.
39 G.-H. Zhang, W.-B. Yang, W.-M. Wu, X.-Y. Wu, L. Zhang, X.-F. Kuang, S.-S. Wang and C.-Z. Lu, *J. Catal.*, 2019, **369**, 54–59.
40 G. Paille, A. Boulmier, A. Bensaid, M.-H. Ha-Thi, T.-T. Tran, T. Pino, J. Marrot, E. Rivière, C. H. Hendon, O. Oms, M. Gomez-Mingot, M. Fontecave, C. Mellot-Draznieks, A. Dolbecq and P. Mialane, *Chem. Commun.*, 2019, **55**, 4166–4169.
41 J. Zhao, Y. Ding, J. Wei, X. Du, Y. Yu and R. Han, *Int. J. Hydrog. Energy*, 2014, **39**, 18908–18918.
42 R. Liu, X. Shang, C. Li, X. Xing, X. Yu, G. Zhang, S. Zhang, H. Cao and L. Bi, *Int. J. Hydrog. Energy*, 2013, **38**, 9954–9960.
43 W. Wu, T. Teng, X.-Y. Wu, X. Dui, L. Zhang, J. Xiong, L. Wu and C.-Z. Lu, *Catal. Commun.*, 2015, **64**, 44–47.
44 H. Lv, Y. Gao, W. Guo, S. M. Lauinger, Y. Chi, J. Bacsa, K. P. Sullivan, M. Wieliczko, D. G. Musaev and C. L. Hill, *Inorg. Chem.*, 2016, **55**, 6750–6758.
45 W. Wu, X.-Y. Wu and C.-Z. Lu, *Catal. Commun.*, 2018, **114**, 56–59.
46 M. A. Fashapoyeh, M. Mirzaei, H. Eshtiagh-Hosseini, A. Rajagopal, M. Lechner, R. Liu and C. Streb, *Chem. Commun.*, 2018, **54**, 10427–10430.
47 R. Ban, Y. Liang, P. Ma, D. Zhang, J. Niu and J. Wang, *Inorg. Chem. Commun.*, 2016, **71**, 65–67.
48 a) X. Xu, X. Liu, D. Wang, X. Liu, L. Chen and J. Zhao, *Inorg. Chem.*, 2021, **60**, 1037–1044, b) E. Tanuhadi, E. Al-Sayed, G. Novitchi, A. Roller, G. Giester and A. Rompel, *Inorg. Chem.*, 2020, **59**, 8461–8467.
49 R. G. Finke, M. W. Droege and P. J. Domaille, *Inorg. Chem.*, 1987, **26**, 3886–3896.
50 R. Massart, R. Contant, J. M. Fruchart, J. P. Ciabrini and M. Fournier, *Inorg. Chem.*, 1977, **16**, 2916–2921.
51 X. López, J. A. Fernández and J. M. Poblet, *Dalton Trans.*, 2006, 1162–1167.
52 Y. C. Li, H. Z. Zhong, R. Li, Y. Zhou, C. H. Yang and Y. F. Li, *Adv. Funct. Mater.*, 2006, **16**, 1705–1716.

53 I. M. Mbomekalle, B. Keita, M. Nierlich, U. Kortz, P. Berthet and L. Nadjo, *Inorg. Chem.*, 2003, **42**, 5143–5152.
54 M. Ibrahim, Y. Xiang, B. S. Bassil, Y. Lan, A. K. Powell, P. de Oliveira, B. Keita and U. Kortz, *Inorg. Chem.*, 2013, **52**, 8399–8408.
55 a) M. Fondo, N. Ocampo, A. M. García-Deibe, J. Cano and J. Sanmartín, *Dalton Trans.*, 2010, **39**, 10888–10899, b) Y. Nishida and S. Kida, *J. Chem. Soc., Dalton Trans.*, 1986, 2633–2640, c) V. McKee, M. Zvagulis and C. A. Reed, *Inorg. Chem.*, 1985, **24**, 2914–2919, d) V. McKee, M. Zvagulis, J. V. Dagdigian, M. G. Patch and C. A. Reed, *J. Am. Chem. Soc.*, 1984, **106**, 4765–4772, e) L. Gutierrez, G. Alzuet, J. A. Real, J. Cano, J. Borrás and A. Castiñeiras, *Inorg. Chem.*, 2000, **39**, 3608–3614, f) L. Gutierrez, G. Alzuet, J. A. Real, J. Cano, J. Borrás and A. Castiñeiras, *Eur. J. Inorg. Chem.*, 2002, **2002**, 2094–2102, g) L. K. Thompson, S. S. Tandon, F. Lloret, J. Cano and M. Julve, *Inorg. Chem.*, 1997, **36**, 3301–3306.
56 a) L. K. Thompson, S. S. Tandon, F. Lloret, J. Cano and M. Julve, *Inorg. Chem.*, 1997, **36**, 3301–3306; b) E. Ruiz, P. Alemany, S. Alvarez and J. Cano, *Inorg. Chem.*, 1997, **36**, 3683–3688.
57 a) D. Luneau, C. Stroh, J. Cano and R. Ziessel, *Inorg. Chem.*, 2005, **44**, 633–637; b) N. Marino, D. Armentano, G. D. Munno, F. Lloret, J. Cano and M. Julve, *Dalton Trans.*, 2015, **44**, 11040–11051.
58 J. S. Miller and M. Drillon, Eds., *Magnetism: Molecules to Materials V*, Wiley, 1st edn., 2004.
59 a) D. Maganas, J. Krzystek, E. Ferentinos, A. M. Whyte, N. Robertson, V. Psycharis, A. Terzis, F. Neese and P. Kyritsis, *Inorg. Chem.*, 2012, **51**, 7218–7231, b) J. Telser, A. Ozarowski and J. Krzystek, in *Electron Paramagnetic Resonance*, eds. B. C. Gilbert, D. M. Murphy and V. Chechik, Royal Society of Chemistry, Cambridge, 2012, vol. 23, pp. 209–263.
60 S. Alvarez, *Chem. Rev.*, 2015, **115**, 13447–13483.
61 M. E. Rose, *Elementary Theory of Angular Momentum*; Courier Corporation, 1995.
62 a) D. F. Evans, *J. Chem. Soc.*, 1959, 2003–2005, b) C. Piguet, *J. Chem. Educ.*, 1997, **74**, 815-816.
63 L.-H. Bi, B. Li, L.-X. Wu and Y.-Y. Bao, *Inorg. Chim. Acta*, 2009, **362**, 3309–3313.
64 A. Haider, B. S. Bassil, Z. Lin, X. Ma, P. J. Haferl, J. K. Bindra, J. Kinyon, G. Zhang, B. Keita, N. S. Dalal and U. Kortz, *Dalton Trans.*, 2021, **50**, 3923–3930.
65 E. Al-Sayed, S. P. Nandan, E. Tanuhadi, G. Giester, M. Arrigoni, G. K. H. Madsen, A. Cherevan, D. Eder and A. Rompel, *ChemSusChem*, 2021, **14**, 2529–2536.
66 a) J. J. Stracke and R. G. Finke, *ACS Catal.*, 2013, **3**, 1209–1219, b) S. J. Folkman and R. G. Finke, *ACS Catal.*, 2017, **7**, 7–16, c) S. J. Folkman, J. Soriano-Lopez, J. R. Galán-Mascarós and R. G. Finke, *J. Am. Chem. Soc.*, 2018, **140**, 12040–12055.
